# Genetic background sets the trajectory of experimental cancer evolution

**DOI:** 10.1038/s41586-026-10821-z

**Published:** 2026-07-22

**Authors:** Sarah J. Aitken, Frances Connor, Christine Feig, Tim F. Rayner, Margus Lukk, Juliet Luft, Stuart Aitken, Claudia Arnedo-Pac, James F. Hayes, Michael D. Nicholson, Ailith Ewing, Vasavi Sundaram, Jan C. Verburg, John Connelly, Craig J. Anderson, Mikaela Behm, Susan Campbell, Maëlle Daunesse, Vera B. Kaiser, Elissavet Kentepozidou, Oriol Pich, Aisling M. Redmond, Javier Santoyo-Lopez, Inés Sentís, Lana Talmane, Liver Cancer Evolution Consortium, Sarah J. Aitken, Frances Connor, Christine Feig, Tim F. Rayner, Margus Lukk, Juliet Luft, Stuart Aitken, Claudia Arnedo-Pac, Ailith Ewing, Vasavi Sundaram, Jan C. Verburg, John Connelly, Craig J. Anderson, Maëlle Daunesse, Vera B. Kaiser, Elissavet Kentepozidou, Oriol Pich, Inés Sentís, Lana Talmane, Ruben M. Drews, Paul A. Ginno, Erika López-Arribillaga, Paul Flicek, Núria López-Bigas, Colin A. Semple, Martin S. Taylor, Duncan T. Odom, Paul Flicek, Núria López-Bigas, Colin A. Semple, Martin S. Taylor, Duncan T. Odom

**Affiliations:** 1 Center of Molecular and Cellular Oncology, Yale Cancer Center, New Haven, CT USA; 2 Department of Pathology, Yale School of Medicine, Yale University, New Haven, CT USA; 3 Medical Research Council Toxicology Unit, University of Cambridge, Cambridge, UK; 4 Cancer Research UK Cambridge Institute, University of Cambridge, Cambridge, UK; 5 Department of Histopathology, Cambridge University Hospitals NHS Foundation Trust, Cambridge, UK; 6 Medical Research Council Human Genetics Unit, Institute of Genetics and Cancer, University of Edinburgh, Edinburgh, UK; 7 Institute for Research in Biomedicine (IRB Barcelona), The Barcelona Institute of Science and Technology, Barcelona, Spain; 8 School of Mathematics and the Maxwell Institute for Mathematical Sciences, University of Edinburgh, Edinburgh, UK; 9 European Molecular Biology Laboratory, European Bioinformatics Institute, Hinxton, UK; 10 Edinburgh Pathology, Institute of Genetics and Cancer, University of Edinburgh, Edinburgh, UK; 11 Laboratory Medicine, NHS Lothian, Edinburgh, UK; 12 Division of Regulatory Genomics and Cancer Evolution (B270), German Cancer Research Center (DKFZ), Heidelberg, Germany; 13 Edinburgh Genomics (Clinical), University of Edinburgh, Edinburgh, UK; 14 Institute of Systems Genomics, University of Connecticut, Storrs, CT USA; 15 Universitat Pompeu Fabra (UPF), Barcelona, Spain; 16 Institució Catalana de Recerca i Estudis Avançats (ICREA), Barcelona, Spain

**Keywords:** Genome informatics, Cancer models, Cancer genomics, DNA damage and repair, Experimental evolution

## Abstract

Human cancers are heterogeneous^1^. Dissecting how germline genetic variation and environmental factors shape tumour evolution using human datasets is limited by inherent diversity in genetic backgrounds^2^ and environmental exposures^3–5^. Here, to overcome these limitations, we re-ran early tumour evolution hundreds of times in diverged inbred mouse strains, generating matched histology and whole-genome and transcriptome sequences. The sex, environment and carcinogenic exposures were all controlled, and the study design allowed us to capture genetic variation comparable with that observed across human populations while exploiting the nested hierarchical structure of strain–litter–animal–tumour relationships. Our analyses reveal that epistatic interactions between genetic background and acquired somatic mutations result in population-specific disease progression, including choice of driver mutations, occurrence of whole-genome duplication and subclonal selection dynamics that mirror both cancer susceptibility and tumour growth rate. Even modest genetic divergence, comparable with that found across human ancestry groups, can strikingly alter selection pressures during cancer development to shape both cancer risk and the trajectory of tumour evolution.

## Abstract (web summary)

Experimentally replaying tumour evolution in divergent mouse strains reveals the importance of interactions between genetic ancestry and acquired cancer-driving mutations in shaping the earliest stages of cancer development.

## Main

Among the most critical and yet least understood stages of cancer formation are the earliest. We lack a comprehensive account of how normal cells acquire driver mutations and interact with environmental factors to achieve oncogenic transformation^6–9^. Retrospective analysis of heterogeneous human tumours has inferred that a few common drivers initiate most cancers^10^. In addition to well-established environmental risk factors^11,12^, mutational signature analyses^3^ have identified putative causative exposures^13^, but these only partly explain differences in cancer incidence between populations^4,14^ and do not account for ancestry differences^2,15^.

People with inherited cancer predisposition syndromes enable molecular profiling of multiple tumours arising under shared genetic and environmental conditions within one individual^16,17^. However, the generalizability of their tumour evolution is limited by small numbers of patients and profiled cancers, and confounded when such strong genetic predisposition can dominate environmental risk factors^18,19^. More generally, human studies are hampered by the polygenic diversity of populations, or by small sample numbers in families with predisposition syndromes—both of which are limited by poorly recorded histories of lifestyle risk factors. As a consequence, it is not clear how consistently cancer would develop given the same genetic background and the same environment, or the mechanisms by which polygenic germline differences influence the trajectory to tumorigenesis.

We reasoned that a powerful strategy to understand the mechanisms of tumorigenesis would be to implement Gould’s thought experiment of replaying evolution^20^, in which cancer development is experimentally repeated hundreds of times for comprehensive analysis. Such a prospective and in vivo approach to study early cancer development would control for many genetic and environmental variables that complicate human studies. Mutagenesis of the mouse liver by the DNA-damaging mutagen diethylnitrosamine (DEN) is a highly controlled system of in vivo tumorigenesis that reproducibly creates hepatocyte-derived tumours that histologically mimic human hepatocellular carcinoma^21^. DEN is metabolically activated by centrilobular hepatocytes to form well-understood mutagenic DNA adducts^22^. Following a single DEN exposure 15 days after birth, C3H/HeOuJ male mice reliably develop multiple clonally distinct tumours within 25 weeks^23,24^, each typically harbouring 60,000 base substitution mutations^25^. A discrete exposure to DEN results in pronounced chromosome-scale mutational asymmetry through the process of lesion segregation, because tumours develop from the clonal growth of cells containing damage on only one strand of the DNA duplex^25^. These strand-oriented mutations are a powerful tool to analyse tumour clonal dynamics, selection, homologous recombination and DNA repair^25–28^, which are generalizable to human contexts^25,29–31^.

**Fig. 1:**
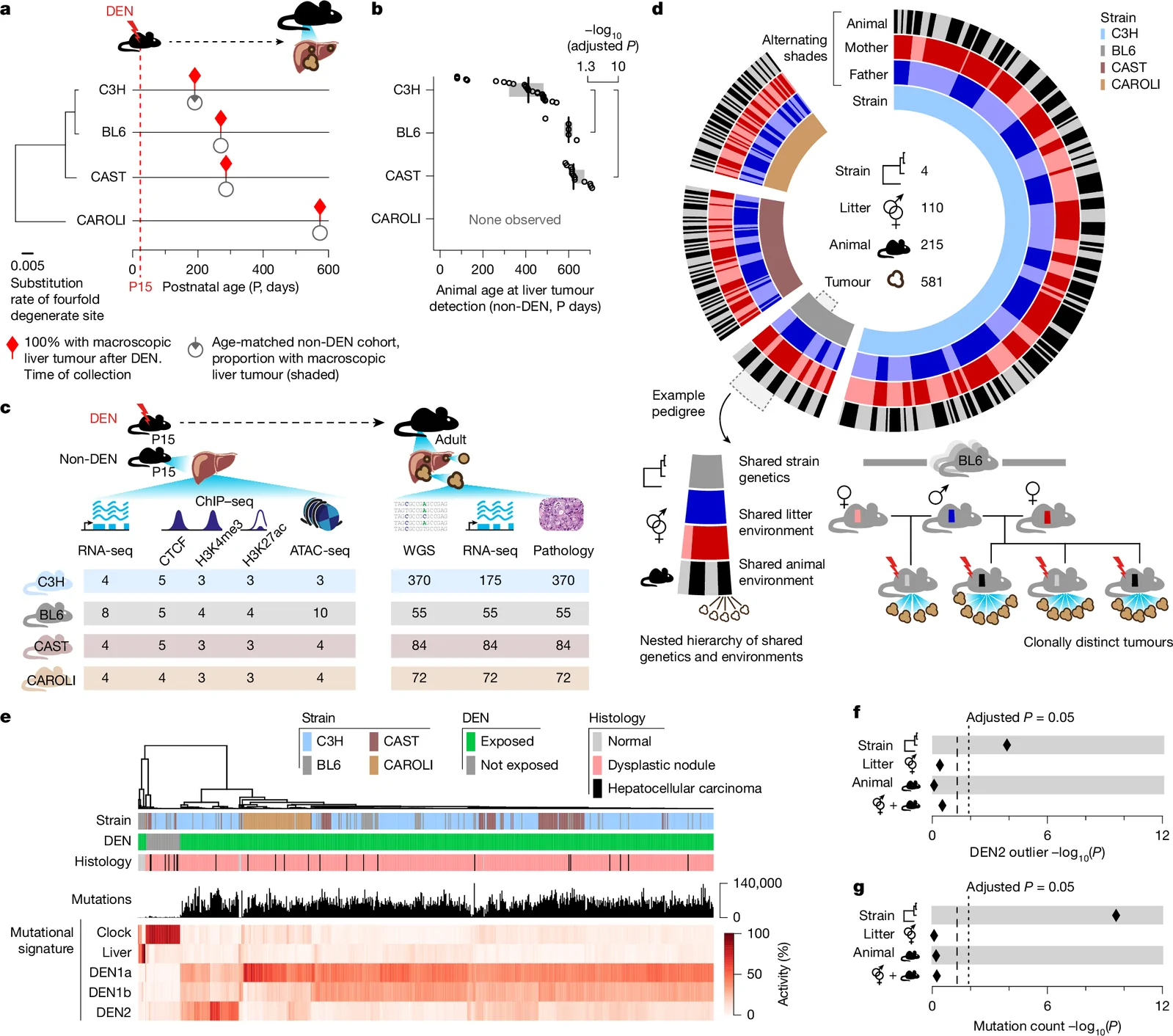
Cancer susceptibility and mutagenesis are shaped by germline genetic variation. **a**, Summary of tumour induction by DEN and tumour latency in each mouse strain. Phylogenetic tree branch lengths are substitution rates of protein-coding fourfold degenerate sites. DEN-induced tumours were collected at fixed timepoints (*x* axis) for each strain (red diamonds), corresponding to 100% tumour incidence (Methods). The grey lollipops show the proportion (shaded area) of untreated, age-matched mice that developed spontaneous tumours at the same timepoint (tumours detected in only C3H at age-matched timepoints). **b**, Timing of spontaneous tumours in untreated mice from prolonged colony surveillance (Supplementary Table 3). The medians (black bar) and interquartile ranges (grey box) are shown. *P* values were determined using a Kolmogorov–Smirnov test after Bonferroni correction; only *P* < 0.05 after correction is indicated. **c**, Summary of the study design and resulting datasets (Supplementary Table 2). **d**, Nested hierarchical relationships of tumour samples enabling analysis of genetic and shared environmental components of tumour development. Each mother contributed one litter, so mother identity defines the litter identity. **e**, Nucleotide substitution mutation rates and signatures were calculated for each WGS sample (columns, total *n* = 647, including non-DEN exposed and histologically normal samples for comparison). Mutational signatures were deconvolved (Methods) to define five component signatures, three associated with DEN exposure: DEN1a, DEN1b and DEN2 (Extended Data Fig. 1e). **f**, In likelihood ratio tests of nested mixed-effects models, the frequency of outlier tumours with more than 30% DEN2 signature mutations shows significant association with strain, but not litter or animal (the dashed line denotes *P* = 0.05, and the dotted line indicates the Bonferroni-corrected *P* = 0.05). **g**, Differences in nucleotide substitution mutation rate are attributed to strain, whereas shared litter, animal and combined litter + animal effects are not significant. Plotting and statistics are as in **f**. Source data

Using this DEN system, we re-ran tumorigenesis in four inbred mouse strains and species (Fig. 1), generating matched genomes, transcriptomes and histology for hundreds of tumours for comparative analysis. The natural genetic variation of these evolutionarily divergent mice captures genetic variation equal to or exceeding that of pairs of individuals selected from diverged human ancestry groups^32,33^, diversity that has been historically neglected in population-level cancer studies^15^. Our analyses revealed strong genetic background influences on genome stability and the intensity of oncogenic selection, and multiple instances of epistatic germline–somatic interactions that shaped subsequent cancer evolution.

## Germline differences in tumour latency

We chemically induced liver tumours^24,25^ by injecting a single intraperitoneal dose of DEN on postnatal day 15 (P15) into inbred male mice of the following genetic backgrounds: *Mus musculus musculus* (*n* = 104 C3H/HeOuJ and *n* = 12 C57BL/6J), *M. musculus castaneus* (*n* = 54 CAST/EiJ) and *Mus caroli* (*n* = 45), hereafter referred to as C3H, BL6, CAST and CAROLI, respectively (Fig. 1a). For simplicity, these strains, subspecies and species are subsequently referred to as strains. The resultant tumours (*n* = 581; Supplementary Table 1) were dissected and processed in parallel for comprehensive profiling using whole-genome sequencing (WGS), total transcriptome sequencing (RNA sequencing (RNA-seq)) and histopathological analysis (Supplementary Table 2). Additional spontaneous liver tumours from a cohort of aged, untreated animals were collected and processed in parallel for comparison (Fig. 1b and Supplementary Table 3). Detailed characterization of the transcriptional state and regulatory landscape of normal liver tissue from untreated P15 mice (the developmental time of DEN exposure) was performed using total RNA-seq, assay for transposase-accessible chromatin using sequencing (ATAC-seq) and chromatin immunoprecipitation followed by high-throughput sequencing (ChIP–seq; Fig. 1c).

We measured a marked variation in susceptibility to DEN-induced tumorigenesis between strains (Fig. 1a). Following treatment, tumours were consistently present after 25 weeks (P190) in C3H but not until 36 weeks (P267) in BL6, in agreement with previous studies^23^. For the first time, to our knowledge, we performed a comprehensive analysis of the DEN model in CAST and CAROLI, which revealed further extended latencies: 38 weeks (P281) in CAST and 78 weeks (P561) in CAROLI. Spontaneous tumours in untreated mice followed the same trend, with C3H most susceptible, then BL6, CAST and CAROLI progressively more resistant (Fig. 1a,b and Supplementary Table 3).

**Extended Data Fig. 1:**
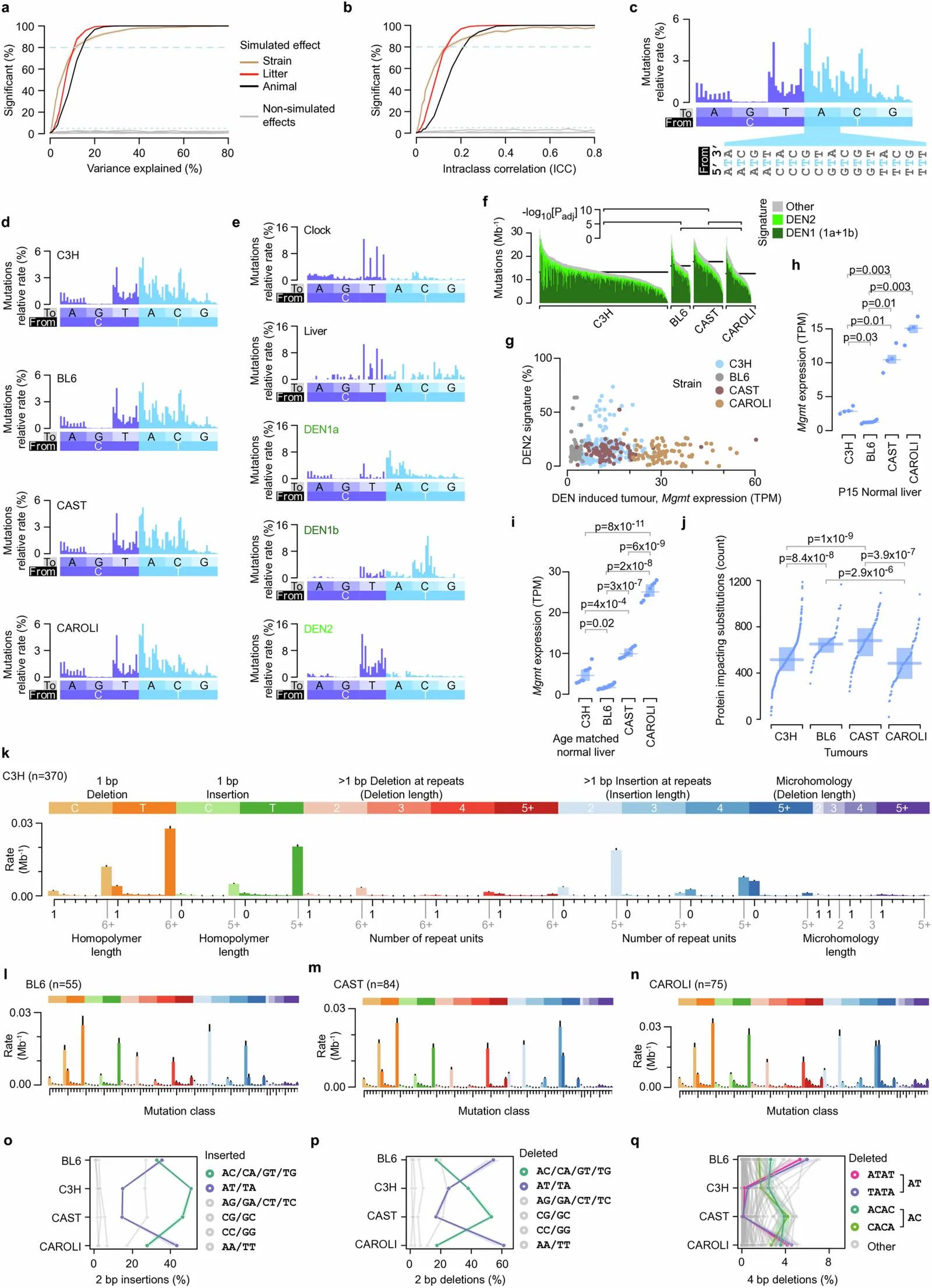
Statistical power and mutation signatures. **a**, Continuous data simulated using the study specific nested data structure (Methods) shows good power to detect simulated effects (strain, litter or animal) using a likelihood ratio test of nested mixed effects models (tan, red and black curves; consistently exceed 80% (dashed blue line) for ≥ 20% explained variance). False discovery of non-simulated effects is well controlled (grey curves consistently below p = 0.05 significance threshold; y-axis, dotted blue line), including the null model where there is no simulated nested effect (x = 0). Percentage significance from 1,000 simulations, 95% CI (shaded areas, just visible) from bootstrap of the simulations. **b**, Simulation and testing of categorical rather than continuous data (Methods). Effect size is shown as the simulated intraclass correlation (ICC, x-axis) for the simulated effect (strain, litter or animal). Analyses consistently exceed 80% power (dashed blue line) for ICC ≥ 20% and false discovery of non-simulated effects is well controlled. **c**, Base substitution mutation rates calculated for DEN-induced tumours aggregated for all strains. Substitution rates were calculated, stratifying by the substitution type and conditioned on the upstream and downstream nucleotide. Rates were calculated as the number of mutations divided by the number of trinucleotide occurrences, allowing direct comparison between genomes. Histograms were normalised so the total area of each histogram sums to 100%. Reverse complement substitutions were combined and are shown relative to substitutions from the pyrimidine nucleotide (e.g. ATG- > ACG and CAT- > CGT combined and shown as the former). **d**, As for panel **c**, calculated separately for each strain. **e**, Mutation signature deconvolution identifies five distinct signatures present amongst the 647 analysed genomes. The signature labelled Clock is predominant in non-DEN exposed (spontaneous) tumours (Fig. 1e) and closely matches expectations for a combination of the established human clock-like mutation signatures SBS1 and SBS5 (ref. ^3^). The signature labelled Liver is predominant in DEN-exposed, histologically normal samples which are also typified by low mutation rates (Fig. 1e). The remaining signatures are identified in DEN-exposed samples. Previously signatures DEN1 and DEN2 have been described for DEN-induced C3H liver tumours^25^, here the DEN1 signature has split into DEN1a and DEN1b to capture the differences in the DEN-induced mutation profile between CAROLI and the other strains, CAROLI demonstrating a reduction in T- > C mutation (lower level of signature DEN1b). **f**, Genome wide substitution mutation rate distribution for each strain. Despite greater tumour susceptibility, C3H does not have a systematically higher rate of mutations. Horizontal line denotes the median mutation rate per strain and pairwise significance of differences in mutation rate are indicated, only significant differences shown (two-sided Mann-Whitney tests with Bonferroni correction). **g**, For each tumour with RNA-seq based measures of gene expression, the normalised (TPM) level of *Mgmt* expression (x-axis) is plotted against the percentage of mutations attributed to the DEN2 signature (y-axis). **h**, Transcript expression level of the DNA repair gene *Mgmt* in P15 liver tissue differs between strains; one point per sample, strain-median is the horizontal line and shaded box shows inter-quartile range. Expression shown in transcripts per million (TPM). Significant (p < 0.05, two-sided Student’s t-test test) pairwise differences shown with Bonferroni corrected p-value. **i**, *Mgmt* expression measured in normal (non-DEN exposed, non-tumour) tissue from animals at the same age as tumour collection for each strain. Plots and statistics as for panel **h**. **j**, There are typically 400 to 800 protein impacting (non-synonymous, stop gain, stop loss or essential splice site) single nucleotide substitutions per DEN-induced tumour. Horizontal lines show medians, shaded boxes show the interquartile ranges. Significant (p < 0.05, two-sided Mann-Whitney test) pairwise differences are shown with Bonferroni corrected p-value. **k**, Insertion and deletion (indel) mutation profile for DEN-induced C3H tumours. Mutations are shown as rates per Mb of genome sequence (y-axis), whiskers denote 95% confidence intervals from bootstrap sampling tumours. Eighty-three categories of indel mutation are defined (x-axis) stratifying by insertion/deletion, event length, and local sequence repetitiveness. The x-axis scale annotates the first (black) and last (grey) repeat measure for the colour coded groups of mutation category, intervening ticks are monotonically increasing. **l**, BL6 indel mutations plotted as **k**. **m**, CAST indel mutations plotted as **k**. **n**, CAROLI indel mutations plotted as **k**. **o**, The sequence composition of inserted dinucleotides differs between mouse strains, with AT (purple) the most inserted in BL6 and CAROLI and AC (green) in C3H and CAST; clustering does not reflect the phylogeny of the strains. Lines connect corresponding points between strains; shaded area denotes the 95% confidence interval. **p**, Dinucleotide deletions show similar strain biases to insertions. Plotted as **o**. **q**, strain biases for 4-nucleotide deletions show the bias for BL6 and CAROLI to delete AT dinucleotide containing sequences extends to larger deletions. Plotted as for **o-p** but considering all possible 4-nucleotide sequences.

With multiple clonally independently DEN-induced tumours per animal, multiple litters per strain and known pedigrees (Fig. 1d and Supplementary Table 1), our experimental design is well structured and powered to dissect the separate influences of both genetics and shared environment using mixed-effects models (Extended Data Fig. 1a,b). Collectively, these data constitute a unique and highly controlled resource to study the molecular mechanisms and clonal dynamics underlying early tumorigenesis in vivo.

## Strain influences genome stability

We explored what underlying patterns of mutagenesis and genome stability shape strain-specific latencies by comparing the somatic changes in WGS of DEN-induced tumours between the four strains (total *n* = 581 tumours). The type and sequence context of base substitution mutations (Extended Data Fig. 1c) broadly correspond to the DEN1 and DEN2 mutational signatures derived from C3H tumours alone^25^. However, mutational signature deconvolution reveals a phylogenetic shift within the DEN1 mutational signature, in which CAROLI has a decreased propensity for T→C mutations compared with the other strains (Extended Data Fig. 1d,e).

The DEN2 signature contributes a significant minority of the total base substitutions in all four strains, with a median of between 11.8% (CAROLI) and 14.1% (CAST), but there are notable outlier tumours in C3H and BL6, which have more than 30% mutations attributed to DEN2 (14% and 7% of tumours, respectively; Fig. 1e,f and Extended Data Fig. 1f,g). *O*^6^-ethG adducts are the likely source of the G→A (or reverse complement C→T) mutations that dominate the DEN2 signature^25^, and can be directly repaired by the enzyme *O*^6^-methylguanine DNA methyltransferase (MGMT). As each MGMT enzyme can repair only a single adduct^34^, this system can be saturated. *Mgmt* is expressed at much lower levels in C3H and BL6 than the other strains in normal tissue, including at the time of DEN exposure (Extended Data Fig. 1h,i), indicating that the outlier tumours with more than 30% DEN2 signature may represent cases where this direct repair system was overwhelmed, resulting in an excess of *O*^6^-ethG-induced mutagenesis.

Genome-wide base substitution burden varies considerably between mouse strains (Fig. 1g and Extended Data Fig. 1f; analysis of variance (ANOVA), *P* = 1.12 × 10^−13^), and is typically higher in CAST (median 17.6 Mb^−1^) and BL6 (median 16.6 Mb^−1^) than in C3H (median 13.5 Mb^−1^) and CAROLI (median 13.3 Mb^−1^). Although there is high variance of mutational rate within each strain, neither the shared litter nor animal environments in these inbred mice significantly contribute to mutational rate variation (Fig. 1g). As mutational burden is discordant with tumour latency, the high susceptibility of C3H is not simply explained by more mutations.

Small (less than 50 bp) insertion and deletion (indel) mutations are rare in DEN-induced tumours (1.4%, s.d. 1.05 of the rate of substitutions). Indels typically introduce fewer than 2 changes per tumour that alter amino acid sequences (mean 1.77, s.d. 0.99), whereas single-base substitutions introduce 400–800 such changes (Extended Data Fig. 1j). Between tumours, the rate of indel mutations is positively correlated with nucleotide substitution rate (Pearson’s correlation = 0.21, *P* = 3.9 × 10^−7^) and the ID83-style signature of indel mutations is highly conserved. Nevertheless, there are strain preferences in nucleotide composition of longer indels (Extended Data Fig. 1k–q).

## Strain determines propensity to WGD

**Fig. 2:**
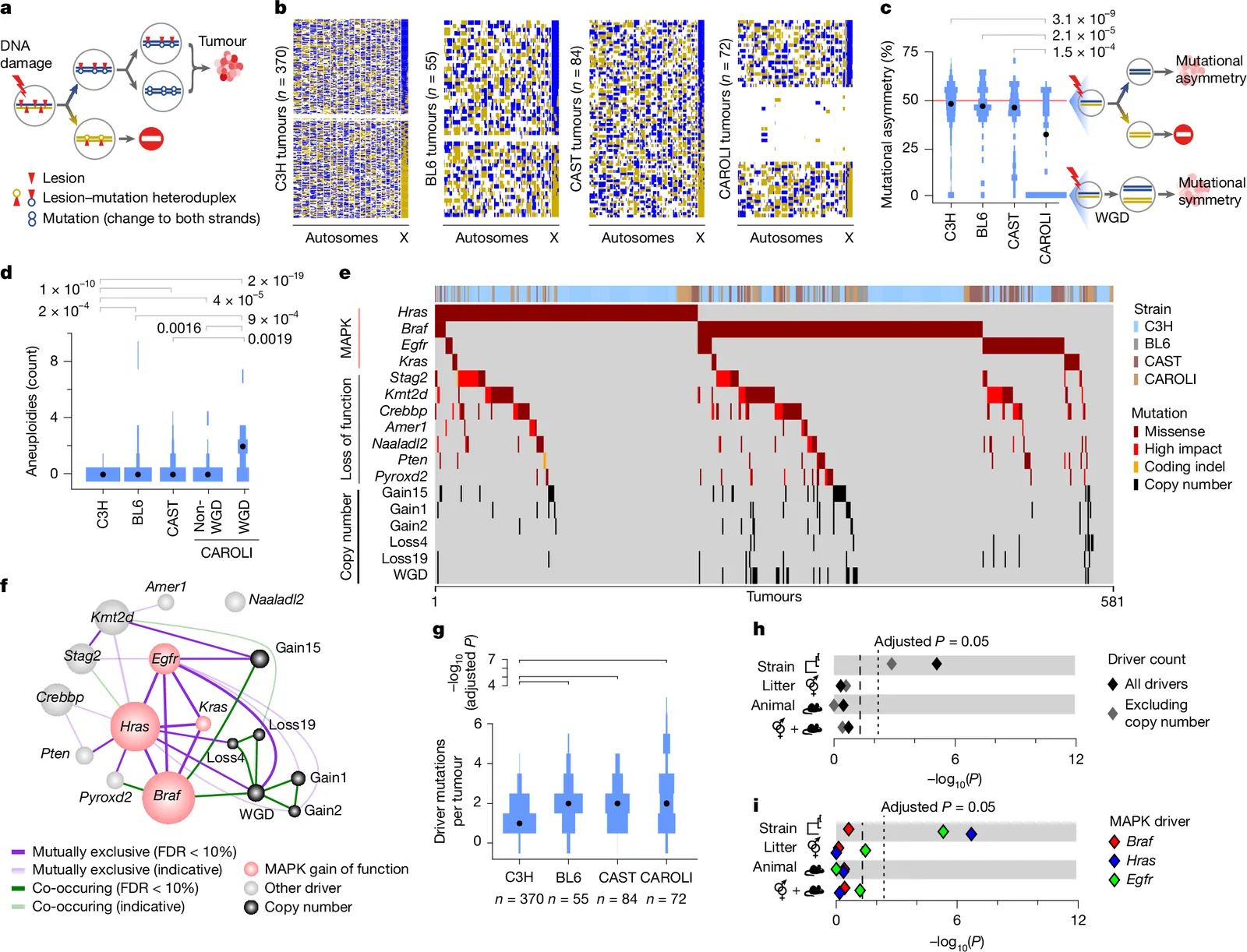
Profound strain differences in WGD but universal convergence on MAPK activation. **a**, Following mutagenic exposure, both DNA strands are damaged, but lesion segregation into daughter lineages and mutagenic replication over damage results in chromosome-scale mutational asymmetry in the tumours that develop. **b**, Mutational asymmetry profiles of individual tumours (rows) across the genomes (*x* axis) of each strain showing genomic segments with asymmetry (*S* > 0.33 in blue; *S* < −0.33 in gold) and without asymmetry (white). **c**, DEN-induced tumours typically show mutational asymmetry across 50% of their autosomal genome (median, circle). In CAROLI, the distribution is bimodal; 36% of tumours have no or negligible asymmetry. Differences indicated where *P* < 0.05, two-sided Mann–Whitney, Bonferroni corrected. The mutationally symmetric genomes could be explained by WGD in the first cell division following mutagenic insult (bottom schematic). **d**, There are fewer aneuploidies in C3H tumour genomes (*y* axis; median, circle) than the other strains, and more in WGD than in non-WGD CAROLI tumours. Differences indicated where *P* < 0.05, two-sided Mann–Whitney, Bonferroni corrected. **e**, Candidate driver mutations (rows) across the 581 DEN-induced tumours (columns). Stop–gain, stop–loss and essential splice-site mutations are annotated as high impact. **f**, Network graph summarizing the driver mutation interdependency relationships, illustrating the core oncogenic dependency on MAPK pathway gain-of-function mutations. The area of circles is proportional to the number of mutations. FDR, false discovery rate. **g**, The total number of driver mutations per tumour differs between strains (two-sided Mann–Whitney, Bonferroni corrected). **h**, The count of putative driver mutation events per tumour is associated with strain but does not show litter or animal effects. The dashed line denotes *P* = 0.05, and the dotted line indicates the Bonferroni-corrected *P* = 0.05. **i**, There are strain biases in the frequency of *Hras*-activating and *Egfr*-activating mutations, but no significant associations with litter or animal. Statistical analysis is as in panel **h**. Source data

The vast majority of C3H (97%), BL6 (94%) and CAST (100%) DEN-induced tumours demonstrate the characteristic mutational asymmetry of lesion segregation^25^. By contrast, over one-third (37%) of the CAROLI tumours appear mutationally symmetric (Fig. 2a–c). These symmetric CAROLI tumours are typified by a significantly higher mutational load than asymmetric tumours (mean 1.4-fold higher, *P* = 0.0014, two-sided Mann–Whitney *U*-test) and approximately one-half of the variant allele frequency (mean 1.8-fold lower, *P* = 5.8 × 10^−15^, two-sided Mann–Whitney *U*-test). Collectively, these observations are consistent with both daughter genomes of the originally mutagenized DNA duplex contributing equally to the sequenced tumour^26,28^. In other words, the complementary mutational asymmetry of the two daughter genomes, arising from the damaged DNA duplex, systematically cancel out each other.

**Extended Data Fig. 2:**
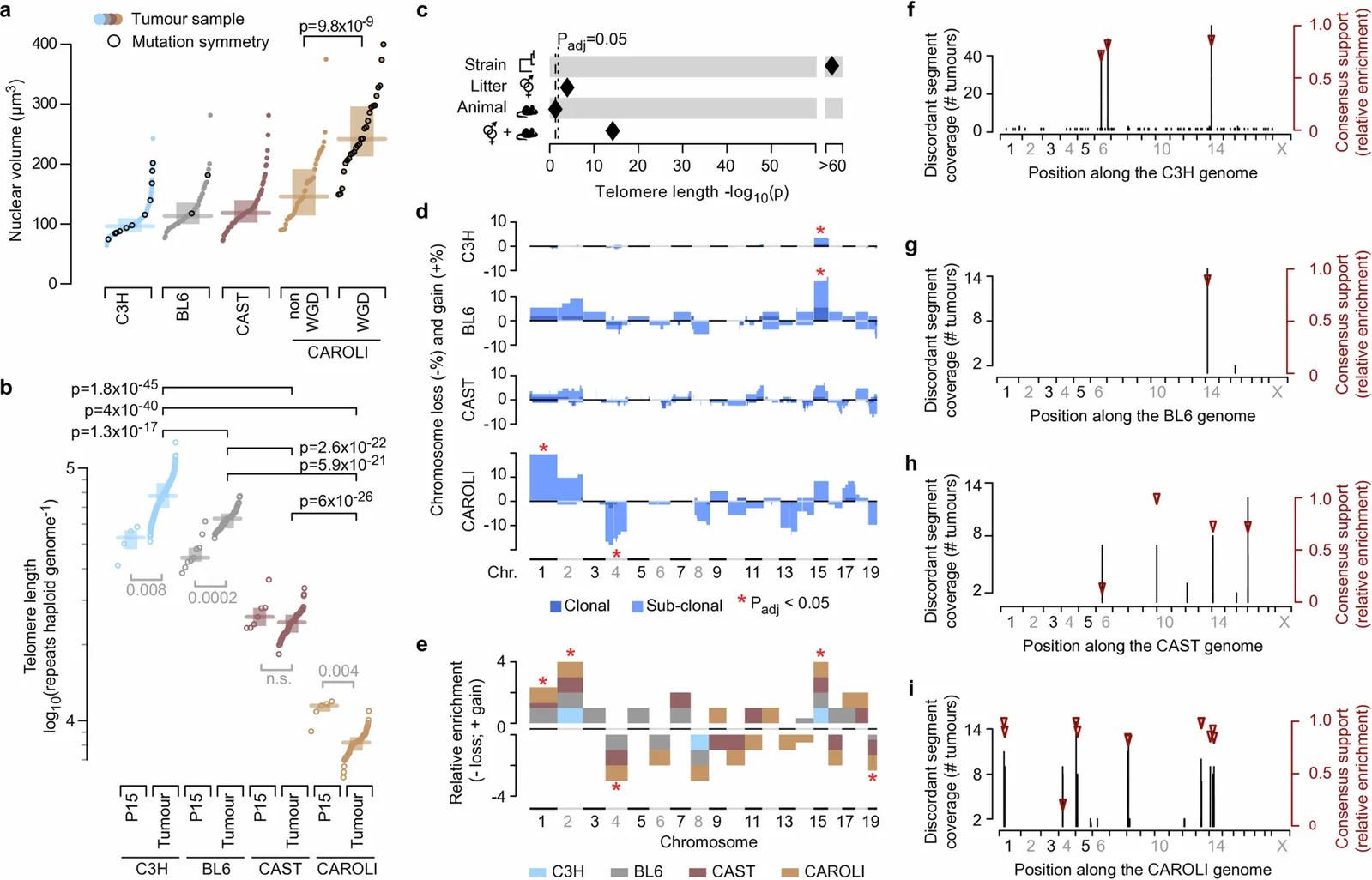
Identifying whole genome duplication, telomere dynamics, and reference genome misassemblies. **a**, Median nuclear volume per tumour (y-axis) calculated from histology shows that mutationally symmetric (black outline) CAROLI tumours have approximately double the nuclear volume of other tumours and significantly greater than non-mutationally symmetric (asymmetric) tumours (two-sided Mann-Whitney test). Boxes denote inter-quartile ranges and bars the median. **b**, Comparison of telomere lengths between P15 liver and DEN-induced tumours within strains (grey comparison bars). Comparisons of telomere lengths between DEN-induced tumours between strains (black comparison bars). In C3H and BL6 telomeres are long and get longer in the tumours. In CAROLI they are short at P15 and get shorter in the tumours. Statistical evaluation by two-sided Mann-Whitney tests with Bonferroni correction (factor=10). **c**, Likelihood ratio tests of nested mixed-effects models with telomere length as the response variable demonstrates association with strain but also significant associations with litter and litter+animal, indicating an element of parent to offspring telomere length inheritance. Bonferroni corrected p = 0.05 indicated (dotted line). **d**, Large (>10 Mb) chromosomal gains (+%) and losses (-%) show pronounced differences in frequency between strains and significant bias towards either gain or loss for some chromosomes (* Bonferroni corrected two tailed permutation test p-value < 0.05). **e**, Whether a chromosome is preferentially gained or lost (y-axis relative enrichment metric, summed over strains) is conserved between strains. Three chromosomes are significantly enriched for copy-gains and two for copy loss in aggregate analysis over strains (* Bonferroni corrected two tailed permutation test p-value < 0.05). **f**, Candidate C3H reference genome assembly errors (previously reported^25^). Immediate switches between forward and reverse strand asymmetry are not expected on autosomes unless both copies of the chromosome have a sister chromatid exchange (SCE) event at equivalent sites. However, inversions and translocations between the sequenced genomes and the reference assembly are expected to produce immediate asymmetry switches. The discordant segment coverage count (black y-axis) shows the number of informative tumours (those with either forward or reverse strand asymmetry at the corresponding genome position) that suggest a tumour genome to reference genome discrepancy. Consensus support (brown y-axis) plotted as triangles shows the relative enrichment of informative tumours that support a genomic discrepancy at the indicated position (values > 0 indicate consensus support for misassembly). **g-i**, Candidate reference genome assembly errors for BL6 (GRCm38/mm10), CAST and CAROLI, plotted as for **f**. The only candidate error in the high quality BL6 reference genome (GRCm38/mm10), located on chromosome 14, corresponds to a proposed assembly error at the approximate orthologous position on chromosome 14 in each of the C3H, CAST, and CAROLI assemblies, suggesting the BL6 misassembly has propagated through the later-assembled genomes. The full list of assembly errors is provided in Supplementary Table 4.

One possible mechanistic explanation for these symmetric genomes is whole-genome duplication (WGD) due to the failure of karyokinesis in the first mitotic division following mutagenesis, which would retain both pairs of sister chromatids in one nucleus (Fig. 2c). In CAROLI tumours, this is supported by the approximate doubling of nuclear volume (quantified from digitized histopathology; Extended Data Fig. 2a). By contrast, the rare mutationally symmetric tumours in other strains do not show increased nuclear size and probably represent instances in which both daughter lineages of an originally mutagenized cell have survived to contribute equally to the tumour^28^. This illustrates profound germline differences in WGD propensity during tumorigenesis.

Short and damaged telomeres have been mechanistically implicated in WGD^35^ and altered cancer risk^36^. In untreated P15 livers, CAROLI have significantly shorter telomeres than the other strains (Extended Data Fig. 2b) and, uniquely among the strains, telomere length in CAROLI tumours is shorter than in the corresponding P15 liver (Extended Data Fig. 2b). This suggests that a genetic predisposition to telomere crisis is mechanistically linked to damage-induced WGD. In addition to the strong strain effect, telomere length shows significant, albeit weaker, associations among tumours from the same animal and litter, supporting previous evidence of telomere length inheritance^37^ (Extended Data Fig. 2c).

Tumours in CAROLI mice were also outliers in several other facets of genome integrity, including the enrichment of aneuploidies and copy number alterations (Fig. 2d, Extended Data Fig. 2d,e and Supplementary Note), and depletion of sister chromatid exchanges (Supplementary Note) identified after removing artefacts from reference genome misassemblies (Extended Data Fig. 2f–i and Supplementary Table 4).

Thus, genetic background substantially influences genome stability following DEN treatment at multiple scales, from single-base substitutions to WGDs. However, none of these strain differences in mutational mechanisms obviously explains the observed differences in tumour susceptibility. Therefore, we next identified and compared the oncogenic driver events.

## Conserved selection of driver pathway

We identified genes containing candidate driver mutations using a suite of complementary driver discovery approaches (see Methods) using 581 tumours aggregated across all strains. Remarkably, 95% of tumours have probable activating mutations in MAPK pathway genes: *Braf* (*n* = 252), *Hras* (*n* = 224), *Egfr* (*n* = 84) or *Kras* (*n* = 21; Fig. 2e). Seven additional genes were under positive selection (Fig. 2e). Of these, *Pten*, *Crebbp* and *Amer1* are well-established tumour suppressor genes in the PI3K, Notch and WNT signalling pathways^38^, and *Stag2* and *Kmt2d* are tumour suppressor genes involved in chromatin regulation. Both *Stag2* and *Amer1* are located on the X chromosome, so their loss of function was a complete knockout in the all male mice of this study. The other identified candidates are *Pyroxd2* and *Naaladl2*, neither of which are known cancer driver genes.

**Extended Data Fig. 3:**
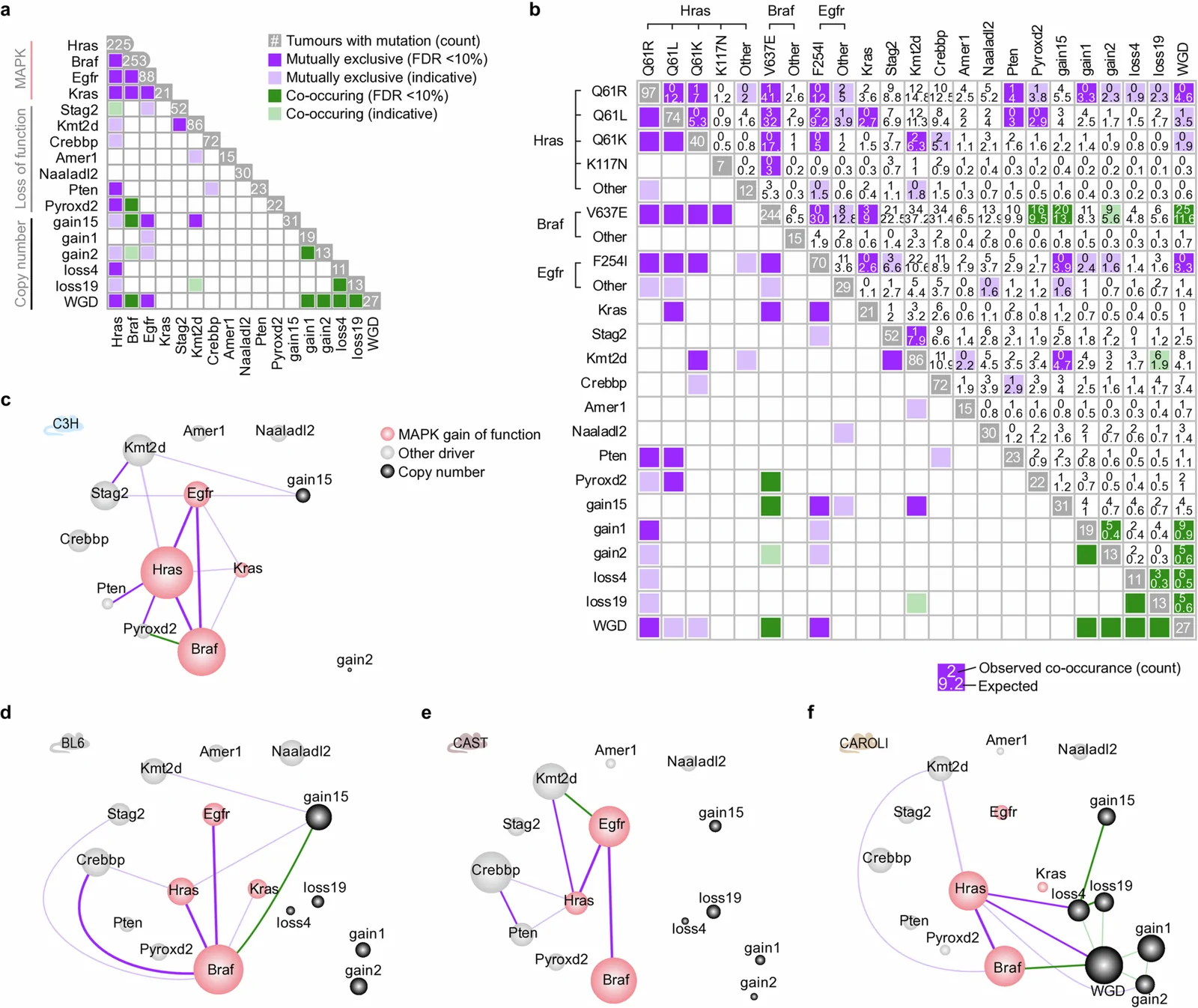
Driver mutation occurrence and interdependency. **a**, Driver mutation co-occurrence (green) and mutual exclusivity (purple) analysis, for example indicating oncogenic synergy between *Braf* activating mutations and loss of function in *Pyroxd2*. Aggregate analysis encompassing all strains. Statistical analysis by permutation^80^. Lighter shading denotes trend towards co-occurrence (green) and mutual exclusivity (purple). **b**, Extended version of multi-strain analysis in panel **a**, separating out specific highly recurrent driver mutations within the same gene. “Other” denotes other heterogeneous protein impacting changes in a given gene; these show less strong co-occurrence and mutual exclusivity relationships indicating they may be mixed populations of both driver and non-driver mutations. Observed and expected co-occurrence counts are indicated in the upper triangle. **c-f**, Occurrence and interdependency graph for driver mutations in each strain. The area of each node is linearly scaled to the proportion of driver mutations, edges show co-occurrence and mutual exclusivity relationships identified and are coloured as in **a-b**. There is a high degree of consistency between strains in the driver mutations present and their co-occurrence/exclusivity relationships, sbut pronounced distortions in their relative frequencies.

The activating mutations in the MAPK pathway show strong mutual exclusivity that is consistently replicated across strains (Fig. 2e,f and Extended Data Fig. 3). Other candidate driver mutations show patterns of co-occurrence and mutual exclusivity that relate to the specific MAPK gene mutated and define distinct axes of oncogenic pathway perturbation (Fig. 2f and Extended Data Fig. 3). These driver relationships include significant co-occurrence of *Braf* mutations with gain of *Myc*-containing chromosome 15 (gain15; weighted mutual information (wMI) *P* = 0.015), mutual exclusivity of *Stag2* and *Kmt2d* (wMI *P* < 0.001) and a consistent trend for *Kmt2d* mutual exclusivity with *Hras* (Fig. 2f and Extended Data Fig. 3a).

It is striking that in CAROLI, WGD occurs specifically in *Braf*-driven tumours (Extended Data Fig. 3f); the single WGD tumour with a *Hras* mutation also contains a *Braf* driver mutation (Fig. 2e). The significant co-occurrence between *Pyroxd2* and *Braf* (*P* = 0.016) mutations, along with mutual exclusivity of *Pyroxd2* with *Hras* (wMI *P* = 0.009), support the identification of *Pyroxd2* as a driver gene (Fig. 2f and Extended Data Fig. 3a).

Of note, our data revealed that genetic background can strongly shape the number of drivers per tumour. At least two driver mutations are typically identified per tumour in BL6, CAST and CAROLI, whereas C3H significantly differs with a median of only one driver (*P* = 1.97 × 10^−13^, Wilcoxon rank-sum test C3H versus other strains; Fig. 2g). This suggests that the higher liver tumour susceptibility of C3H may, in part, be attributable to fewer genetic changes being required for tumour inception or growth than in the other strains. By contrast, local effects such as a shared litter or the intra-animal environment had minimal, if any, impact on driver choice (Fig. 2h,i).

In summary, despite evolutionary divergence in key aspects of mutagenesis, genome stability and the number of genetic changes required for transformation, we found ubiquitous selection for activating mutations in MAPK pathway genes.

## Genetic background biases driver choice

**Fig. 3:**
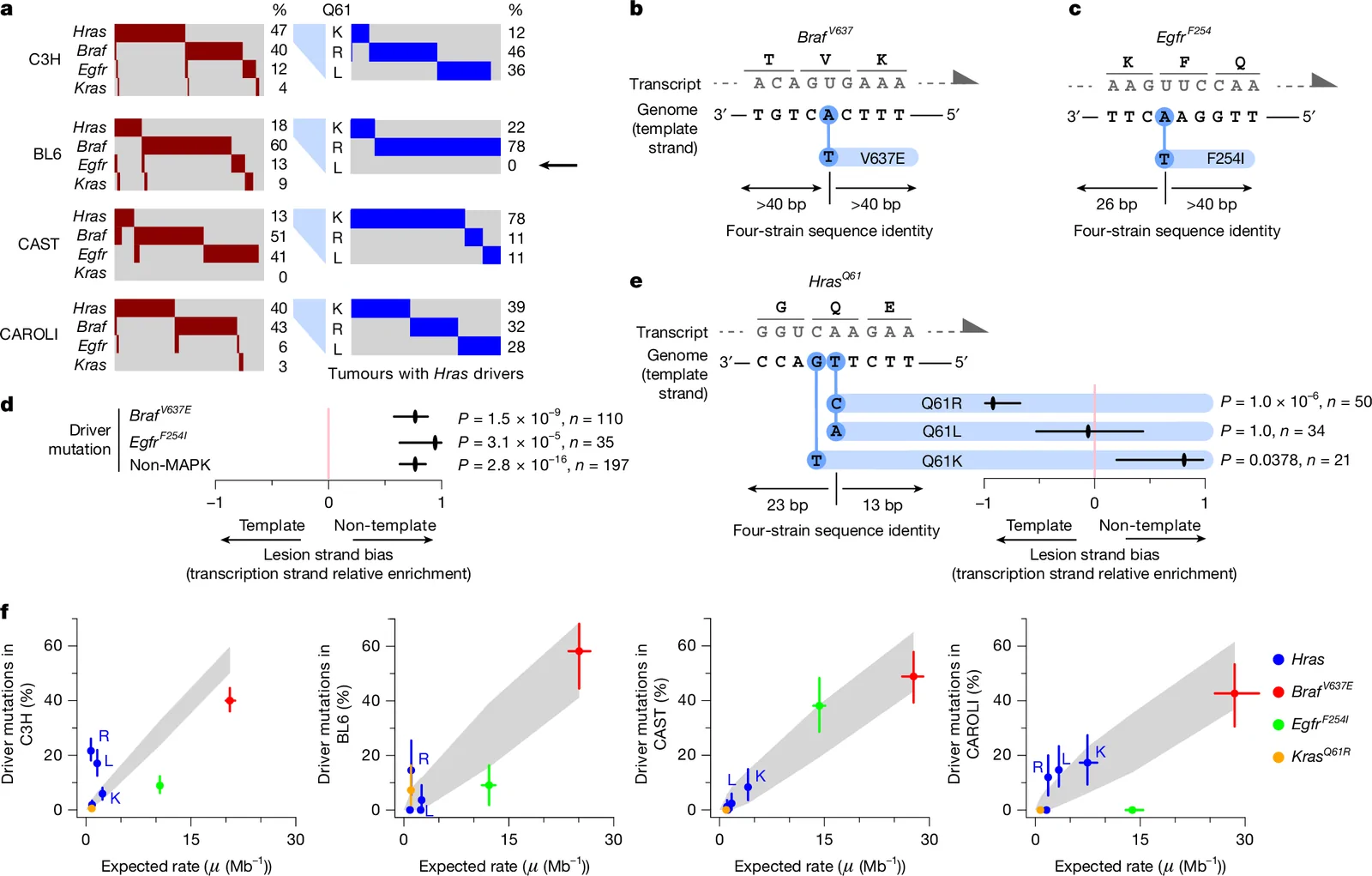
Genetic background influences the identity of MAPK pathway activating mutations. **a**, Most tumours harbour a MAPK pathway activating mutation, but the distribution across genes differs by strain (left panels). Among tumours with *Hras* mutations (right panels), strains show differences in the specific Q61 mutation, including complete absence of Q61L from BL6 (arrow). **b**, Schematic of the *Braf*^*V637E*^ mutation (orthologous to human *BRAF*^*V600E*^): an adenosine (A) on the genic template strand is mutated to thymine (T). The immediate sequence context is identical across each strain 40 or more nucleotides both upstream and downstream. **c**, Summary of the *Egfr*^*F254I*^ mutation (as panel **b**).** d**, Recurrent *Braf* and *Egfr* mutations show a strong preference for arising in tumours that inherited DNA damage (lesions) on the non-template strand (not subject to transcription-coupled repair) based on lesion strand bias (Methods; aggregate analysis over all four strains; whiskers show bootstrap 95% CI). Aggregate analysis over all non-MAPK pathway driver mutations shows the same bias for non-template strand damage. Lesion strand bias was assessed using two-sided Fisher’s exact tests; *P* values are Bonferroni corrected. **e**, Summary of *Hras* driver mutations (as panels **b**,**c**). *Hras*^*Q61K*^ mutations also show non-template strand bias. Of the mutations at *Hras* Q61 codon position 2, Q61R shows a strong bias for template strand damage and Q61L occurs in approximately equal numbers of tumours with template and non-template damage. **f**, Observed percentages of tumours with each recurrent MAPK mutation (*y* axis) versus expected mutation rate (*μ*; *x* axis). Expected rates were modelled from lesion-strand-resolved mutation rates and spectra, adjusting for transcription template strand and gene expression level (Methods). The whiskers show 95% bootstrap CI. Annotations (R, L, K; blue) identify specific *Hras* Q61 substitutions. The grey regions show the predicted 95% CI for driver mutations if their relative observed frequencies reflected only expected mutation rates. Source data

Despite almost universal acquisition of activating mutations in the MAPK pathway (Fig. 2e), the frequency with which each MAPK driver gene is mutated varies significantly between strains (*χ*^2^ *P* = 6.59 × 10^−14^; Fig. 3a). It is remarkable that mutations at codon 61 of *Hras* are found as prominent oncogenic drivers in all four strains (as in human cancers^39^), but the identity of the specific oncogenic amino acid change is highly divergent between genetic backgrounds (*χ*^2^ *P* = 2.69 × 10^−6^; Fig. 3a).

**Extended Data Fig. 4:**
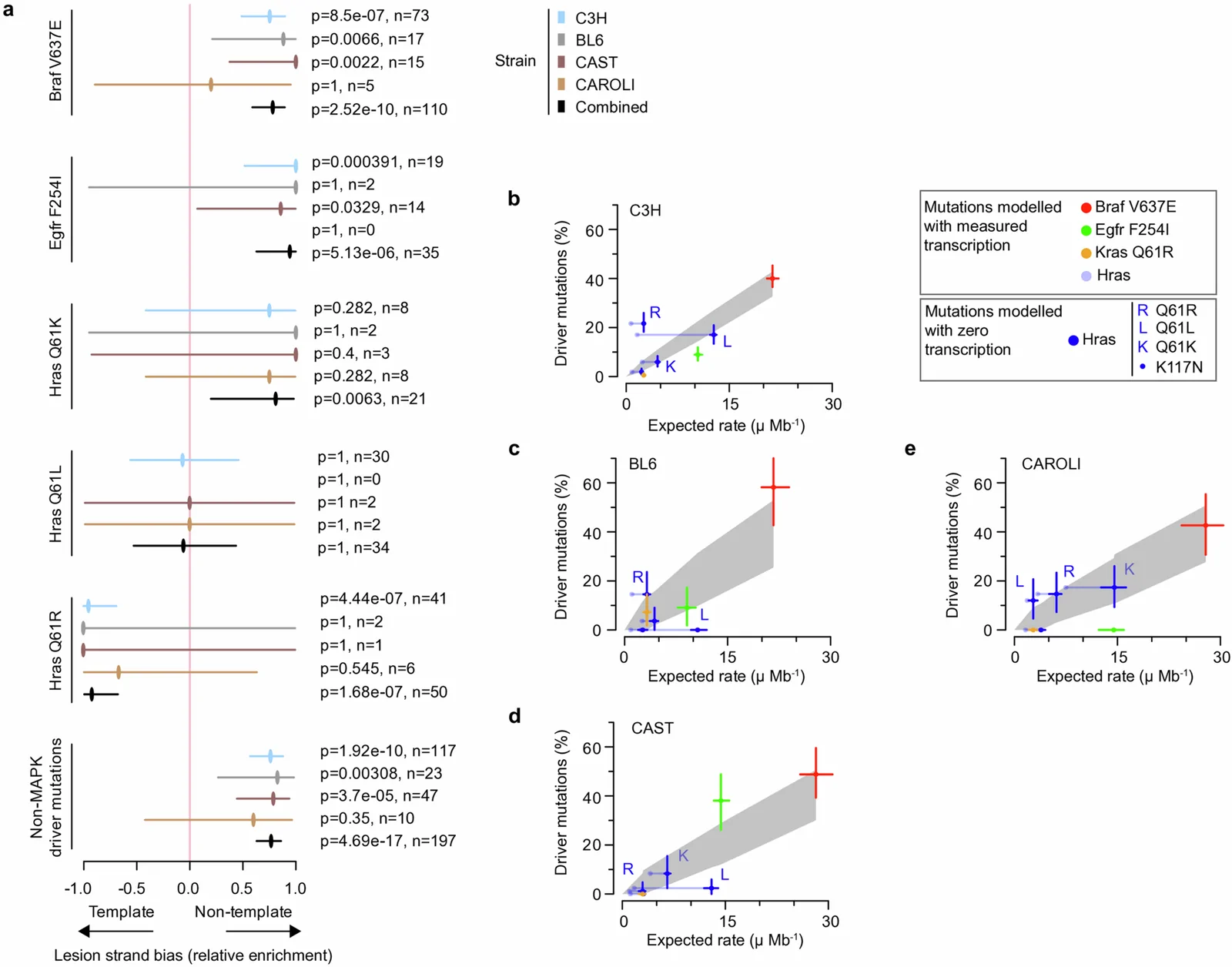
Strain and transcriptional influences on driver mutation bias. **a**, Bias of recurrent driver mutations to transcriptional template or non-template strand (x-axis) shown separately for each strain and the combined analysis aggregating across strains (black). Analysis only considers tumours that are mutationally asymmetric over the focal driver locus so that lesion strands can be confidently resolved. Whiskers show the bootstrap 95% confidence interval. The number of tumours with each mutation (n) and nominal p-value (p; two-sided Fisher’s exact test) indicated. Lower panel shows aggregated analysis for mutations in non-MAPK pathway genes identified as containing driver mutations. **b**, Observed percentage of C3H tumours with each recurrently observed MAPK activating mutation (y-axis) compared with the expected mutation rate (μ, x-axis). Expected mutation rates were calculated based on lesion-strand resolved mutation rate and spectrum, adjusting for transcription template strand and gene expression level (Methods). Confidence intervals (95%, whiskers) for both observed driver mutation percentage and expected mutation rate were calculated from bootstrap sampling of tumours. Expected mutation rates for *Hras* were calculated assuming zero transcription (dark blue); the expected rates calculated using observed P15 bulk liver RNA-seq estimates of *Hras* expression shown (light blue; also shown in Fig. 3f) and solid line (light blue) connecting the alternative estimates. **c-e**, Observed versus expected driver mutation rates for BL6, CAST, and CAROLI, respectively, shown as for **b**.

For every recurrent MAPK driver mutation, the extended sequence context is highly conserved, with no germline genetic differences within at least 13 nucleotides in any strain (Fig. 3b–e), thus excluding proximal sequence divergence as the explanation for different driver mutations. With the exception of *Hras*^*Q61R*^ and *Hras*^*Q61L*^ mutations, we found that all MAPK and other identified driver base substitutions are strongly biased to have been produced from DEN-induced damage on the non-template, rather than transcription template, strand (Fig. 3d,e and Extended Data Fig. 4a). Thus, mutated oncogenic proteins would not be produced until after the first round of replication following damage. By contrast, the *Hras*^*Q61R*^ mutations arise from template strand damage and therefore error-prone transcription over that damage could lead to the production of pro-oncogenic miscoded proteins before replication^40^.

We modelled the expected strain-specific rate of driver mutation occurrence (Fig. 3f). This modelling accounted for the heterogeneity of mutational burden and mutational spectrum between tumours, and captured the pronounced effects of transcription-coupled repair on mutational rate, using our strain and tissue-matched gene expression data (Fig. 1c). The rank order of expected MAPK mutations is closely matched between strains, and subtle differences in mutational spectra do not explain the marked phylogenetic divergence in driver utilization (Fig. 3f).

Both the *Hras*^*Q61R*^ and *Hras*^*Q61L*^ mutations are observed at higher frequencies than expected from modelling in several strains (Fig. 3f). Mutational spectrum suggests, and lesion strand bias confirms, that Q61R arises from T damage on the transcription template strand (Fig. 3e and Extended Data Fig. 4a). The low expected rates for *Hras*^*Q61R*^ and *Hras*^*Q61L*^ mutations reflect the high efficiency of transcription-coupled repair of DEN-damaged nucleotides^25,27^. Modelling shows that the observed driver mutation frequencies could be better explained if *Hras* is not transcribed in tumour progenitor cells during the time between DNA damage and the first round of DNA replication (Extended Data Fig. 4b–e). This would be consistent with transformation in a population of hepatocytes that are transiently quiescent at the *Hras* locus, which would be masked by bulk transcriptomic analysis.

**Extended Data Fig. 5:**
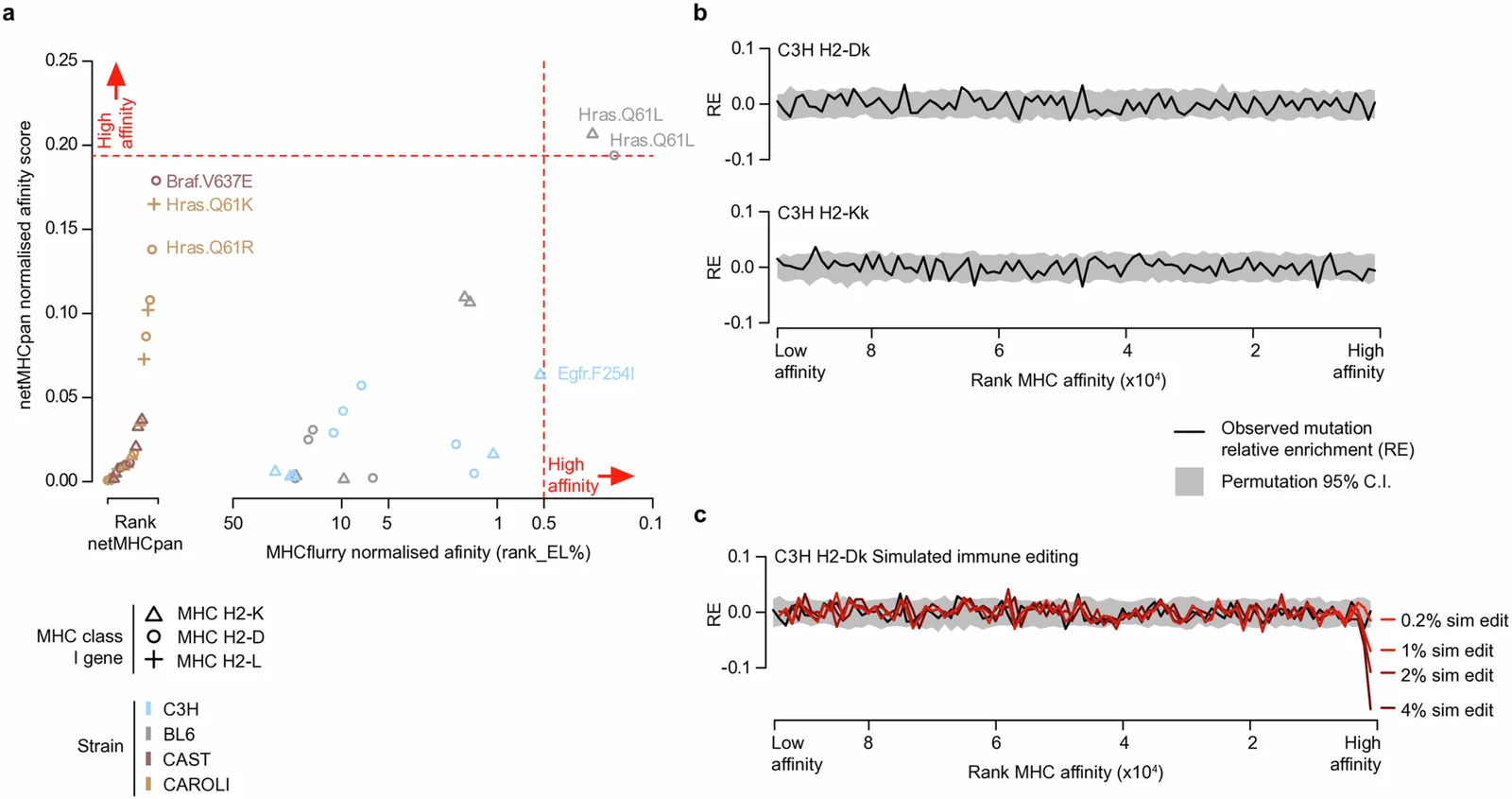
Immune presentation and editing of DEN-induced mutations. **a**, Predicted maximum affinity of recurrent driver mutation containing peptides for MHC class I molecules. Each of the mouse strains has two (C3H, BL6, CAST) or three (CAROLI) class I genes. For netMHCpan (y-axis) each mutation was scored for each MHC class I gene in each strain, whereas MHCflurry could only be applied to BL6 and C3H genes for which trained models exist. Scores for both netMHCpan and MHCflurry were quantile normalised as recommended and thresholds for high affinity binding indicated (right and above the red dashed lines). Only *Hras* Q61L in BL6 is predicted to be a high affinity binding peptide. **b**, For a defined MHC class I gene from an indicated strain (top, H2-Dk in C3H), all protein altering base substitution mutations from all four mouse strains were scored for their maximum binding affinity (MHCflurry). Binding scores were ranked and consecutive windows of 1,000 ranked scores were tested for the relative enrichment/depletion (RE) of strain-matched mutations compared to random expectation (C3H mutations when considering a C3H MHC gene). Immune editing of high affinity peptides is expected to show a depletion of strain-matched mutations (RE < 0) at high affinity, compared to non-strain-matched mutations. Confidence intervals (95%, grey area) were calculated from permuted affinity ranks. Example analyses from C3H shown, equivalent analysis for each MHC locus in each strain was performed but no compelling evidence of substantial immune editing was evident. **c**, Immune editing simulated by computational subtraction of high MHC affinity strain-matched mutations from the observed distribution of C3H MHC gene H2-Dk affinity scores, simulating from 0.2% (bright red) to 4% (dark red) of protein altering mutations being removed probabilistically dependent on affinity score. This indicates that in the bulk analysis of mutations we have power to detect ≥1% of protein altering mutations removed. However, BL6 *Hras* Q61L is predicted to be in the top 0.2% of affinity scores, which is not adequately powered in this dataset (bright red).

Despite *Hras*^*Q61L*^ and *Hras*^*Q61R*^ representing alternate changes of the same nucleotide that occur with similar frequency to each other in all other strains, Q61L is entirely absent from BL6 tumours (Fig. 3a). This suggests lineage-specific oncogenic selection. A potential source of such differences is immune editing from neoepitope presentation in the major histocompatibility (MHC) class I molecules. The *Hras*^*Q61L*^ mutation is predicted to have high affinity for MHC class I uniquely in BL6 (Extended Data Fig. 5a), potentially explaining the strain-specific absence of this mutation through epitope presentation and immune clearance. However, predictions of neoepitope presentation do not explain all strain differences in driver mutation frequency, and immune editing does not appear to be a major force shaping the observed distribution of mutations (Extended Data Fig. 5b,c).

## Epistatic disruption of gene expression

**Extended Data Fig. 6:**
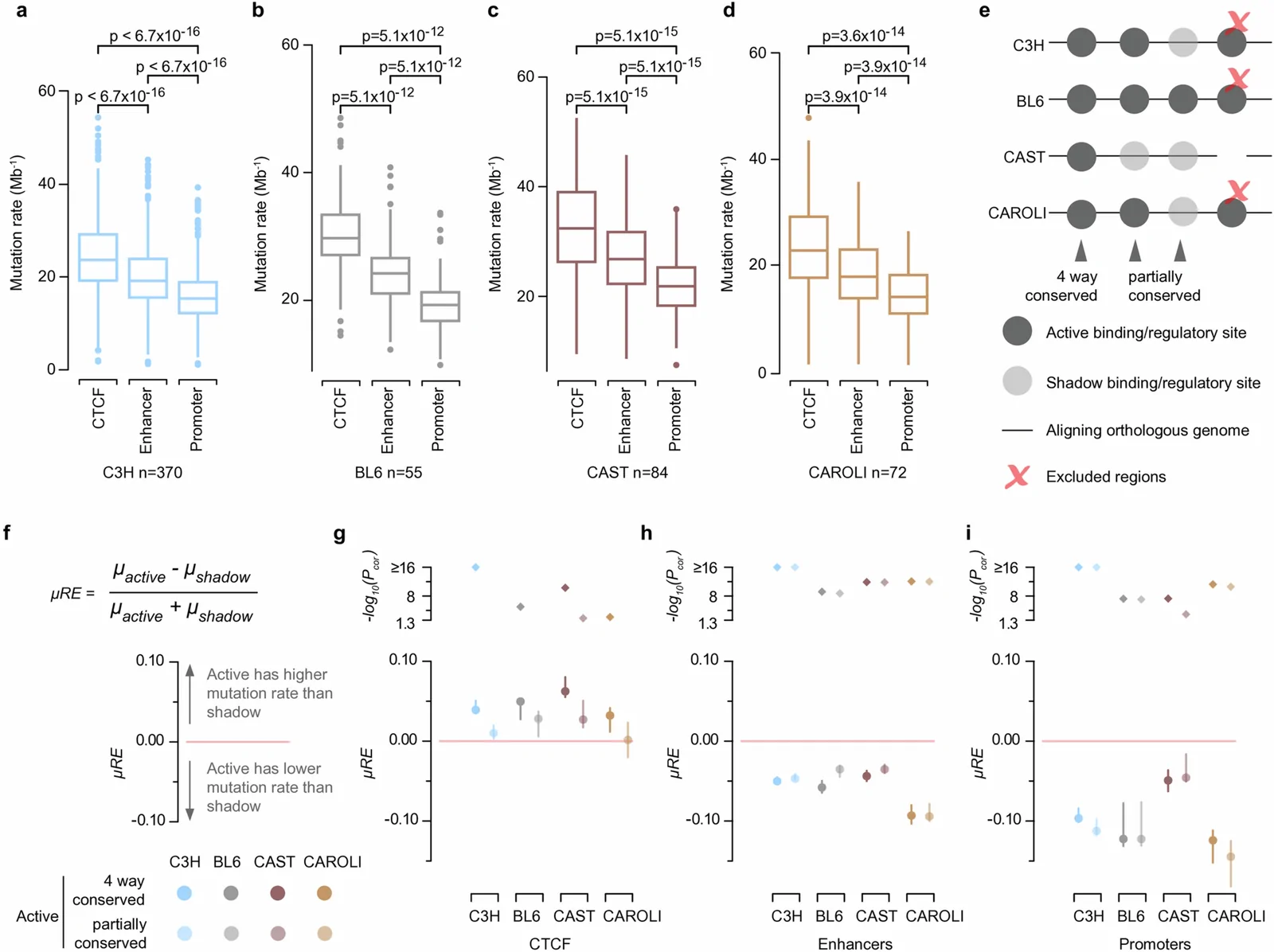
Mutation rates differ between regulatory site categories but show consistent behaviour between strains. **a**, Comparison of mutation rates between gene regulatory categories shows higher rates for CTCF binding sites than enhancers or promoters in C3H tumours. Mutation rates for ChIP-seq defined CTCF binding sites and histone modification defined active enhancers and promoters from P15 mouse liver. Rates calculated per tumour with outlier points indicated. The box defines the interquartile range and mid bar the median. Comparisons of mutation rate distribution between regulatory categories were calculated using a two-sided, paired Wilcoxon signed rank test and Bonferroni corrected for multiple testing. **b-d** Regulatory site mutation rate comparisons for BL6, CAST and CAROLI as for C3H in **a** shows a consistent rank of mutation rates with CTCF > enhancer > promoter. **e**, Using whole genome alignment, the cross strain conservation of regulatory activity was categorised. Orthologous regulatory sites active in all four strains were classed as 4 way conserved. Partially conserved sites were those where orthologous sequence could be identified in each strain but binding/histone modification was only identified in a subset of the four strains; the corresponding unbound regions are defined as shadow sites. Sites where orthologous sequence could not be identified in all strains were excluded from subsequent regulatory site analysis. **f**, The relative enrichment (RE) of mutation rates (μ) was calculated between active regulatory sites and shadow sites as defined in **e**. **g**, Active CTCF binding sites have higher mutation rates than shadow CTCF binding sites (μRE > 0), and 4 way conserved CTCF sites have higher mutation rates than partially conserved CTCF sites. Statistical significance (top panel) was calculated using two-sided, paired Wilcoxon signed rank test, between mutation rates for active and shadow binding sites, plotted p-values (y-axis) are Bonferroni corrected. Whiskers show bootstrap 95% confidence intervals. **h**, Active enhancers have lower mutation rates than shadow enhancers; calculated and plotted as in **g**. **i**, Active promoters have lower mutation rates than shadow promoters; calculated and plotted as in **g**.

We next examined the downstream consequences of DEN-induced mutagenesis for gene regulation and the resulting tumour phenotypes. We profiled the transcriptome of whole-genome-sequenced tumours (*n* = 386) using total RNA-seq and characterized the regulatory landscape of strain-matched normal livers (P15) using ChIP–seq, ATAC-seq and RNA-seq (Fig. 1c). Extending previous findings^25,27,28,41^, we showed that regulatory activity influences DNA damage and repair and that these features are conserved across strains (Extended Data Fig. 6 and Supplementary Note).

**Fig. 4:**
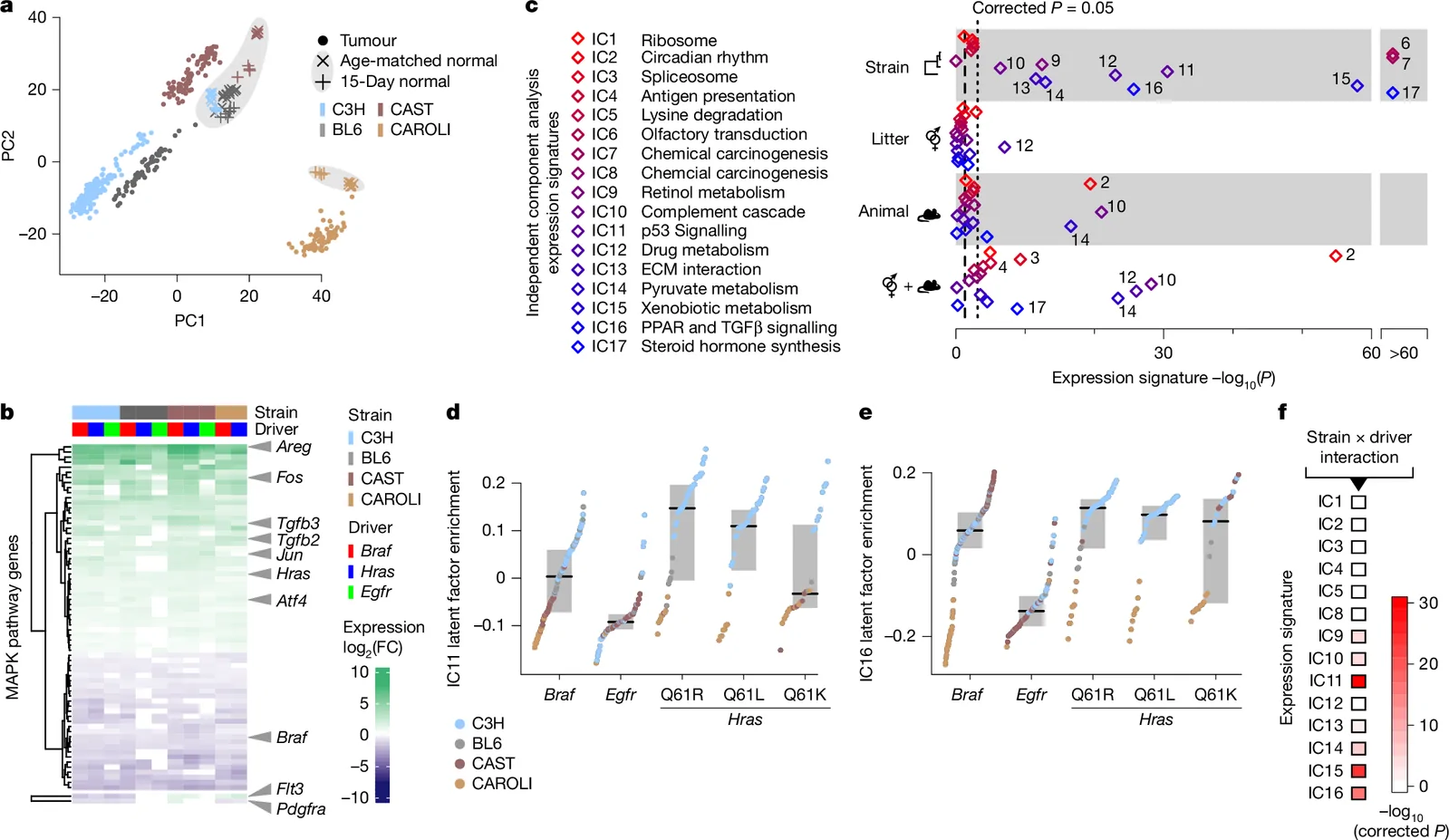
Tumours from different strains converge on a common transcriptional state. **a**, For expression analysis of DEN-induced tumours and untreated liver tissue, the first two principal components (PC1 and PC2) capture expression clustering by strain (colour), and developmental stage and tumour status (shapes). **b**, MAPK pathway genes are consistently differentially expressed between age-matched normal liver and *Braf*-driven, *Hras*-driven and *Egfr*-driven DEN-induced tumours. The only MAPK genes with discordant, significant expression changes between strains are *Flt3* and *Pdgfra*. FC, fold change. **c**, IC analysis of tumour gene expression identifies 17 transcriptional signatures that vary between DEN-induced tumours (Extended Data Fig. 8a). Eleven of these signatures are significantly associated with strain (likelihood ratio tests of nested mixed-effects models; to the right of the dotted line are *P* < 0.05 after Bonferroni correction). The dashed line denotes *P* = 0.05, and the dotted line indicates the Bonferroni-corrected *P* = 0.05. ECM, extracellular matrix. Several signatures show significant coordination across litter, animal and combined litter + animal interaction, most notably signature IC2, which is enriched for circadian rhythm genes. **d**, Median (black line) enrichment of p53 signalling-associated IC11 gene expression signature (*y* axis) stratified by MAPK driver mutation (*x* axis) and strain (colour). **e**, Enrichment of PPAR and TGFβ signalling-associated IC16 expression signature, plotted as in panel **d**. **f**, Significance of strain × driver mutation interaction (Bonferroni-corrected *P* values) for each expression signature on the basis of the likelihood ratio test of nested linear mixed-effects models that differ only by strain × driver interaction. Source data

**Extended Data Fig. 7:**
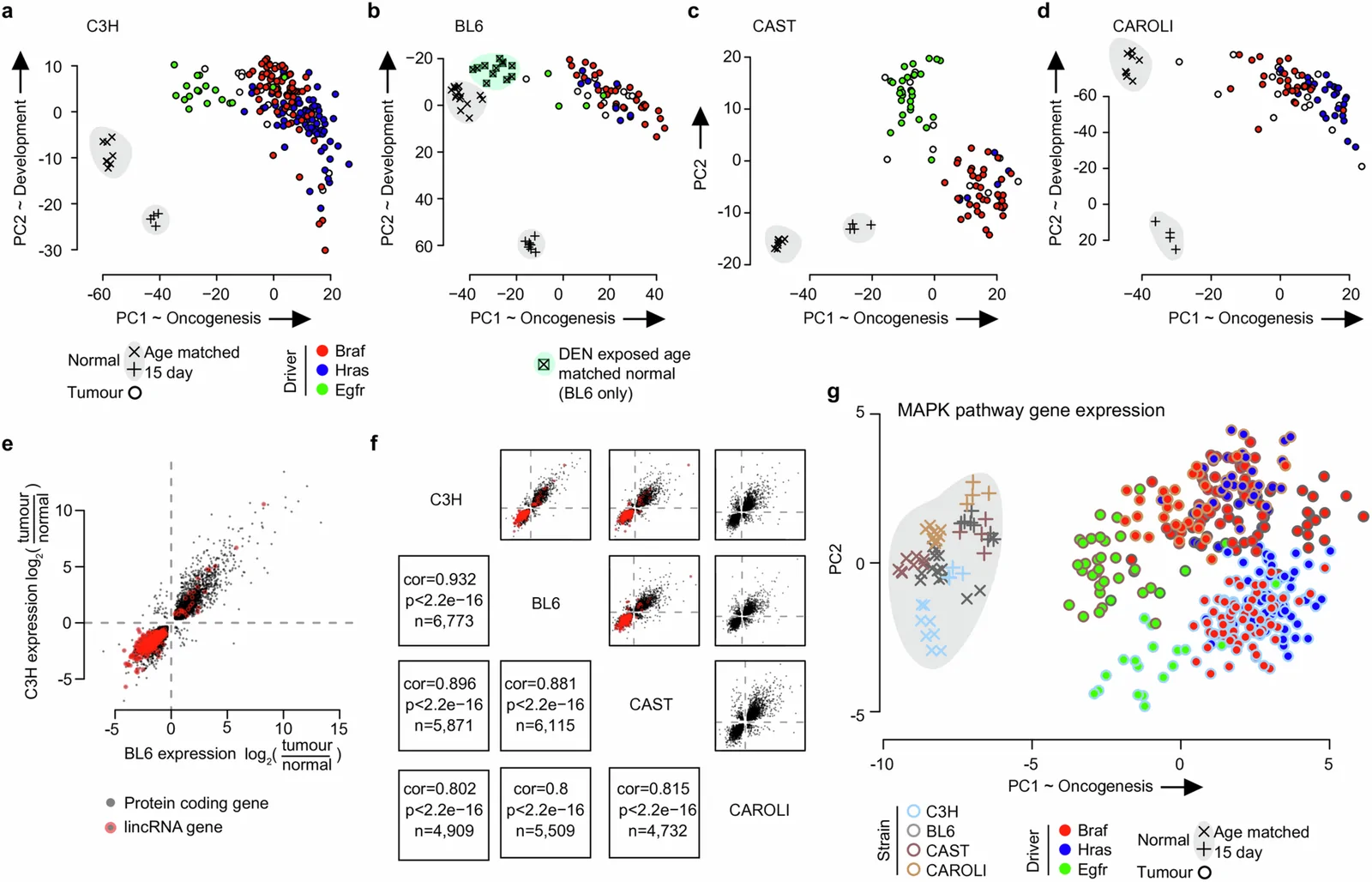
Oncogenic gene expression changes. **a**, Principal component expression analysis of C3H tumours, along with comparison to age-matched liver tissue from DEN-naive animals (grey X) and DEN-naive animals at P15 (grey +), matching the time of DEN administration. This demonstrates an axis of oncogenic transformation (PC1) that is distinct from ageing or developmental changes in gene expression (PC2). **b**, Principal component expression analysis for BL6 samples, as in panel **a** but additionally including comparison to background liver sampled distant to tumours in the DEN-exposed animals (green checked square). **c**, CAST expression analysis as in panel **a**. While PC1 represents oncogenesis, specifically in this strain PC2 captures expression differences between driver mutations rather than an axis of development. **d**, CAROLI expression analysis as in panel **a**. **e**, Changes in gene expression between age matched normal liver and liver tumours are highly correlated between C3H and BL6. Each point denotes the expression change (*log*_*2*_*(tumour/normal)*) of a single orthologous gene pair between the BL6 and C3H genomes, only genes significantly differentially expressed in both strains are shown. In contrast to protein coding genes (grey), lincRNAs (red) are strongly biassed to reduced expression in tumours compared to normal liver. **f**, All versus all plots between strains showing the correlation of gene expression changes between age-matched normal liver and tumour, as per panel **e**. The C3H versus BL6 sub-panel is the same data as panel **e**. Pearson’s correlation coefficients, corresponding p-values, and counts of differentially expressed genes are shown. Equivalent lincRNA annotation is not available for CAROLI. **g**, Principal component analysis of MAPK pathway gene expression demonstrates the axis of oncogenic transformation is consistent between strains (PC1) and highlights the muted transcriptional perturbation of tumours with *Egfr* driver mutations compared to *Braf* and *Hras* (also seen in Fig. 4b).

Transcriptome analysis demonstrated strain differences in gene expression for both P15 livers and DEN-induced tumours, with CAROLI being the most divergent phylogenetic outgroup (Fig. 4a). Within each strain, the majority of variance in expression between samples captures the oncogenic progression from normal age-matched liver to DEN-induced tumour (Extended Data Fig. 7a–d), and long non-coding RNAs are consistently downregulated in the tumours (Extended Data Fig. 7e,f). Across strains, driver mutations in MAPK pathway genes are the principal drivers of DEN-induced tumours (Fig. 2e), and many MAPK-regulated genes are differentially expressed between age-matched normal and DEN-induced tumour tissue (Fig. 4b). This profile of MAPK transcriptional perturbation is remarkably consistent across strains and generally between driver mutations, although the magnitude of change is slightly reduced in *Egfr*-driven tumours (Fig. 4b and Extended Data Fig. 7g).

**Extended Data Fig. 8:**
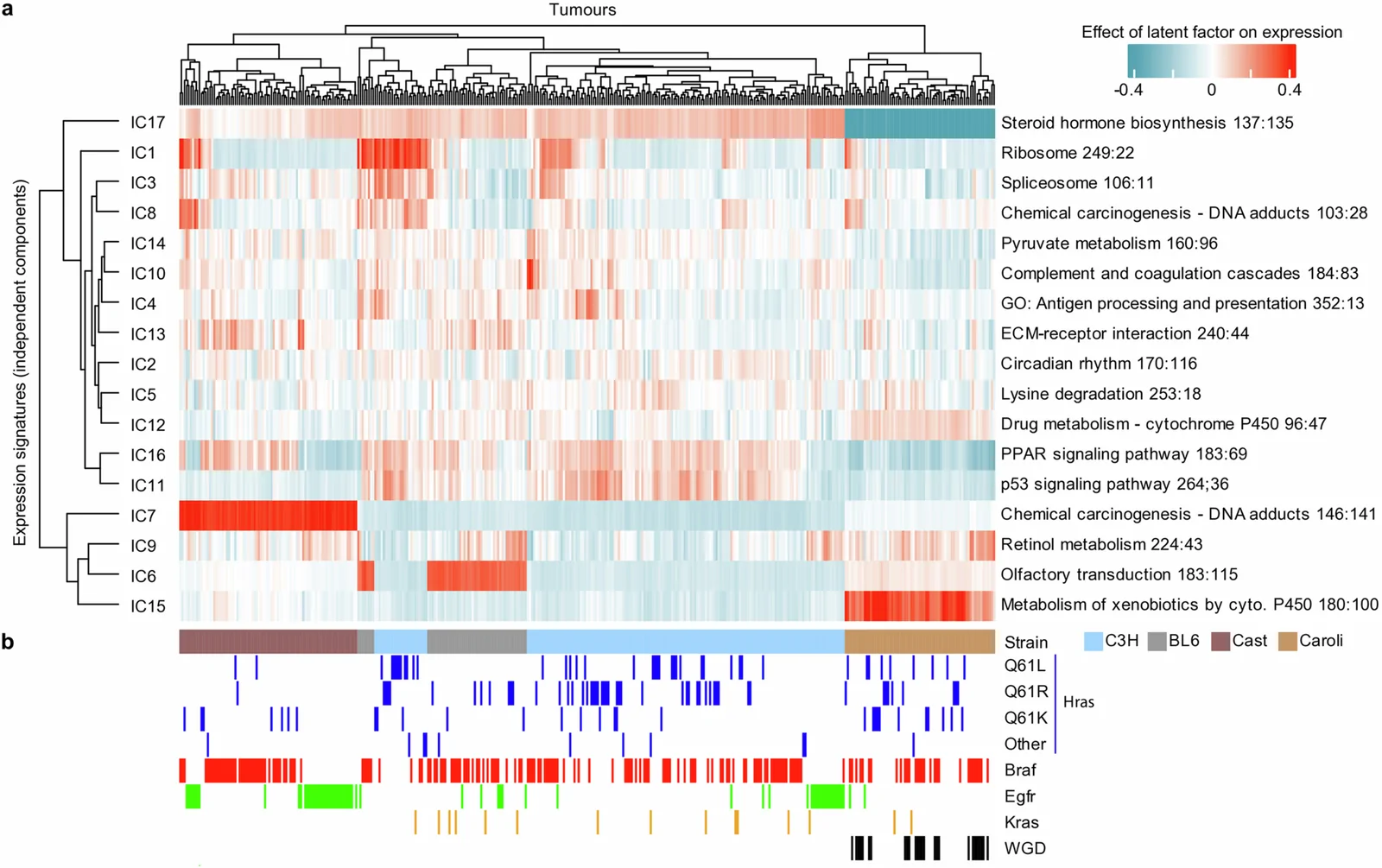
Deconvolving signatures of tumour gene expression. **a**, Independent component analysis deconvolves the expression of DEN-induced liver tumours (x-axis, hierarchical clustering tree above) into 17 distinct expression signatures (IC1 to IC17). The contribution of each signature to each tumour is indicated by the heatmap colours (orange=enriched, blue=depleted). Annotation to the right of the heatmap rows shows the Kyoto Encyclopedia of Genes and Genomes (KEGG) or Gene Ontology (GO) annotation terms most over-represented amongst the genes defining the signature. Numbers shown as a ratio A:B denote the number of genes whose expression increases the signature (A) versus the number whose expression decreases the signature (B). **b**, The strain and driver mutations for each tumour are indicated below the heatmap.

We next specifically considered gene expression differences among DEN-induced tumours. Independent component (IC) analysis identified 17 signatures (IC1–IC17) of gene expression, each of which represents a defined set of genes that are correlated in their expression (Extended Data Fig. 8a and Supplementary Table 5).

**Extended Data Fig. 9:**
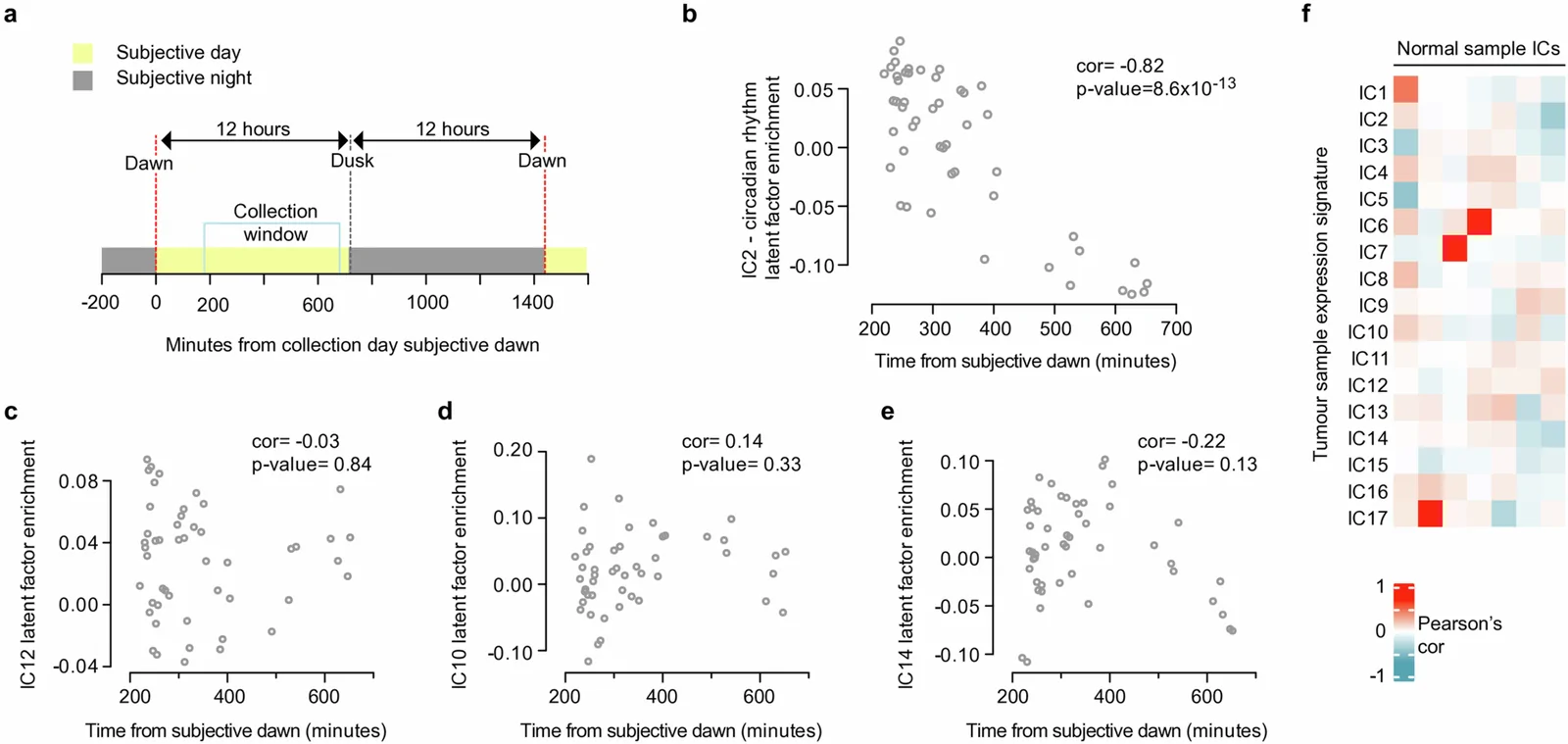
Explaining expression signatures that differ between tumours. **a**, Schematic showing the tumour collection window relative to the 12 h light/dark cycle in the vivarium, with subjective dawn at circadian time 0. **b**, For BL6 mice there are accurate records of tumour collection time (x-axis), which strongly correlate with the IC2 expression signature (y-axis), that is enriched for circadian rhythm genes. **c-e**, Expression signatures (y-axes) IC12 (drug metabolism), IC10 (complement cascade), and IC14 (pyruvate metabolism) also show animal or animal+litter associations (Fig. 4c) but these do not significantly correlate with tumour collection time, suggesting individual differences in immune or metabolic processes that are independent of circadian cycles. **f**, Heatmap showing Pearson’s correlation between the 17 tumour derived IC signatures and IC signatures derived from normal samples. Tumour IC6, IC7, and IC17 are highly correlated with normal sample ICs and perfectly discriminate strains; they are therefore excluded from subsequent analyses.

In contrast to genome stability and driver mutation choice, several gene expression signatures show significant associations across tumours from the same animal or litter (Fig. 4c), suggesting that elements of shared environment contribute to tumour transcription. Preeminent among these is signature IC2, which is enriched for circadian rhythm genes. In our experimental system, the time of DEN administration was consistent, but we found that the timing of tumour collection was highly correlated with IC2 (Extended Data Fig. 9a,b). This demonstrates that, despite the substantial transcriptional rewiring between normal liver and tumour, clonally distinct tumours maintain the circadian coordination of transcription. Signatures IC10, IC12 and IC14 also show similar animal and animal + litter associations to IC2 (Fig. 4c), but are not correlated with tumour collection time (Extended Data Fig. 9c–e), indicating other environmental coordinators of tumour gene expression.

Three expression signatures (IC6, IC7 and IC17) perfectly discriminate strains and are similarly found in non-tumour liver tissue (Extended Data Figs. 8a,b and 9f); these baseline differences are excluded from subsequent enrichment analyses. A further eight signatures show strong associations with strain (Fig. 4c), two of which are enriched for cancer-associated pathways: IC11 enriched for p53 cellular stress response signalling, and IC16 enriched for peroxisome proliferator-activated receptor (PPAR) and TGFβ signalling. Both IC11 and IC16 signatures exhibit pronounced non-additive interactions between strain and specific driver mutations (Fig. 4d–f). For example, CAST tumours with *Braf* mutations are more positively enriched for IC16 than C3H tumours with the same driver mutation, whereas *Egfr*-driven tumours show the exact opposite. Using mixed-effects models, we systematically tested for such epistatic interactions between driver mutations and genetic background, finding highly significant interactions for IC11, IC16 and IC15 (Fig. 4f). Although transcriptional disruption to the core MAPK pathway appears largely consistent between driver mutations and strain, pronounced epistatic interactions between driver mutation and genetic background on the cellular stress response (p53, IC11) and mitogenic signalling (PPAR and TGFβ, IC16) provide a plausible mechanistic basis for strain differences in driver mutation choice and tumour latency.

## Genetic background shapes selection

**Fig. 5:**
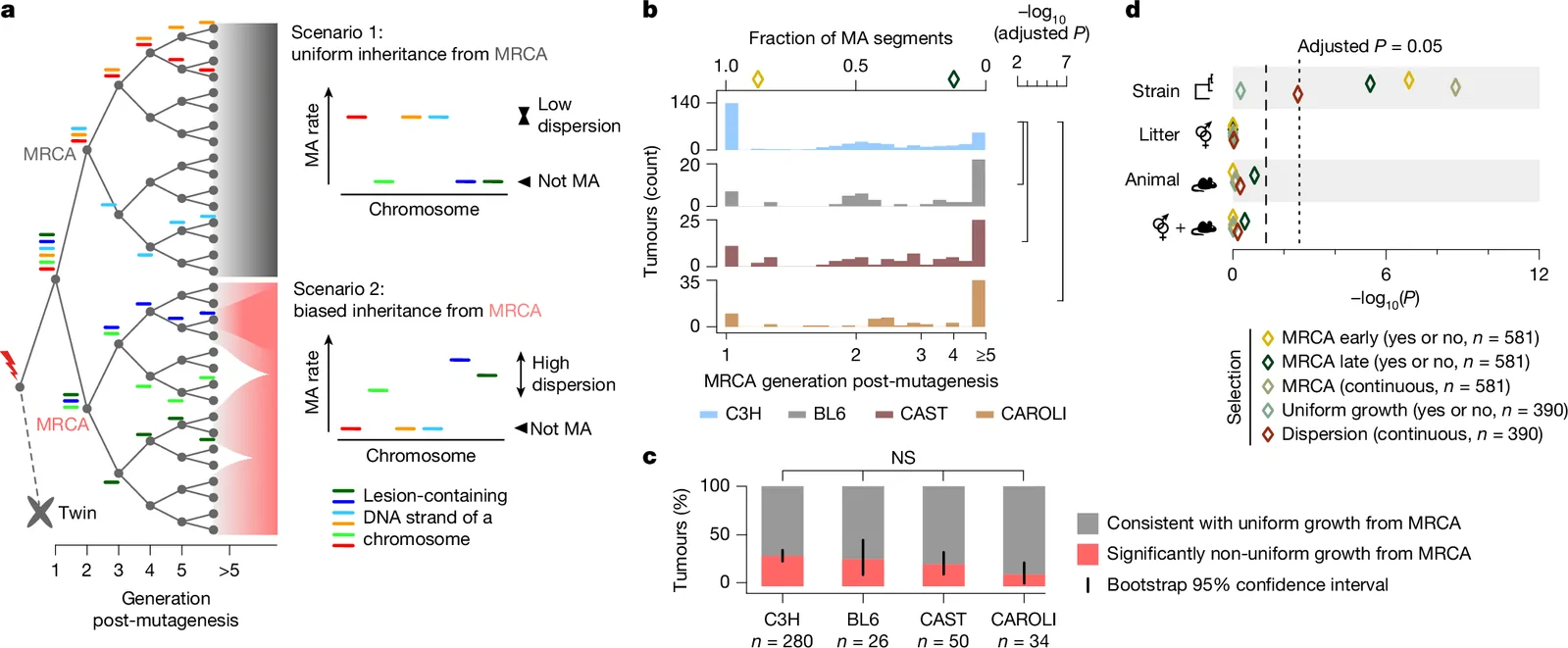
Genetic background affects tumour clonal dynamics. **a**, Schematic of the segregation of lesion-containing chromosome strands (coloured bars) through cell generations (*x* axis) following mutagenesis (red bolt). Typically, only one daughter of a mutagenized cell contributes to a tumour, so the twin lineage is lost (dashed line). Scenario 1 (top panel, grey) shows a tumour that develops from a MRCA in the second generation, with all subsequent lineages contributing equally. Chromosomes without a lesion strand in the MRCA are not multiallelic (MA); chromosomes with a lesion strand in the MRCA all have approximately the same MA rate in the tumour. Scenario 2 (bottom panel, pink) also shows the evolution of a tumour with a second generation MRCA, but unequal contributions by subsequent lineages causes heterogeneity of the MA rate (dispersion) between chromosomes that inherit a lesion-containing strand. **b**, The fraction of chromosomal segments with MA variation (*x* axis) identifies the cell generation post-mutagenesis of the MRCA of each tumour. The frequency distribution (*y* axis, tumour count) of MRCA generation is different between C3H and the other strains (two-sided Kolmogorov–Smirnov test *P* values, Bonferroni corrected, shown for pairwise comparisons *P* < 0.05), with the majority of C3H tumours having their MRCA in the first cell generation post-mutagenesis. **c**, The percentage of tumours that reject a model of uniform post-MRCA growth (*y* axis, pink) is not significantly different between strains (*x* axis). The black bars show bootstrap 95% confidence intervals. **d**, Likelihood ratio tests of nested linear mixed-effects models show significant strain associations for dichotomized early:not early MRCA and late:not late MRCA (see top *x* axis of panel **b** for thresholds) and MRCA as a continuous measure (fraction of MA segments). No animal, litter or combined associations with clonal growth measures are significant (the dashed line denotes nominal *P* = 0.05, and the dotted line indicates Bonferroni-corrected *P* = 0.05). Source data

Finally, we considered the clonal dynamics of tumour development. The persistence of DNA damage through multiple rounds of replication can generate multiallelic variation—that is, multiple different mutations at the same site—within an expanding clone^25,26,30^. This multiallelic variation represents the generation of extensive genetic diversity within the first cell divisions following a burst of mutagenesis, affecting both the presence or the absence of subclonal mutations and their co-occurrence within a sublineage. The segmental pattern of multiallelic variation across the tumour genome can identify which cell generation post-mutagenesis gave rise to the most recent common ancestor (MRCA) of that tumour^25^. If the MRCA of a tumour is a first-generation daughter of a mutagenized cell, then one strand of every chromosome retains DNA damage and results in genome-wide multiallelic variation. Damage-containing strands randomly segregate through subsequent cellular generations, losing on average 50% of damaged strands per generation. Consequently, a tumour developing from a second-generation MRCA is expected to have only 50% of the genome as multiallelic, 25% in a third-generation MRCA and 12.5% in a fourth-generation MRCA (Fig. 5a).

**Extended Data Fig. 10:**
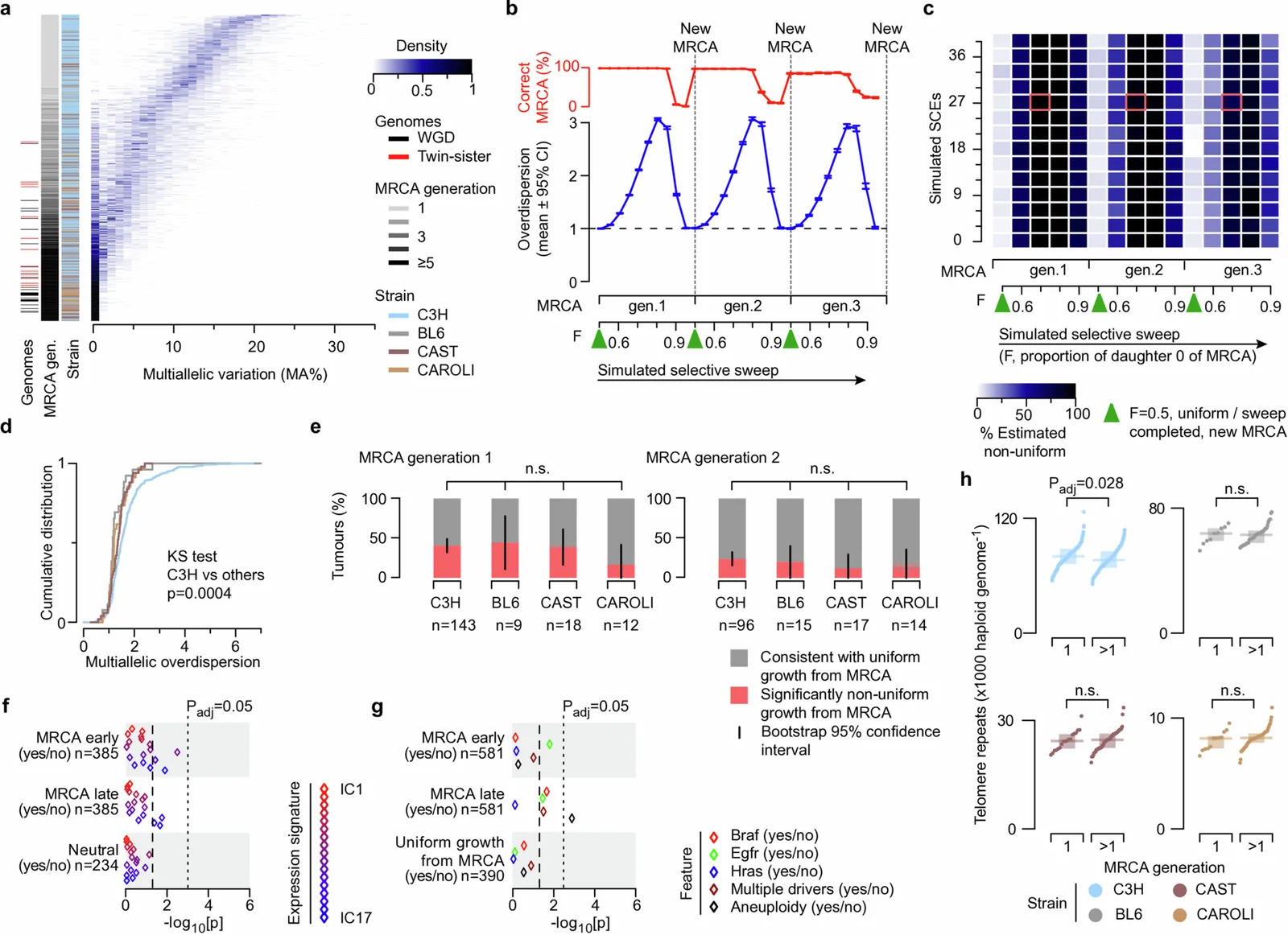
Subclonal dynamics of tumour development. **a**, Heatmap summary of multiallelic (MA) rate distribution for genomic segments in each tumour (rows), ranked by mean MA rate plotted in consecutive 1% bins (x-axis). Annotation of MRCA generation post-mutagenesis is based on the fraction of MA segments. Whole genome duplicated (WGD, black) and cases where both daughters of the originally mutagenised cell contributed to the sequenced tumour (twin-sister, red) are indicated. **b**, Computationally simulated DEN-induced tumours with a defined generation of MRCA (x-axis) and subclonal selection (F, the proportion of the final tumour contributed by the clonally dominant daughter cell of the MRCA). Green triangles (F = 0.5) denote neutral evolution from the MRCA, and increasing F reports greater subclonal imbalance. The overdispersion metric (blue; Methods) was calculated for each selection scenario, whiskers denote 95% confidence. At extreme levels of F (> 0.8) with 30x simulated read coverage, multiallelic variation starts to drop below detection sensitivity and strong subclonal sweeps are classified as a next generation MRCA (red). **c**, Power analysis for the detection of non-uniform post MRCA growth using the truncated binomial model (see Methods). The MRCA generation and extent of simulated subclonal sweep (x-axis as in **b**) plotted against simulation scenarios spanning a range of SCE events per genome (y-axis). Heatmap shading denotes the percent of time the corresponding simulation rejects neutral evolution at p < 0.05. Red border denotes simulated F = 0.7 with 27 generation 0 and 27 generation 1 sister chromatid exchanges for which there is 100%, 91%, and 71% power to detect non-neutral evolution in MRCA generations 1, 2, and 3 respectively. **d**, Compared to the other strains, C3H (blue, key shared with panel **a)** has a greater proportion of tumours (cumulative distribution, y-axis) with a high multiallelic coefficient of dispersion (x-axis), indicating unequal contributions of sub-clones to the sequenced tumour cell population. **e**, Detection of non-uniform post-MRCA growth in DEN-induced tumours, stratified by MRCA generation. Statistical testing by all-versus all two-sided Fisher’s exact tests with Bonferroni correction, all tests non-significant even before correction. Bootstrap 95% confidence intervals shown for the percent with non-neutral evolution. **f**, Testing gene expression signature latent variable enrichment as response variables for association with binarised measures of selection (see Methods). P-values (x-axis) from likelihood ratio tests of nested linear mixed-effects models, Bonferroni corrected threshold for p < 0.05 significance indicated (dotted line). **g**, Testing binarised measures of selection as response variables for association with candidate driver mutations (presence or absence), and associated features. P-values (x-axis) from likelihood ratio tests of nested linear mixed-effects models, Bonferroni corrected threshold for p < 0.05 significance indicated (dotted line). MRCA late tumours (<10% of genomic segments with multiallelic variation) are significantly associated with the presence of aneuploidy events which may provide a second oncogenic hit. **h**, Specifically in the C3H strain, telomeres (y-axis) are significantly longer in first generation MRCA than in later generation tumours. Statistical testing by two-sided Mann-Whitney test with Bonferroni correction.

Quantification of multiallelic rates across the tumour genomes demonstrates that all strains are able to instantly transform; that is, where a first-generation post-mutagenesis cell is the MRCA of a tumour (Fig. 5b and Extended Data Fig. 10a). This is remarkable, because the first-generation MRCA tumours have essentially retained all the multiallelic and combinatorial genetic diversity generated from the initial burst of DNA damage and exhibit minimal loss of sub-lineages. Instant transformation is consistent with aspects of the big bang model of transformation^42^, but contrasts with a prevalent view of cancers developing through strong selection and recurrent subclonal sweeps^43^.

Of note, C3H tumours have a high propensity for a first-generation MRCA, whereas the other strains typically have later-generation MRCAs (two-sided Kolmogorov–Smirnov test, C3H versus the aggregate of other strains, *P* = 6.6 × 10^−9^; Fig. 5b). Later-generation MRCAs could arise from either (1) increased early lineage loss, in which only a subset of early post-mutagenesis cells survive or grow to form tumours; or (2) strong late selective sweeps (an early clone expands but most cells are subsequently removed, resulting in apparent late MRCA). To differentiate these scenarios, we examined the heterogeneity of multiallelism between chromosomal segments within each tumour. If all subclones arising after the MRCA contribute similarly to the tumour, then lesion-containing segments should show similar multiallelic rates (Fig. 5a, scenario 1). By contrast, if some subclones expand at the expense of others, the biased inheritance will increase the variability (dispersion) of multiallelic rates across the genome (Fig. 5a, scenario 2, and Extended Data Fig. 10a–c).

We quantified this dispersion (Extended Data Fig. 10b,c) and did not find that C3H exhibited the most uniform growth from the MRCA (Extended Data Fig. 10d), arguing against strong late selective sweeps in the other strains. Moreover, the similar percentage (23 ± 8%) of tumours exhibiting significantly non-uniform growth (Fig. 5c and Extended Data Fig. 10e) suggests that all strains show comparable uniformity of post-MRCA clonal growth. Instead, our results implicate differences in lineage loss, specifically during the earliest stages of tumour initiation, as being responsible for the strain biases in clonal dynamics.

Concordant with most other aspects of the tumour evolution, MRCA generation is strongly associated with strain, but not with shared litter or animal (Fig. 5d). Across strains, the gene expression signatures, driver events and other tumour features were not robustly associated with tumour clonal dynamics, the exception being an enrichment of aneuploidy in late MRCA generation tumours (Extended Data Fig. 10f,g). Uniquely in C3H (the strain with frequent generation-one MRCAs and the longest telomeres), we found that first-generation MRCA tumours had longer telomeres than later MRCA tumours (two-sided Mann–Whitney test with Bonferroni correction, *P* = 0.028; Extended Data Fig. 10h), which may confer a growth advantage.

## Discussion

Despite the availability of millions of human cancer genomes and sustained interest in genome–environment interactions^9^, how the genetic background of an individual shapes tumour development remains incompletely understood^2^. Retrospective cohort studies are constrained by substantial variation in diet, sex, environment, mutagen exposure and sociodemographic history. Conversely, tumours arising in people with cancer predisposition syndromes^16,17^, or genetically engineered animal models^44,45^, are overwhelmingly driven by high-penetrance single-gene effects. These strong genetic drivers can mask more subtle polygenic or environmental contributions^18,19,46^. Similarly, although systematic single-locus screens have identified novel host regulators^47^, this approach cannot consider epistatic interactions with wider genetic variation.

To complement these approaches, we introduced a prospective strategy that leverages the evolutionarily acquired genetic diversity of mouse genomes, a well-established model of tumorigenesis^21,24,25^, and the hierarchical relationships of shared animal and litter environments. Using this framework, we show that nearly every measure of mutation and selection was strongly correlated with genetic background, underscoring its importance in constraining and directing tumour evolution. We illustrated the mechanistic basis of these genetic background differences, which might also shape otherwise-enigmatic features of human cancer development. These include population-specific driver variation, the occurrence and impact of early WGD, and differences in selection driving tumour latency and susceptibility.

It was plausible that coordinated metabolic or immune activity within a given animal or litter would influence cancer initiation, progression or clearance. However, in contrast to the pervasive genetic background effects on tumour development, we found no evidence of correlated mutagenesis, genome instability or selection among tumours arising within the same animal or litter. This is despite considerable statistical power from the study design and identification of animal and litter-coordinated effects on circadian rhythm and telomere length. We conclude that genetic background, rather than individual-level factors, predominantly defines the trajectories available for tumour development.

Rerunning tumour evolution converged on a common pathway of vulnerability for oncogenesis that is shared among humans^48^ and the genetically divergent strains of mice in our study. The acquisition of DEN-induced activating mutations in the MAPK pathway genes *Braf*, *Hras*, *Egfr* and *Kras* was an almost universal feature of the tumours, and similar perturbation of the core MAPK transcriptional programme was common to all strains. Each genetic background exhibited pronounced preferences not only for which MAPK gene was mutated but also for specific amino acid changes within each driver gene. These population-specific patterns of driver utilization could not be attributed to strain-specific mutational spectra or local DNA sequence context. Strain-specific immune surveillance might explain the absence of *Hras*^*Q61L*^ from BL6, but this mechanism does not generalize to the broader set of driver differences that we observed.

Our results establish novel mechanistic insights into how population-specific biases in driver utilization can occur: epistatic interactions between cancer driver mutations and genetic background. We identified strain specificity in how MAPK driver mutations perturb the transcriptional regulation of key cancer signalling pathways (p53, TGFβ and PPAR), while consistently perturbing the core MAPK pathway. Disruption of these critical pathways is predicted to alter cell division, differentiation, inflammation and apoptosis^49–51^, potentially resulting in a selective advantage, but only when the driver mutation arises on the synergistic genetic background. This concept of somatic–germline epistasis is not currently considered in the design and validation of clinical prognostic and predictive tests. Our results indicate that this may be an important consideration because driver mutations do vary in frequency between human populations^2,15^. The same genetic change in populations of distinct ancestry may not only reflect differences in mutagenesis^4^ but could also confer vastly different oncogenic consequences and clinical outcomes.

Epistatic interactions between driver mutation and genetic background may also underlie WGD, which in humans is enriched in cancers from specific ethnic groups^52^. Similarly, among mouse strains, only CAROLI exhibits WGD and then only in *Braf*-driven tumours. The strain-specific acquisition of WGD subsequently introduced genome instability, leading to secondary aneuploidies in these CAROLI tumours. This parallels mechanisms and evolutionary trajectories that are prevalent in human cancers^53^, in which WGD is a critical and often early^53^ event in tumour evolution that confers poor prognosis^54^.

Genetic background also profoundly influences tumour latency and cancer susceptibility. By exploiting multiallelism, we showed that strains differ in the number and nature of early driver events required for transformation, revealing germline-dependent variation in selection and subclonal survival at the earliest stages of tumour development. For instance, C3H, the most cancer-susceptible strain, frequently transforms with a single driver mutation and minimal loss of subclone lineages, whereas other strains typically require two or more driver events and show extensive loss of early subclones. These differences in the threshold for malignant transformation and early clonal dynamics probably contribute to strain-specific variation in tumour latency and incidence.

Our study in a highly controlled in vivo system reveals that the extent to which germline genetic variation shapes disease risk and development is far greater than previously appreciated. This raises challenging questions about whether germline genetic background should be considered alongside somatic alterations and environmental exposures in the design and interpretation of biomedical and translational cancer research.

## Methods

The key reagents and resources required to replicate our study are listed in Supplementary Table 2.

### Mouse colony management

Animal experimentation was carried out in accordance with the Animals (Scientific Procedures) Act 1986 (UK) and with the approval of the Cancer Research UK Cambridge Institute Animal Welfare and Ethical Review Body: the maximum approved tumour burden was 10% body weight, which was not exceeded. Animals were maintained using standard husbandry: mice were group-housed in Tecniplast GM500 IVC cages with a 12 h–12 h light–dark cycle (07:00–19:00) and ad libitum access to water, food (LabDiet 5058) and environmental enrichments.

The following mouse strains and species were used: *M. musculus domesticus* C3H/HeOuJ (C3H mice) and C57BL/6J (BL6), *M. musculus castaneus* CAST/EiJ (CAST) and *M. caroli* CAROLI/EiJ (CAROLI; Supplementary Table 2). For simplicity, these strains, subspecies and species are here referred to as ‘strains’.

### Chemical model of hepatocarcinogenesis

We treated 15-day-old (P15) male mice of all strains with a single intraperitoneal injection of DEN (N0258, Sigma-Aldrich; 20 mg kg^−1^ body weight) diluted in 0.85% saline. This is a well-established tumour-induction protocol and therefore DEN treatment was not randomized or blinded. Injections were performed in a fixed-time interval between 08:00 and 09:00 to control for circadian effects in DEN metabolism. Liver tumour samples were collected from DEN-treated mice 25 weeks (C3H), 36 weeks (BL6), 38 weeks (CAST) or 78 weeks (CAROLI) after treatment; pilot data indicated that 100% of surviving DEN-treated mice would develop tumours by these timepoints. The time of tumour removal was recorded for DEN-exposed BL6 mice, with 5-min intervals added for each sequential tumour isolated (Extended Data Fig. 9a–e). Our existing cohort of 370 C3H tumours^25^ included multiple tumours per animal and multiple animals per litter. To capture this hierarchical structure across genetic backgrounds, we included a minimum of 50 tumours per strain.

Untreated control mice from each strain were assessed for the presence or absence of tumours at the same ages as DEN-induced tumours to assess inherent susceptibility to spontaneous tumours (Fig. 1a and Supplementary Table 3). In addition, spontaneous liver tumours were collected from untreated mice identified opportunistically as part of routine colony health surveillance (including one female mouse; Fig. 1b and Supplementary Table 3). All macroscopically identified tumours were isolated and processed in parallel for DNA and RNA extraction and histopathological examination. Additional tissues from untreated P15 mice (ear, tail and liver), untreated age-matched adult mice (liver only; C3H 27 weeks, BL6 38 weeks, CAST 40 weeks and CAROLI 80 weeks) and DEN-treated background (non-tumour) liver (BL6 38 weeks) were sampled for control experiments.

### Tissue collection and processing

Liver tumours of sufficient size (2 mm or larger diameter) were bisected; one-half was flash frozen in liquid nitrogen and stored at −80 °C for DNA and RNA isolation, and the other half was processed for histology. Tissue samples for histology were fixed in 10% neutral-buffered formalin for 24 h, transferred to 70% ethanol, machine processed (Leica ASP300 Tissue Processo) and paraffin embedded. All formalin-fixed paraffin-embedded sections were 3 μm in thickness.

### Histochemical staining

Formalin-fixed paraffin-embedded tissue sections were stained with haematoxylin and eosin (H&E) using standard laboratory techniques. Histochemical staining was performed using the automated Leica ST5020; mounting was performed on the Leica CV5030.

### Whole-slide image acquisition

Tissue sections were digitized using the Aperio XT system (Leica Biosystems) at 20× resolution; all H&E images are available in the BioStudies archive at EMBL-EBI under accession numbers S-BSST383 (ref. ^25^) and S-BSST384 (this study).

### Tumour histopathology

H&E sections of liver tumours were blinded and assessed twice by a histopathologist (S.J.A.). Tumours were classified as dysplastic nodule or hepatocellular carcinoma (HCC) according to the International Harmonization of Nomenclature and Diagnostic Criteria for Lesions in Rats and Mice guidelines^55^. In addition, tumour grade, size, morphological subtype, nature of steatosis and mitotic index were assessed (Supplementary Table 1), as well as the presence of cystic change, haemorrhage, necrosis or vascular invasion.

### Sample selection for computational pathology, WGS and RNA-seq

Tumours that met the following histological criteria were selected for WGS (*n* = 370 for C3H, *n* = 55 for BL6, *n* = 84 for CAST and *n* = 72 for CAROLI): (1) diagnosis of dysplastic nodule, (2) homogenous tumour morphology, (3) tumour cell percentage of more than 70%, and (4) adequate tissue for DNA and/or RNA extraction. Neoplasms with extensive necrosis, mixed tumour types, a nodule-in-nodule appearance (indicative of a HCC arising within a dysplastic nodule) or contamination by normal liver tissue were excluded. As carcinogen-induced tumours arising in the same liver are independent^24^, multiple tumours were selected from each mouse to minimize the number of animals used. A subset of normal (non-tumour) samples from untreated age-matched mice were also whole-genome sequenced (*n* = 13 for C3H, *n* = 11 for BL6, *n* = 7 for CAST and *n* = 14 for CAROLI). In addition, a small number of HCC tumours were subject to WGS and released in the accompanying data, but were excluded from the analyses reported in this article (*n* = 1 for C3H, *n* = 11 for BL6, *n* = 0 for CAST and *n* = 4 for CAROLI).

### Computational pathology analysis

Whole-slide images (WSIs) of tumours were annotated in QuPath (v0.2.2)^56^ using the polygon tool to include neoplastic tissue and exclude adjacent parenchyma, cyst cavities, processing artefacts and white space. For tumours with multiple transections, only a single WSI was used. Annotations were reviewed for quality by two histopathologists (S.J.A. and J.C.). Annotated regions were tessellated into fixed size, non-overlapping 256 µm × 256 µm tiles using Groovy in QuPath. For segmentation of epithelioid nuclei, a pre-trained StarDist^57^ model (he_heavy_augment.zip) was downloaded (https://github.com/stardist/stardist-imagej/tree/master/src/main/resources/models/2D) and an inference instance was deployed using Groovy across the tiles in QuPath, built from source with Tensorflow^58^, with a minimum detection threshold of 0.5. Quantitative geometric features (size and shape) were measured for each nuclear object (Supplementary Table 1). The object-level measurements were subsequently abstracted by computing the median, standard deviation, interquartile range and kurtosis for each tile and WSI. Nuclear volume was subsequently calculated using the median nuclear diameter per slide.

Digitized histology images of DEN-induced tumours are available from Biostudies: accession numbers S-BSST383 (ref. ^25^) and S-BSST384 (this study).

### DNA and RNA isolation

Simultaneous isolation and purification of genomic DNA and total RNA from tumours, background DEN-exposed liver tissue and untreated normal liver tissue was performed using AllPrep 96 DNA/RNA kit (80311, Qiagen) according to the manufacturer’s instructions. DNA from P15 liver tissues was isolated and purified using the AllPrep DNA/RNA mini kit (80204, Qiagen); total RNA was extracted using QIAzol Lysis Reagent (79306, Qiagen) according to the manufacturer’s instructions. Genomic DNA was isolated from ear or tail samples using the DNeasy blood and tissue kit (69504, Qiagen) according to the manufacturer’s instructions.

### WGS

Genomic DNA quality was assessed on a 1% agarose gel and quantified using the Quant-IT dsDNA Broad Range Kit (Q33130, Thermo Fisher Scientific). Genomic DNA was sheared using a Covaris LE220 focused-ultrasonicator to a 450-bp mean insert size.

WGS libraries were generated from 1 μg of 50 ng µl^−1^ high-molecular-weight genomic DNA with a *Saccharomyces cerevisiae* spike-in control (1 ng of 900 pg µl^−1^ genomic DNA; 69240, Merck Millipore) using the TruSeq PCR-free Library Prep Kit (20015963, Illumina) according to the manufacturer’s instructions. Library fragment size was determined using a Caliper GX Touch with a HT DNA 1k/12K/Hi Sensitivity LabChip and HT DNA Hi Sensitivity Reagent Kit to ensure 300–800 bp (target of approximately 450 bp).

Libraries were quantified by real-time PCR using the Kapa library quantification kit (KK4824, Kapa Biosystems) on a Roche LightCycler 480. We pooled 0.75 nM libraries in six-plex and sequenced them on a HiSeq X Ten (Illumina) to produce paired-end 150-bp reads. Each pool of six libraries was sequenced over eight lanes (minimum of 40× coverage).

WGS FASTQ files are available in the European Nucleotide Archive at EMBL-EBI under accession PRJEB37808 (ref. ^25^) and PRJEB15138 (this study).

### WGS read alignment

Alignment of WGS data was performed as previously described^25^. In brief, sequencing reads were aligned to their respective genome assemblies from Ensembl (v.91)^59^, (BL6 = GRCm38 = GenBank: GCF_000001635.26, C3H = C3H_HeJ_v1 = GenBank: GCA_001632575.1, CAST = CAST_EiJ_v1 = GenBank: GCA_001624445.1, CAROLI = CAROLI_EiJ_v1.1 = GenBank: GCF_900094665.2) using bwa-mem (v0.7.12)^60^. The yeast reference genome S228c (GenBank: GCA_902192305.1) was included in the alignment targets to account for the spike-in DNA. Using WGS of non-tumour liver, ear and tail samples (described above) collected and sequenced contemporaneously with tumour samples, regions of abnormal WGS read coverage were identified^25^ and subsequently masked from the analysis (percentage of the reference genome masked: BL6 = 5.5%, C3H = 12.7%, CAST = 11.5% and CAROLI = 12.5%).

### Variant calling and mutation filtering

Single-base substitution (SBS) mutations were called using Strelka2 (v2.8.4)^61^. As described in ref. ^25^, fixed genetic differences and segregating germline variants within our colonies were identified and discarded. Indel mutations were filtered with identical parameters to SBS mutations with the additional criteria that (1) each called indel mutation must be supported by at least three independent reads, and (2) where the same indel mutation was found in at least two tumours from the same animal and shared between more than 50% of tumours from that animal, it was filtered from the calls of all tumours within the strain. This latter ‘animal-level-filtering’ step removes fixed and segregating germline indels that passed the familial clustering filter due to the conservative calling of indels relative to SBS mutations. Of the 4,446 indel mutations subject to animal-level filtering (distinct sites, not summing repeat occurrences over tumours), only two are predicted by Variant Effect Predictor (VEP; v109.3)^62^ to disrupt protein sequence (genes *Atxn2l* and *Cyp4a30b*).

### Mutational rate calculations

For lesion-strand resolved analyses, SBS mutational rates were calculated as 192 category vectors representing every possible single-nucleotide substitution conditioned on the identity of the upstream and downstream nucleotides. Each rate being the observed count of a mutation category divided by the count of the trinucleotide context in the analysed sequence. For lesion-strand non-resolved analyses, the same procedure was followed but reverse complement mutation (for example, T→C and A→G) and sequence context (for example, ATG and CAT) counts were combined to give 96 category vectors, which by convention are presented oriented as a mutation from the pyrimidine base of the base pair. Indel rates were calculated as indel count per megabase, per tumour and corrected for the expected diploidy of autosomes.

### Mutational signature analysis

SBS mutational signatures were defined and deconvolved using the R NMF library (v0.28) nmf function^63^ with rank = 5, run =1,500 and the method set to use the Brunet algorithm^64^. The rank defines the number of signatures to identify; the value of 5 was selected after testing ranks 2 to 8 inclusive and selecting the first value of rank for which the cophenetic coefficient substantially decreases^64^. This value also coincided with an inflection point in the curve plotting the residual sum of squares against rank, as proposed as an alternate method for optimal rank choice^65^.

Indel mutational signatures were generated using SigProfiler (v1.2.19)^66^ using the corresponding strain-specific reference assemblies (masked, see above). Bootstrap replicate datasets were generated using the SigProfilerMatrixGenerator for the calculation of 95% confidence intervals. For the analysis of multi-nucleotide indel sequence composition, only tumours with cellularity estimated at more than 50% were considered and indels of length greater than 1 bp were folded into unique discrete sequence categories combining repeat unit repetition and reverse-complement relationships, for example, AC deletion encompasses AC, CA, GT and TG deletion. Insertions and deletions were categorized by length (span of deleted or inserted nucleotides) and the mutational rates for each sequence category (for example, AC) calculated as the number of events (for example, AC deletions) divided by the number of that sequence context in the reference genome (for example, AC occurrences). Percentage rates were calculated (for example, AC deletion rate as a percent of the sum of dinucleotide category deletion rates) and compared between strains (Extended Data Fig. 1o–q); the comparison of rates thus controls for the minor sequence composition differences between the genomes of the four strains. The 95% confidence intervals were obtained from 100 bootstrap samples of the tumours for analysis within each strain.

### Mutational asymmetry segmentation and scoring

The lesion segregation model predicts that whole chromosomes would be coherently strand asymmetric for mutations following a burst of DNA damage. Although this is often the case, homologous repair in the first round of DNA replication following damage can result in sister chromatid exchange (SCE) events and discrete transitions in the mutational asymmetry of the chromosome such that chromosomes are made up of multi-megabase blocks of alternating mutation asymmetry^25,26^. We refer to these blocks as genomic segments. Genomic segmentation on mutational asymmetry was performed as previously reported^25^. Mutational strand asymmetry was scored for each genomic segment using the relative difference metric *S* = (*R*_T_ *−* *R*_A_)/(*R*_T_ + *R*_A_) where *R*_T_ is the rate of mutations from T on the forward (plus) strand of the reference genome and *R*_A_ is the rate of mutations from A on the plus strand, equivalent to the rate of mutations from T on the reverse (minus) strand.

### Identifying and filtering reference genome mis-assemblies

Mutational asymmetry patterns arising from lesion segregation allow the long-range phasing of chromosome strands, which can be used to detect discrepancies in sequence order and orientation between the sequenced genomes and the reference. We identified autosomal asymmetry segments that immediately transitioned from forward strand bias (*S* > 0.33) to reverse strand (*S* < −0.33) or vice versa without occupying the intermediate unbiased state (−0.33 ≥ *S* ≤ 0.33); such discordant segments are unexpected. Allowing for ±100-kb uncertainty in the position of each exchange site, we produced the discordant segment coverage metric. At sites with discordant segment coverage of more than 1, we calculated relative enrichment for misassembly *M* = (ds − cs)/(ds+cs) where ds is the number of discordant segments over the exchange site and cs the number of concordant: where either forward or reverse mutational asymmetry extends at least 1 × 10^6^ nucleotides on both sides of the exchange site. Values of *M* > 0 indicate consensus for misassembly. The approximate genomic coordinates for a C3H strain-specific inversion on chromosome 6 have been previously reported^67^.

### Classification of mutational asymmetry

On the basis of previously described mutational asymmetry segmentation and scoring, samples were classified based on their adherence to the expectations of lesion segregation, that is, asymmetric or non-asymmetric^25^. First, to reduce noise and minimize the influence of large copy number alterations (CNAs) and aneuploidies, genomic segments with fewer than 100 mutations and those with significantly higher or lower than average read depth were removed (*P* < 0.05, probability of each segment being sampled from a normal distribution with mean and standard deviation equal to the weighted mean and standard deviation of the per-segment means). Genomic segments were classified as symmetric (abs(*S*) < 0.2, where abs(*S*) is the absolute value of the previously defined mutational asymmetry parameter *S*), asymmetric (abs(*S*) > 0.8) or intermediate (abs(*S*) ≥ 0.2 and ≤0.8). For each tumour, the proportion of the genome belonging to each class was calculated. Samples with less than 10% asymmetric autosomes were defined as non-asymmetric (symmetric). All remaining samples were classified as asymmetric. As SCE events can be identified only in the presence of mutation asymmetry, the non-asymmetric tumours were excluded from SCE rate calculations.

### Tumour cellularity estimates

As described in ref. ^25^, variant allele frequency (VAF) was calculated as (1 − *R*/*d*), where *R* is the reference read count at a mutated site and *d* is the total read depth at the site. For each tumour, the modal VAF was determined using the function ‘amps’ from the R package modes (0.7.0) to detect the size and position of the largest peak in the VAF density distribution. Cellularity was then calculated as ploidy × the major peak VAF, in which ploidy was set as 2 for all samples, except CAROLI tumours with no mutational asymmetry, which were inferred to be whole-genome duplicated (ploidy = 4).

### Large copy number variant calling

Tumour genomes were segmented on the basis of their change in read depth, relative to untreated adult liver samples (*n* = 11 for BL6, *n* = 11 for C3H, *n* = 14 for CAROLI and *n* = 7 for CAST) using CNVkit (v0.9.6)^68^. To suppress calling of small CNAs, minimum segment size was set as 1 Mb. For each segment, copy number (CN) was calculated using the log_2_ read depth fold change (FC) given by CNVkit, and the sample ploidy (*p*) and cellularity (*c*), where: CN = ((2^FC^ − 1 + *c*)/*c*) × *p*. Within strain, CN estimates were adjusted for read depth biases by subtracting the median change in CN of all overlapping segments (overlapping defined as segments where the intersect is 80% or more of the union, adjustment performed for segments with 3 or more overlapping segments, change in CN calculated as CN − *p*). Segments with adjusted CN within 0.15 of the sample ploidy were discarded. Segments with adjusted CN within 0.15 an integer were called as clonal CNAs. Segments outside this threshold were identified as subclonal CNAs. Filtering was then performed to remove recurrent artefactual CNAs by discarding any short CNA (less than 10% of total chromosomal length) with 80% or more overlap of another CNA from the same strain.

Aneuploidies were identified as either gain or loss of chromosomal segments totalling more than 80% of a chromosome; typically these were whole-chromosome (100%) gains and losses. Significant recurrence was identified by randomly permuting aneuploidy labels across autosomes. *P* values were calculated by comparing the observed recurrence of a specific aneuploidy to the null distribution of random recurrence generated by 10,000 permutations. Aneuploidies with *P* < 0.05 Bonferroni-corrected significance were identified as having significant recurrence.

### Telomere length analysis

Two complementary approaches were used for telomere length analysis. First, telomerecat (v4.0.1)^69^ was applied using default settings to whole-genome-sequenced samples aligned to the strain-matched reference genome (see above). The telomere length metric was estimated for each sample, which attempts to take into account ploidy changes, other changes in telomere count and interstitial (non-telomeric) telomere repeats in the reference genome. However, the quality of genome assembly systematically differs between the four strains in this study so a second, reference alignment free, approach was used to allow direct comparison between strains without confounding assembly differences. Using samtools view (v1.9)^70^ and Unix shell scripting (grep), WGS reads were identified that contained three perfect consecutive matches to the telomeric repeat sequence (TTAGGG)_3_ or its reverse complement (CCCTAA)_3_. All instances of the single TTAGGG or CCCTAA repeat were counted in, and summed over, those reads. The raw counts of telomere repeats were normalized to 1× haploid genome coverage using read alignment to the reference genome. Telomerecat and grep-based estimates of telomere length were highly correlated (Spearman’s *ρ* = 0.616, *P* = 1.54 × 10^−64^), but a small number of extreme discrepancies were identified that represented either zero-length or implausibly large telomeres reported by telomerecat. For these reasons, and the fair direct comparison between strains, the main results are reported using the grep-based method, but qualitatively, the same conclusions can be drawn from the telomerecat-based analysis.

### Multiallelic mutational rates

Aligned reads spanning genomic positions of somatic mutations were re-genotyped using samtools mpileup (v1.9)^70^. Genotypes supported by two or more reads with a nucleotide quality score of 20 or more were reported, considering sites with two alleles as biallelic, those with three or four alleles as multiallelic. To simplify analysis and interpretation, multiallelic rates were calculated only for mutations from A or T nucleotides (C and G lesions contribute to both DEN1 and DEN2 signatures and have differing propensities for multiallelic variation, and the signatures vary in their contributions between tumours). The multiallelic rate is the fraction of mutations from A or T in a tumour that are identified as multiallelic in that tumour. The multiallelic rate was calculated (1) as an average for each tumour, and (2) separately for each mutation asymmetry segment of each tumour.

### MRCA generation

Following previous work, we estimated cell generation post-mutagenesis of the MRCA of the cells in the sequenced tumour based on the fraction of autosomal genomic segments that are multiallelic^25^. Genomic segments were defined per tumour using mutational asymmetry, as described above. After the first cell division post-damage, each segment will contain two lesion-containing strands, which will be diluted through subsequent divisions. The scenarios where one or both lesion strands of a segment are multiallelic are not readily distinguishable from the data, as in both cases the segment will appear as multiallelic. However, the fraction of non-multiallelic segments, where both lesion strands are lost, is directly observable from the data. We called a segment non-multiallelic if less than 4% of mutated sites are multiallelic. Let *p* be the fraction of autosomal lesion containing strands, and *q* = 1 − *p*. Assuming independent segregation of DNA copies per segment, the expected fraction of autosomal segments without multiallelism (MA) is *q*^2^, and thus we estimate *p* as 1 − (fraction of segments without MA)^½^.

Tumours with more than 75% or 0% of segments showing multiallelism were assigned generation 1 and generation ≥5 MRCAs, respectively. For the remaining tumours, we estimated the MRCA generation using maximum likelihood under the following rationale. At generation 1 post-mutagenesis, we expect 100% of segment copies to have a lesion-containing strand and thus show multiallelism. At each subsequent generation, a segment loses one of its lesion-containing strands, and thus its ability to generate multiallelism with probability 0.5: hence, the probability of retaining an individual lesion strand at generation *n* post-mutagenesis is 2^(−*n* + 1)^. With independent segregation, the probability of no lesion strands in a segment at generation *n* is thus (1 − 2^(−*n* + 1)^)^2^. In a tumour with *x* autosomal segments, assuming segment independence, the observed segment number without multiallelism is thus distributed as binomial (*x*, (1 − 2^(−*n* + 1)^)^2^). Ranging over *n* = 2, 3, 4, 5, we selected the *n* value that maximizes the likelihood of the observed data under this binomial model, resulting in an inference of the MRCA generation of the tumour.

### Uniformity of post-MRCA growth

To quantify the balanced:unbalanced contribution of lineages post-MRCA (uniformity of clonal growth), we first identified genomic segments where the two allelic copies of a chromosome have opposite mutational asymmetries, resulting in the segment as a whole being mutationally symmetric (−0.5 ≤ *S* ≤ 0.5; more conservative asymmetry threshold used based on simulated data). In these mutationally symmetric regions, T→N mutations relative to the reference genome forward strand represent mutations from one lesion strand and A→N mutations represent the other lesion strand of the segment. Calculating multiallelic rates separately for T→N and A→N allows determination of the multiallelic rate resulting from each lesion strand of a segment. Under the rationale that unbalanced lineage contributions would result in higher variation in multiallelic rate (Fig. 5a), within these segments, we further split mutations into non-overlapping windows of 100 neighbouring T→N or A→N mutations, which allowed us to test for balanced retention of lineages within individual tumours. Our test requires only a single assumption, which is that the probability that a mutated site is multiallelic is constant across all sites. Under this assumption and balanced lineage retention, among segments that display multiallelism, the number of multiallelic mutant sites per window follows a truncated binomial distribution, TruncBinom(*n*, *p*, *a*), where *n* = 100, *p* is the probability of multiallelism per mutated site, and *a* is the threshold for a window to be deemed to have non-zero multiallelism (four or more multiallelic sites). Deviations from this distribution indicate non-uniform lineage retention. To test for non-uniform lineage retention, we calculated the overdispersion of the observed windowed multiallelic rates: the variance of these rates divided by the variance expected under the maximum-likelihood best-fitting truncated binomial distribution. Only windows within mutationally symmetric regions and with multiallelic rate ≥ 4% were considered.

To assess the power of this method and its robustness to SCEs, we performed a simulation study. We simulated DEN-induced tumours with generation 1, 2 and 3 MRCAs, and with different numbers of SCEs. We considered both SCEs that occurred at generation 0, which are detectable with mutational asymmetry segment calling, and SCEs that occurred after the first mitotic division, which are undetectable via asymmetry and henceforth termed ‘cryptic SCEs’. We simulated non-uniform lineage retention via selection by varying F, the proportion of the final tumour contributed by the clonally dominant daughter cell of the MRCA from 0.5 (neutral evolution) to 0.9 (highly biased lineage retention). Tumours were simulated for seven generations after transformation, with retained damage generating mutations at each round of replication, according to mutational probabilities matching the observed DEN mutations. We set the probability of correctly incorporating the reference base opposite damage in a single replication to be 0.75, which best matched the typical observed multiallelic rates in our data. We then simulated sequencing at 30× coverage, and applied the same pipeline of calling multiallelic and performing segment calling to the simulated data as to the mouse data. For each simulated tumour, we calculated the overdispersion, and used Monte Carlo simulation of the overdispersion from the best-fitting truncated binomial distribution to test for non-uniform lineage retention (10,000 Monte Carlo simulations per tumour).

In simulations, we observed that cryptic SCEs could potentially inflate our false-positive rate by acting as an alternative, non-uniform mechanism to increase the variance in multiallelic rates. Therefore, we performed a stringent filtering of windows to remove any windows that potentially overlapped a cryptic SCE site, identifying as candidates windows with non-zero multiallelic rate (4% or more) that occurred next to one or more windows with zero multiallelic rate (less than 4%). As this strategy does not account for cryptic SCEs where the breakpoint occurs on the first or last window of a segment, we also removed the first and last window of each type from each segment in our filtering (which resolved this issue in our simulations). The multiallelic overdispersion was calculated for *n* = 389 tumours, including tumours with inferred generation 1, 2 or 3 MRCAs and that had at least two windows that satisfied the lesion-strand-filtering conditions. For each of these tumours, we then performed Monte Carlo simulations, simulating 1 million draws from the best-fit uniform-growth distribution to each tumour, calculating a *P* value of our observed overdispersion, which we then Bonferroni corrected, and used to test the null hypothesis, at 0.05 significance, that the tumour grew with uniform retention of lineages. We also note, from simulations, that the value of this overdispersion statistic provides an indication of the extent of biased lineage retention that takes place (Extended Data Fig. 10b).

### Genomic annotation

Genic annotation was obtained from Ensembl (v91)^59^ for the corresponding C3H, BL6, CAST and CAROLI reference genome assemblies (C3H_HeJ_v1, GRCm38, CAST_EiJ_v1 and CAROLI_EiJ_v1.1, respectively). Genomic repeat elements were annotated using RepeatMasker (v20170127)^71^ with the default parameters and libraries for mouse annotation. Genomic coordinates were transformed between reference genomes using the HAL toolkit (v2.3)^72^, utilizing the UCSC mouseStrains_1509.hal multi-strain alignment with CAROLI_EiJ_v1.1 added using progressiveCactus (v1.0.0)^73^. The functional consequences of mutations (for example, amino acid change, splice site disruption and synonymous sequence change) were annotated using Ensembl VEP (v109.3) run in the ensemblorg/ensembl-vep docker container under Singularity; the Ensembl v91 annotation was used for the corresponding reference genome assembly. Phylogenetic distances between strains were calculated using fourfold degenerate (4D) sites, extracted from the HAL alignments using the HAL toolkit hal4dExtract function. 4D site rate calculations were performed using PHAST (v1.3)^74^ phyloFit under the REV model.

Extrapolating from substitution rates at codon 4D sites (Fig. 1a), there are expected to be 7.5 × 10^6^ single-nucleotide differences genome wide between C3H and BL6. Between BL6 and CAST, 2.5 × 10^7^ single-nucleotide differences are expected. For comparison, between any two randomly selected humans, there are expected to be between 6.4 × 10^6^ and 2.5 × 10^7^ single-nucleotide differences^33^, placing most human comparisons between BL6:C3H and BL6:CAST in the number of genetic differences.

### Driver discovery methods

Protein-coding cancer driver genes were analysed using three computational methods: OncodriveFML (v2.2.0)^75^, which identifies genes with a bias towards high impacting mutations, OncodriveCLUSTL (v1.1.3)^76^, which identifies genes containing mutations that are linearly clustered in nucleotide sequence, and dNdScv (v0.1.0)^77^, which uses a maximum-likelihood approach to quantify selection by identifying genes with an excess of nonsynonymous, essential splice site or truncating mutations.

To increase the sample size, a single pan-strain analysis was performed using all DEN-treated dysplastic nodules (*n* = 581). Only single-nucleotide mutations that mapped to all four genomes were kept (*n* = 32,894,997, HAL alignments above). For the OncodriveFML and OncodriveCLUSTL analyses, mm10 was used as the reference genome. The dNdScv analysis was carried out four times; in each iteration, mutations were projected onto the gene annotations of one of the four mouse strains.

To run OncodriveFML and OncodriveCLUSTL, protein-coding sequences from all transcripts in protein-coding genes were merged together into their corresponding gene using pybedtools (v0.8.0)^78^.

OncodriveFML variant pathogenicity was assessed using GRCm38/mm10 SIFT scores (v83)^79^. OncodriveFML was run on the pan-strain dataset using strain-specific coding DNA sequence (CDS) normalized trinucleotide background models. These mixed background models allow the method to account for strain-specific differences in the trinucleotide mutational probabilities. These models were computed using bgsignature (v0.2; https://pypi.org/project/bgsignature/). All other OncodriveFML parameters were kept as default. Genes with *q* < 0.1 were considered candidate drivers.

OncodriveCLUSTL was run using a strain-specific CDS normalized trinucleotide background model, as for OncodriveFML. To improve the accuracy of clustering signals detected in CDS regions, simulated mutations were randomly sampled within coding sequences (simulation mode ‘region_restricted’). Clusters were analysed by concatenating all subsequent CDS regions of a gene. All other parameters were kept as default. Genes with *q* < 0.01 bearing more than 15 mutations were considered candidate drivers.

To run dNdScv, genome references were built using the GTF (Ensembl v91) annotations of the respective mouse strain. dNdScv was run with the parameters ‘cv = NULL’ and ‘max_muts_per_gene_per_sample = 50’ and genes with *q* < 0.1 were identified.

### Driver event annotation and combinatorial analysis

Candidate driver events including point mutations, protein-coding sequence disrupting indels, WGD and recurrent aneuploidies were encoded as binary variables (either present or absent) for each tumour. For nucleotide substitution and indel mutations any ‘moderate’ or ‘high’ impact mutation annotation in an identified driver gene was considered to be a candidate driver mutation. Where VAF filtering of driver mutations was applied, a tumour was considered to have a driver mutation if the VAF of that mutation exceeded (*c*/2)/*p*, where *c* is the cellularity estimate of the tumour and *p* is the expected ploidy of the driver locus (autosomal = 2, X chromosome = 1, double those values in WGD tumours).

Evolutionary dependency (co-occurrence and mutual exclusivity) was scored using SELECT (v1.6)^80^ with default parameters. The wMI *P* value was used for colour-coding significance and false discovery rate < 0.1 used as multi-testing corrected threshold of significance^38^. Comparison of driver mutation proportions between strains were performed using the *χ*^2^ test implemented in the built-in chisq.test function of R, based on contingency tables of mutation counts.

### RNA-seq

Tumour RNA was isolated and purified as described above. RNA concentration was measured using a NanoDrop spectrophotometer (Thermo Fisher); RNA integrity was assessed on a Total RNA Nano Chip Bioanalyzer (Agilent).

Total RNA (1 μg) was used to generate sequencing libraries using the TruSeq Stranded Total RNA Library Prep Kit with Ribo-Zero Gold (20020596 and 20020599, Illumina), according to manufacturer’s instructions. Library fragment size was determined using a 2100 Bioanalyzer (Agilent). Libraries were quantified by quantitative PCR (Kapa Biosystems). Pooled libraries were sequenced on a HiSeq4000 to produce 40 million or more paired-end 150-bp reads per library.

### RNA-seq data processing and analysis

Transcript abundances were quantified with Kallisto (v0.43.1)^81^ (using the flag–bias) and a transcriptome index for each strain compiled from coding and non-coding cDNA sequences defined in Ensembl (v91)^59^. Expression patterns among the four strains were identified through PCA by combining unnormalized counts for protein-coding genes with orthologues in GRCm38 (Fig. 4a), and per strain from all annotated protein-coding genes and long intergenic non-coding RNAs (Extended Data Fig. 7e,f). The 500 most variable genes were selected in both cases. Transcripts per million estimates were generated for each annotated transcript and summed across alternate transcripts of the same gene for gene-level analysis. Transcription start sites for each gene were annotated with Ensembl (v91) and based on the most abundantly expressed transcript.

RNA-seq data are available at ArrayExpress at EMBL-EBI under accession E-MTAB-8518 (ref. ^25^) and E-MTAB-16391 (this study).

### Differential gene expression analysis

Differential expression was called using DESeq2 (ref. ^82^) at the gene level after importing estimated counts per transcript from Kallisto using tximport^83^. Calling was restricted to protein-coding genes and long intergenic non-coding RNAs. Pairwise comparison of differentially expressed genes across strains was performed on the intersection of significantly differentially expressed genes from per-strain dysplastic nodule versus adult normal comparisons (adjusted *P* ≤ 0.005) after mapping strain-specific IDs to GRCm38 (Extended Data Fig. 7e,f).

### Independent component analysis of gene expression

Independent component analysis (ICA) was performed using BIODICA (v0.9)^84^. The optimal number of components was chosen by BIODICA based on *n* = 100 iterations of ICA. ICA was applied separately to DEN-induced tumours and to normal (non-tumour) liver samples; for both datasets, expression counts were subject to variance stabilizing transformation before ICA clustering. The Bioconductor package clusterProfiler (v4.14.0)^85^ was used to identify Gene Ontology and Kyoto Encyclopedia of Genes and Genomes (KEGG) terms over-represented in the high-contribution (key) genes, defined as at least 3 standard deviations from the mean ICA contribution of all genes. For each independent component, we used the annotation with the greatest degree of enrichment (ratio of the number of key genes with the annotation to the number of all genes with the annotation) that had more than five key genes with that annotation and significant term enrichment (Benjamini–Hochberg adjusted *P* < 0.05).

Enrichment of independent component signatures was evaluated through multinomial log-linear modelling using the R package nnet (v7.3) function multinom with the brglmFit model from the R package brglm2 (v0.9.2). Models were specified including independent component latent factor enrichment and strain identifier. Signatures IC6, IC7 and IC17 were redundant as they closely correlated with strain so were specifically excluded from the model. A MAPK pathway driver gene (or specific mutation for *Hras* analysis) was defined as the response variable and odds ratios calculated relative to a reference driver (*Braf* for gene analysis and *Hras*^*Q61K*^ for *Hras* analysis). Statistical tests were Bonferroni corrected for 42 tests in the driver gene-based comparisons and 28 tests in the *Hras* analysis.

### MAPK pathway gene expression analysis

Seventy genes in the MAPK pathway (KEGG pathway: mmu04010 (ref. ^86^)) were found to be differentially expressed in all strains in a *Braf*-driven tumour versus adult, age-matched normal liver tissue comparison. Fold changes of these genes, where significant, in *Hras*-driven and *Egfr*-driven tumours was consistent with those in *Braf*-driven tumours (Fig. 4b). Differential expression was called as above, with restriction to tumours with identified driver genes. The expression of the 70 MAPK genes in the combined four-strains dataset described above were used in PCAs (Extended Data Fig. 7g).

### Expected mutational rate modelling

To model the expected rate (mutations per million nucleotides) of a specific mutation in a focal gene (for example, *Hras*^*Q61L*^ is a T→A substitution in a GTT context with respect to the transcription template strand), we identified the 1,000 genes with the closest measured expression (transcripts per million) to the focal gene in the P15 liver RNA-seq. In aggregate for these nearest-neighbour genes, 192 category lesion-strand-resolved mutational rate vectors were calculated (see above) separately for transcription on (1) the lesion-containing template, and (2) lesion-containing non-template genic strands^28^. These rate vectors contain the rate per million nucleotides for each type of substitution (for example, *R*_GTT→GAT_ for the template strand and *R*_AAC→ATC_ for the non-template strand lesions in *Hras*^*Q61L*^). For a set of tumours (for example, *n* = 370 C3H tumours), we counted the number of template-retained (*S*_t_) and non-template-retained (*S*_n_) lesion strands at a focal locus (for example, *Hras* codon 61) and used the ratio of template:non-template lesion strands to calculate an expected mutation rate for the specific focal mutation (for example, expected rate *μ* = (*R*_GTT→GAT_*S*_t_ + *R*_AAC→ATC_*S*_n_)/(*S*_t_ + *S*_n_)). To estimate uncertainty, we calculated the 95% confidence intervals from 100 bootstrap samples of the tumour set. The same bootstrap sampling of tumours provided 95% confidence intervals on the observed mutational counts.

The expected proportions of MAPK driver mutations for a set of tumours (for example, *n* = 370 C3H tumours) was calculated as *μ*_*i*_/∑*μ*_(1..*n*)_ where *μ*_*i*_ is the expected rate of the focal mutation and ∑*μ*_(1..*n*)_ is the sum of the expected rates of for all considered MAPK driver mutations (*Hras*^*Q61R*^, *Hras*^*Q61L*^, *Hras*^*Q61K*^, *Kras*^*Q61R*^, *Egfr*^*F254I*^ and *Braf*^*V637E*^). The 95% confidence intervals for the expected proportions were calculated from 100 bootstrap samples of the tumour set.

### Predicting the immune presentation of mutations

For every VEP annotation of a substitution mutation (see above) that altered the protein-coding sequence, the corresponding amino acid change was edited into the translated peptide sequence and all possible overlapping 8-amino acid, 9-amino acid, 10-amino acid and 11-amino acid oligopeptides were presented to MHCflurry (v2.1.1)^87^ and netMHCpan (v4.1)^88^ for MHC class Iα affinity prediction. MHCflurry was used with pre-trained affinity models that correspond to the BL6 (*H-2-Kb* and *H-2-Db*) and C3H (*H-2-Kk* and *H-2-Dk*) class Iα MHC genes. No pre-trained MHCflurry models are available for CAST or CAROLI class Iα MHC genes.

The netMHCpan tool does allow for prediction even when the exact MHC class Iα gene was not included in the training set^88^. Strain-specific MHC class Iα gene sequences for netMHCpan were defined as follows. The genomic sequences of the *H2-D1* and *H2-K1* genes, plus flanking sequence were queried from the mm39 genome reference using Bedtools: (v2.30.0)^89^ (*H2-D1* = chromosome 17: 35480951–35487230, *H2-K1* = chromosome 17: 34213953–34220395). Orthologous sequence from each of the strains was found by querying the mm39 sequences against unannotated long-read-based genome references (BL6 = C57BL_6NJ_v2, C3H = C3H_HeJ_v2, CAST = CAST_EiJ_v2, available from NCBI, BioProject (PRJEB47108)) using BLAST (v2.5.0)^90^. To predict the protein sequence, MHC class Iα protein sequences were obtained from UniProt^91^ (P01899 for H-2-Db, P14426 for H-2-Dk, P01901 for H-2-Kb and P04223 for H-2-Kk) and projected onto the H2-D1 and H2-K1 sequences using GeneWise^92^. Sequences were manually refined to ensure the presence of start and stop codons, and resemblance to previously published exon boundaries. For the more divergent CAROLI strain, MHC class Iα protein sequences were obtained from NCBI RefSeq (BioProject PRJNA387030); in contrast to the other strains, CAROLI has three annotated MHC class Iα genes (*H2-K*: XP_029329709.1, *H2-D*: XP_021009871.1 and *H2-L*: XP_029326886.1).

The maximum affinity score (lowest rank_EL for MHCflurry; maximum pan_EL score for netMHCpan) of the multiple alternate overlapping peptides for each mutation was recorded and used in subsequent analysis. Note that mutations of every strain were scored against each of the MHC class Iα of every strain (for example, BL6 mutations scored against each MHC class Iα gene from each of C3H, BL6, CAST and CAROLI). Affinity scores were quantile normalized for comparison to previously computed distributions of test peptides, as recommended^87,88^, and scores within the top 0.5% of test peptides were considered to be high-affinity predicted binders. Normalized, maximum affinity scores were used for driver mutation immunogenicity prediction (Extended Data Fig. 5).

As, for example, a C3H MHC molecule can facilitate the immune-mediated removal of mutations only in C3H mice and not BL6, CAST or CAROLI mice, we tested for general evidence of immune editing by contrasting strain-matched versus non-strain-matched distributions of epitope affinity scores. We define the fraction of all (global) mutations that come from a focal strain as *G*_f_ (for example, C3H mutations/(C3H + BL6 + CAST + CAROLI) mutations, for focal C3H). We produced a combined rank of affinity scores for global for each MHC class Iα gene, and take 1,000 mutation consecutive windows over the rank affinity scores, for each window calculating the (local) fraction of mutations from the focal strain *L*_f_. The relative enrichment (RE) of mutations from a strain in the window is calculated as RE = (*L*_f_ − *G*_f_)/(*L*_f_ + *G*_f_), a metric bounded (1,−1) where RE = 0 for no enrichment. For the highest predicted affinity windows, immune editing would be predicted to lead to RE < 0 (depleted mutations) where the focal strain matches the MHC class Iα gene used in affinity prediction. The results presented are restricted to mutations in expressed genes (median tumour expression transcripts per million > 1.0) as expression is expected to be a prerequisite for immune presentation; however, the same conclusions can be drawn from analyses that do not filter on expression level. Confidence intervals (95%) were calculated from 10,000 random permutations of the affinity rank list. The analysis was repeated for all combinations of strain and each MHC class Iα gene, none showed compelling evidence supporting the extensive immune editing of neoepitopes; example analyses are shown (Extended Data Fig. 5c). Positive controls were provided by the computational subtraction of a defined percentage (4%, 2%, 1% or 0.2%) of high predicted affinity mutations, selected by sampling predicted weak and strong binders (top 5% normalized affinity) weighted by normalized affinity score (better predicted binders are more likely to be selected for removal).

### ChIP–seq

Livers from P15 mice were perfused in situ with PBS and then dissected, minced, cross-linked using 1% formaldehyde solution for 20 min, quenched for 10 min with 250 mM glycine and washed twice with ice-cold PBS, and tissue pellets were stored at –80 °C. Tissues were homogenized using a dounce tissue grinder, washed twice with PBS and lysed according to published protocols^93^. Chromatin was sonicated to an average fragment length of 300 bp using a Misonix tip sonicator 3000. To negate batch effects and allow multiple ChIP experiments to be performed using the same tissue, we pooled ten livers for each mouse strain; 0.5 g of washed dounced tissue was used for each immunoprecipitation. The following antibodies were used for each ChIP experiment: CTCF (rabbit polyclonal, 07-729, Merck Millipore; 20 μg), H3K4me3 (mouse monoclonal IgG clone CMA304; 05-1339, Merck Millipore; 10 μg) and H3K27ac (rabbit polyclonal IgG; 4729, Abcam; 10 μg). Immunoprecipitated DNA or input DNA (maximum of 50 ng) was used for library preparation using the ThruPLEX DNA-Seq library preparation protocol (R400676, Rubicon Genomics). Library fragment size was determined using a 2100 Bioanalyzer (Agilent). Libraries were quantified by quantitative PCR (Kapa Biosystems). Pooled libraries were sequenced on a HiSeq4000 (Illumina) according to the manufacturer’s instructions to produce paired-end 150-bp reads. All experiments were performed with a minimum of three biological replicates.

### ChIP–seq data processing and peak calling

To identify ChIP–seq-positive regions, sequencing reads were trimmed to 50 bp and then aligned to their respective genome assemblies (BL6 = GRCm38, C3H = C3H_HeJ_v1, CAST = CAST CAST_EiJ_v1 and CAROLI = CAROLI_EiJ_v1.1) using bwa (v0.7.17)^60^ using default parameters. Uniquely mapping reads from each library were selected for further analysis. Peaks were identified for each ChIP library using MACS (v2.1.2)^94^ and matched input controls; for histone modifications, the ‘–broad’ flag was used. For CTCF and histone modifications, all peaks with *q* < 0.05 were included. We used the input libraries to filter spurious peaks associated with a high input signal using the GreyListChIP R package (v1.36.0)^95^. Biologically reproducible peaks were identified by merging ChIP–seq peaks (as defined above) from individual replicates and selecting those genomic regions found in two or more replicates.

Enhancers and promoters were defined using the sets of biologically reproducible H3K4me3 and H3K27ac peaks following the overlap rules previously defined^96^. In brief, promoters were defined as H3K4me3 regions, with or without overlapping H3K27ac. Enhancers were defined as H3K27ac regions that did not overlap a H3K4me3 region. Finally, as regions of abnormal read coverage were masked for mutation detection (previously described), all ChIP–seq regions overlapping these regions were also removed from downstream analyses.

### Regulatory region mutational rate calculation

Genomic coordinates were transformed between reference genomes using the HAL toolkit (v2.3)^72^, utilizing the UCSC mouseStrains_1509.hal multi-strain alignment with CAROLI_EiJ_v1.1 added using progressiveCactus (v1.0.0)^73^. Regions mapping to multiple scaffolds and overlapping alignments were removed, and the longest alignment for each region was identified. These 1:1 alignable regions were then overlapped with regulatory region annotation (above) in their corresponding strain.

For a given regulatory region type (CTCF-binding sites, enhances or promoters), we calculated the weighted-mean mutational rate of single-nucleotide variants across all trinucleotide contexts. Mutational rate was calculated on a per-tumour basis as the fraction of each trinucleotide in the aggregated genomic span of a group of regions (for example, promoters) that are mutated, weighted by the frequency of that trinucleotide in the genome (including regions masked for abnormal read coverage in that strain). To compare the influence of CTCF binding and regulatory site activity on mutational rate between strains, for each strain, we calculated a relative enrichment metric. We compared the CTCF bound or active promoters or enhancers with their shadow sites (that is, sites with orthologous sequences to bound or active sites in other strains but not identified as bound or active in the focal strain) (Extended Data Fig. 6e). Rates were calculated in aggregate over the tumours of a focal strains but individual binding and shadow sites were sampled with replacement 1,000 times to calculate bootstrap confidence intervals. The relative enrichment metric (*μ*RE) was calculated as *μ*RE = (*μ*_active_ *− μ*_shadow_)/(*μ*_active_ + *μ*_shadow_);* μ*RE > 0 shows that active site mutation rate is greater than the shadow rate, *μ*RE < 0 shows the opposite and *μ*RE = 0 denotes equal rates. Comparisons include separate calculations for (1) where the active sites are conserved across all four strains (four-way conserved), and (2) where they are active in the focal strains but not detected as active in all four strains (partially conserved). Statistical tests for difference in mutational rate between active and shadow regions were implemented as Wilcoxon matched-pairs signed-rank test using the R wilcox.test function, with aggregate active and aggregate shadow regions within a tumour as the matched-pairs; *P* values were Bonferroni corrected for multiple testing (*n* = 24 tests).

ChIP–seq data (FASTQ files and peak calls) are available in ArrayExpress at the EMBL-EBI under accession E-MTAB-11959 (ref. ^28^) and E-MTAB-14454 (this study).

### ATAC-seq

ATAC-seq protocol for the frozen tissue was used as previously described^97^, with minor modifications to the nuclear isolations steps. In step 1, 1 ml of 1× homogenizer buffer was used instead of 2 ml, and in step 4, douncing was performed with 30 strokes instead of 20 (ref. ^28^). Pooled libraries were sequenced on a NovaSeq 6000 (Illumina) to produce paired-end 50-bp reads, according to the manufacturer’s instructions. Experiments were performed with at least three biological replicates.

ATAC-seq was also performed on DEN-treated BL6 tissues: three pairs of DEN-induced tumour and background non-tumour, one unpaired tumour and four additional DEN-treated background liver tissue.

### ATAC-seq data processing and analysis

ATAC-seq data processing was performed using a custom-made Snakemake pipeline (v6.1.1)^98^. The quality of raw reads was assessed using fastQC (v0.11.9)^99^ and adaptor sequences were removed using cutadapt (v2.6)^100^. Reads were aligned to their respective reference genome assemblies using bwa (v0.7.17)^60^. Duplicate reads were marked using Picard (v2.23.8)^101^.

Alignment filtering was performed with samtools (v1.9)^70^ and reads overlapping genomic regions with abnormal read coverage (previously described) were removed. Reads aligning to mitochondrial DNA were excluded from further analysis. Read positions aligning to plus and minus strands were offset by +4 bp and −5 bp, as previously described^102^.

Peaks were called using MACS2 (ref. ^94^) from all fragments, where ‘fragment’ refers to the inset between 5′ ends of read 1 and read 2, and sub-nucleosomal size fragments (less than 100 bp). This was done for each sample separately and for a pool containing all replicates per condition.

The irreproducible discovery rate (IDR) method^103^ was used to determine a set of reproducible peaks. IDR was performed pairwise on replicates, using a subset of pooled peaks as reference. This subset contained pooled peaks that overlap at least 25% of the peak length in both replicates compared. Pooled replicate peaks that passed the IDR threshold of 0.05 in at least one pairwise comparison were deemed reproducible.

ATAC-seq data (FASTQ files and peak calls) are available in ArrayExpress at EMBL-EBI under accession E-MTAB-11780 (ref. ^28^) and E-MTAB-14144 (this study).

### Statistical analyses

Statistical analyses were performed in R (v4.3.2)^104^. Mann–Whitney tests were performed using the wilcox.test function as two-sided tests. Student’s *t*-tests were conducted using the t.test function with default settings of conducting two-sided, non-paired tests and not assuming equality of variance. Kolmogorov–Smirnov tests were carried out with the ks.test function as two-sided tests. Pearson’s correlations were calculated using the cor.test function. Two-sided Fisher’s exact tests were calculated using the fisher.test function. Whenever multiple tests were performed, such as multiple pairwise comparisons, Bonferroni correction for multiple testing was applied.

Linear mixed-effects models with continuous response variables were defined and fitted with maximum likelihood using the lmer function from the lme4 package (v1.1-37)^105^. Random intercept full models were defined as response ~ 1 + (1 | strain/litter/animal), where response was a continuous variable such as base substitution mutation rate or latent factor enrichment of the expression independent component, and the factors strain, litter and animal modelled as random effects. Nested models were defined as the full model with specific random effects variables excluded. Likelihood ratio tests were conducted using the lme4 package called through the generic anova function to compare the fit of nested models. For example, to test the significance of strain as an independent contributor to the fit of the full model, the fit of the full model was compared (with the likelihood ratio test using the *χ*^2^ distribution) to the fit of a model that excludes strain as a random effects variable. For binary response variables such as driver mutation presence or absence, the same procedure was used, but using the lme4 function glmer and a binomial model.

To test for associations between expression signatures and measures of selection, the latent factor enrichment of the expression independent component was the response variable, strain was fit as a random effects variable and the selection measure was fit as a binary fixed effect. The likelihood ratio test considered a comparison of models that did and did not include the selection measure (Extended Data Fig. 10f). Tests for the association of categorical tumour features including driver gene, multiple drivers (binary yes or no) and aneuploidy (binary yes or no), these features were treated as a response variable using the glmer function with a binomial model, the selection measures as fixed effects and strain as a random effect (Extended Data Fig. 10g). Testing the significance of strain–driver mutational interactions (Fig. 4f) used the lmer function and likelihood ratio test to compare nested models that differed in a MAPK driver × strain interaction defined as: response ~ 1 + MAPK + strain + MAPK:strain + (1 | motherId/animalId) versus response ~ 1 + MAPK + strain + (1 | motherId/animalId), where the response variable was the latent factor enrichment of the expression independent component and MAPK the categorial identity of the MAPK pathway driver mutation assigned with the priority *Hras* > *Egfr* > *Braf* in the case of multiple drivers. Samples without driver mutations in these genes were excluded. All tests were Bonferroni corrected for multiple testing.

Power analysis for the mixed-effects models was conducted by simulation, preserving the hierarchical structure of the study data (581 tumours nested within 215 animals, 110 litters and 4 strains). For continuous response variables, we generated data by random sampling from a normal distribution with mean 0 and standard deviation equal to the square root of the specified variance. The variance was set to zero to represent no effect. For each simulated tumour, the response was defined as the sum of the simulated random effects at each level of the hierarchy and the simulated residual component. The same general procedure was used for power analysis of categorical response variables, but in this case, the summed effects were converted to a probability vector through inverse logit and used as the basis for sampling from the binomial distribution.

We examined scenarios with ‘pure’ animal, litter or strain effects, in which only one level contributed to between-group variance at a time. For these scenarios, effect sizes ranged from 0% to 68% of the total variance (for example, 68% of the variance attributable to differences between litters, with none attributable to strain or animal). For each effect size at each hierarchical level, 1,000 simulated datasets were generated and fitted using the corresponding nested mixed-effects model. Likelihood ratio tests were then used to assess the presence of the relevant random effect. For each scenario, the proportion of simulations with *P* < 0.05 was recorded as statistical power when the tested effect matched the simulated non-zero effect, and as false discovery when it did not. Non-parametric bootstrap resampling (*n* = 100) of the simulation results was used to obtain 95% confidence intervals of these estimated proportions.

### Computational analysis environment

Quality control and alignment of WGS, ChIP–seq, ATAC-seq and RNA-seq data and WGS variant calling were performed in a Linux cluster with LSF batch control. Except where otherwise noted, subsequent analysis was performed on a Linux cluster with Altair Grid Engine batch control, analysis in Conda environments and choreographed with Snakemake (v7.32.4). Data analysis and figure generation were performed in R (v4.3.2)^104^.

### Reporting summary

Further information on research design is available in the Nature Portfolio Reporting Summary linked to this article.

## Online content

Any methods, additional references, Nature Portfolio reporting summaries, source data, extended data, supplementary information, acknowledgements, peer review information; details of author contributions and competing interests; and statements of data and code availability are available at https://doi.org/10.1038/s41586-026-10821-z.

## Supplementary information

Supplementary Note Supplementary notes regarding structural variation and non-coding mutations. Complementary results about individual chromosome losses and gains, sister chromatid exchange, and mutations occurring within regulatory sites.

Reporting Summary

Supplementary Table 1 Table of sequenced tumours containing key metadata. For each sequenced tumour sample, summarising: sample identifiers, animal and parent identifiers, diagnosis and histopathological features, mutation signatures and counts, cellularity estimates, count of sister chromatid exchange events, mutation asymmetry metrics, identified driver mutations, and sequencing accession identifiers.

Supplementary Table 2 Key resources, software, and datasets. Summary of reagents, resources, datasets, and software used, including (where applicable) unique identifiers, references, and version numbers.

Supplementary Table 3 List of DEN-naive mice assessed for the presence of tumours. Metadata for spontaneous liver tumours arising in untreated mice, including age-matched controls.

Supplementary Table 4 List of reference genome mis-assemblies. Genomic coordinates and consensus support values for the mi-assemblies identified in each of the four reference genomes.

Supplementary Table 5 List of genes identified from independent component analysis. Details of independent component analysis of DEN-induced tumour transcriptomes. Includes a list of all genes contributing to any of the seventeen gene expression signatures, and the outputs of pathway enrichment analysis for each signature.

Peer Review File

## Source data

Source Data Fig. 1

Source Data Fig. 2

Source Data Fig. 3

Source Data Fig. 4

Source Data Fig. 5

## Data Availability

Raw data files for all new datasets (*n* = 29) are available from ArrayExpress, the European Nucleotide Archive (ENA) and BioImage Archive at the EMBL-EBI (Supplementary Table 2): WGS (BL6 and CAROLI) accession number PRJEB15138 from the ENA; RNA-seq accession number E-MTAB-16391 from ArrayExpress; WSI (BL6 and CAROLI) accession S-BSST384 from the BioImage Archive; ChIP–seq accession number E-MTAB-14454 from ArrayExpress; and ATAC-seq accession number E-MTAB-14144 from ArrayExpress. Previously published datasets were also analysed^25,28^: WGS (C3H and CAST) accession number PRJEB37808 from the ENA; RNA-seq (C3H and CAST, P15 only) accession number E-MTAB-8518 from Array Express; WSI (C3H and CAST) accession number S-BSST383 from the BioImage Archive; ChIP–seq (C3H CTCF) accession number E-MTAB-11959 from ArrayExpress; and ATAC-seq (C3H) accession number E-MTAB-11780 from ArrayExpress. Source data are provided with this paper.

## Code availability

The analysis pipeline including Conda and Snakemake configuration files can be obtained without restriction from the repository (https://git.ecdf.ed.ac.uk/taylor-lab/lceGeneticBackground).
