# Supplementary material for: Genetic background sets the trajectory of experimental cancer evolution
Source: Nature. 2026 Jul 22;657(8133):1035–44. doi: 10.1038/s41586-026-10821-z (PMC13600922; doi:10.1038/s41586-026-10821-z)
Supplement: Supplementary file 1 [file 41586_2026_10821_MOESM1_ESM.pdf]

nature portfolio

https://doi.org/10.1038/s41586-026-10821-z

Supplementary information

Genetic background sets the trajectory of experimental cancer evolution

In the format provided by the authors and unedited

Supplementary Note

Aneuploidy biases are conserved

The majority of DEN-induced tumours retain mutationally asymmetric, diploid genomes (**Fig. 2a-c**). Accordingly, large copy number alterations (CNAs >1Mb), including whole chromosome aneuploidies, are generally rare and most DEN-induced tumours (69%) have no detectable events (**Fig. 2d**). However, CNAs are significantly enriched in CAROLI tumours with WGD (odds ratio=8.3, $p=9.0\times10^{-7}$, two-sided Fisher's exact test, versus all other tumours; **Fig. 2d**), suggesting either increased tolerance of aneuploidies or greater genome instability, consistent with previous observations of WGD in cancer[106]. Chromosome 15 recurrently increases in copy-number (gain15) in all strains and, notably, contains the *Myc* oncogene that is frequently amplified in human cancers[107]. Additionally, there is a remarkable consistency across strains for gain or loss of specific chromosomes (**Extended Data Fig. 2d,e**), likely reflecting oncogenic selection for the dosage of component genes. For instance, gains of chromosome 1, 2, and 15 and losses of chromosomes 4 and 19 occur more frequently than expected, representing candidate secondary oncogenic drivers.

Other facets of genome integrity can be interpreted from sub-chromosomal patterns of mutational asymmetry. After identifying and filtering out artefacts from reference genome misassemblies (**Extended Data Fig. 2f-i**; **Supplementary Table 4**), transitions between genomic blocks of asymmetry reveal sites of sister-chromatid exchange (SCE) that occurred in the first cell-generation after mutagenesis[25,26]. These transitions are thought to reflect homologous repair, for example at sites of DNA double-strand breaks, with an average 27 (s.e.m.=0.02) SCEs per tumour genome, though significantly fewer are observed in CAROLI (mean=23, s.e.m.=0.2, p=0.007, Mann-Whitney U test versus all other strains, only considering mutationally asymmetric tumours).

Mutagenesis of the non-coding genome

All of the oncogenic driver events identified in our multi-species analyses involve protein coding genes. However, the vast majority of mutations in these DEN-induced tumours arise in the non-coding genome, mimicking the findings of human pan-cancer analyses[108,109] reflecting the composition of their underlying genomes[110,111]. Cell of origin influences tumourigenesis[112,113] and we have demonstrated that transcription coupled repair of genes is prevalent in the DEN system[25,27,28]. In order to examine mutation rates at functionally important sites in the non-coding genome, we profiled the epigenomic (ChIP-seq for H3K4me3, H3K27ac, and CTCF) and transcriptional (total RNA-seq) landscape of P15 livers from each strain and used biologically-reproducible peaks to identify enhancers and promoters.

We previously identified that DEN-induced tumours have a dramatically elevated mutation rate in CTCF-binding sites in C3H mice[28], and now we show this is consistent across species. While there are marked differences in the mutation rate between classes of regulatory sites (bound CTCF sites > enhancers > promoters) within each strain, the ranking is common between strains (**Extended Data Fig. 6a-d**), implying that relative repair efficiencies are conserved. Next, to assess the effect of protein-DNA binding and regulatory activity on mutation rates, we identified regions that aligned across all four strains, and exhibited CTCF binding or regulatory activity in at least one strain. Aligned orthologous regions that did not show activity in a specific strain were defined as a "shadow" region in that strain (**Extended Data Fig. 6e**). Differences in mutation rate between bound and shadow regions then reveal differences in mutation properties that can be attributed to protein binding. For CTCF, bound regions had higher mutation rates than unbound shadow regions which supports the finding that, in addition to impeding repair of transcription factor binding sites[41], CTCF binding can increase DNA

damage[28]. In contrast, the mutation rate of active enhancers and promoters was lower than inactive shadow regions, indicative of greater repair efficiency at the corresponding active, and likely accessible, enhancers and promoters (**Extended Data Fig. 6f-i**).

Additional references

106. Prasad, K. et al. Whole-genome duplication shapes the aneuploidy landscape of human cancers. *Cancer Res*. **82**, 1736–1752 (2022).
107. Dhanasekaran, R. et al. The MYC oncogene - the grand orchestrator of cancer growth and immune evasion. *Nat. Rev. Clin. Oncol*. **19**, 23–36 (2022).
108. Khurana, E. et al. Role of non-coding sequence variants in cancer. *Nat. Rev. Genet*. **17**, 93–108 (2016).
109. ICGC/TCGA Pan-Cancer Analysis of Whole Genomes Consortium. Pan-cancer analysis of whole genomes. *Nature* **578**, 82–93 (2020).
110. Nurk, S. et al. The complete sequence of a human genome. *Science* **376**, 44–53 (2022).
111. Liu, J. et al. The complete telomere-to-telomere sequence of a mouse genome. *Science* **386**, 1141–1146 (2024).
112. Hoadley, K. A. et al. Cell-of-origin patterns dominate the molecular classification of 10,000 tumors from 33 types of cancer. *Cell* **173**, 291–304.e6 (2018).
113. Panja, S. et al. Passenger mutations link cellular origin and transcriptional identity in human lung adenocarcinomas. *Nat. Genet*. **57**, 3066–3074 (2025).
