# Supplementary material for: Genetic background sets the trajectory of experimental cancer evolution
Source: Nature. 2026 Jul 22;657(8133):1035–44. doi: 10.1038/s41586-026-10821-z (PMC13600922; doi:10.1038/s41586-026-10821-z)
Supplement: Supplementary file 2 [file 41586_2026_10821_MOESM2_ESM.pdf]

nature portfolio

Corresponding author(s): Sarah Aitken, Martin Taylor, Duncan Odom

Last updated by author(s): May 21, 2026

Reporting Summary

Nature Portfolio wishes to improve the reproducibility of the work that we publish. This form provides structure for consistency and transparency in reporting. For further information on Nature Portfolio policies, see our Editorial Policies and the Editorial Policy Checklist.

Statistics

For all statistical analyses, confirm that the following items are present in the figure legend, table legend, main text, or Methods section.

n/a	Confirmed	
☐	☒	The exact sample size (*n*) for each experimental group/condition, given as a discrete number and unit of measurement
☐	☒	A statement on whether measurements were taken from distinct samples or whether the same sample was measured repeatedly
☐	☒	The statistical test(s) used AND whether they are one- or two-sided<br>*Only common tests should be described solely by name; describe more complex techniques in the Methods section.*
☐	☒	A description of all covariates tested
☐	☒	A description of any assumptions or corrections, such as tests of normality and adjustment for multiple comparisons
☐	☒	A full description of the statistical parameters including central tendency (e.g. means) or other basic estimates (e.g. regression coefficient) AND variation (e.g. standard deviation) or associated estimates of uncertainty (e.g. confidence intervals)
☐	☒	For null hypothesis testing, the test statistic (e.g. *F*, *t*, *r*) with confidence intervals, effect sizes, degrees of freedom and *P* value noted<br>*Give P values as exact values whenever suitable.*
☒	☐	For Bayesian analysis, information on the choice of priors and Markov chain Monte Carlo settings
☐	☒	For hierarchical and complex designs, identification of the appropriate level for tests and full reporting of outcomes
☐	☒	Estimates of effect sizes (e.g. Cohen's *d*, Pearson's *r*), indicating how they were calculated

Our web collection on statistics for biologists contains articles on many of the points above.

Software and code

Policy information about availability of computer code

Data collection

Illumina Software Control (ICS) v.3.3.76

Data analysis

bwa mem 0.7.12
bwa mem 0.7.17
CNVkit 0.9.6
iScan Control Software (ICS) 3.3.76
kalisto 0.43.1
strelka2 2.8.4
Bedtools 2.30.0
bgsignature 0.2
BIODICA 0.9
Blast 2.5.0
clusterProfiler 4.14.0
CNVkit 0.9.6
cutadapt 2.6
DESeq2
dNdScv 0.1.0
fastQC 0.11.9
GeneWise 3.cee4437
HAL toolkit 2.3
lme4 1.1-37

April 2023

MACS 2.1.2
MHCflurry 2.1.1
netMHCpan 4.1
OncodriveCLUSTL 1.1.13
OncodriveFML 2.2.0
PHAST phyloFit 1.3
Picard 2.23.8
progressiveCactus 1.0.0
pybedtools 0.8.0
QuPath 0.2.2
R 4.3.2
r-GreyListChIP 1.36.0
r-nmf 0.28
RepeatMasker 20170127
samtools 1.9
SELECT 1.6
SIFT 83
SigProfiler 1.2.19
SnakeMake 6.1.1
SnakeMake 7.32.4
StarDist
Strelka2 2.8.4
Telomerecat 4.0.1
tximport 0
VEP (Variant Effect Predictor) 109.3

For manuscripts utilizing custom algorithms or software that are central to the research but not yet described in published literature, software must be made available to editors and reviewers. We strongly encourage code deposition in a community repository (e.g. GitHub). See the Nature Portfolio guidelines for submitting code & software for further information.

Data

Policy information about availability of data

All manuscripts must include a data availability statement. This statement should provide the following information, where applicable:

- Accession codes, unique identifiers, or web links for publicly available datasets
- A description of any restrictions on data availability
- For clinical datasets or third party data, please ensure that the statement adheres to our policy

Raw data files for all new datasets are available from ArrayExpress, the European Nucleotide Archive (ENA), and BioImage Archive at the EMBL-EBI. WGS (BL6 and CAROLI) accession number from ENA: PRJEB15138. RNA-seq accession number from ArrayExpress: E-MTAB-16391. WSI (BL6 and CAROLI) accession from BioImage Archive: S-BSST384. ChIP-seq accession number from ArrayExpress: E-MTAB-14454. ATAC-seq accession number from ArrayExpress: E-MTAB-14144.

Previously published datasets were also analysed (Aitken et al. 2020; Anderson et al. 2024): WGS (C3H and CAST) accession number from ENA: PRJEB37808. RNA-seq (C3H and CAST, P15 only) accession number from Array Express: E-MTAB-8518. WSI accession from BioImage Archive (C3H and CAST): S-BSST383. ChIP-seq (C3H CTCF) accession number from ArrayExpress: E-MTAB-11959. ATAC-seq (C3H) accession number from ArrayExpress: E-MTAB-11780.

Research involving human participants, their data, or biological material

Policy information about studies with human participants or human data. See also policy information about sex, gender (identity/presentation), and sexual orientation and race, ethnicity and racism.

Reporting on sex and gender	Not applicable.
Reporting on race, ethnicity, or other socially relevant groupings	Not applicable.
Population characteristics	Not applicable.
Recruitment	Not applicable.
Ethics oversight	Not applicable.

Note that full information on the approval of the study protocol must also be provided in the manuscript.

Field-specific reporting

Please select the one below that is the best fit for your research. If you are not sure, read the appropriate sections before making your selection.

☒ Life sciences ☐ Behavioural & social sciences ☐ Ecological, evolutionary & environmental sciences

For a reference copy of the document with all sections, see nature.com/documents/nr-reporting-summary-flat.pdf

Life sciences study design

All studies must disclose on these points even when the disclosure is negative.

Sample size	Sample sizes for the main DEN induced oncogenesis study were chosen to be the maximum possible with available resource. DEN induced tumours from C3H/HeOuJ and CAST/EiJ were previously whole genome sequenced and reported. Tumours from C57BL/6J and CAROLI/EiJ were collected with the aim to approximately match sample size with the smaller CAST/EiJ cohort for balanced comparisons between strains.
Data exclusions	Except where otherwise stated in relevant results sections, analyses were confined to n=581 clonally distinct DEN-induced dysplastic nodules. When multiple samples of a given tumour were available, only one was included; this is based on the previous finding that the majority of tumours are highly clonal (Aitken et al., Nature 2020). Mutation spectrum analysis included additional normal liver samples, spontaneous liver tumours, and hepatocellular carcinomas (HCCs) for comparison.
Replication	The study design was based on replication and comparison between four distinct, inbred genetic backgrounds (strains). All analyses were conducted separately for each genetic background and conclusions drawn based on the replication/consistency of findings or not. Within a strain or defined cohort the replication and robustness of findings was tested both by within strain replication (e.g. minimum of n=55 DEN induced tumours per strain) and bootstrap sampling of cohorts (e.g. driver mutation rate analysis) or permutation of category assignments (e.g. recurrent aneuploidy analysis).
Randomization	This study did not involve a randomised case:control study design. Randomisation was used to permute genes and tumour classifications for permutation based analysis (using the R sample function without replacement). Randomisation was used for bootstrap analyses (R sample function with replacement). Analysis code provided uses a defined seed for random number generation for reproducibility but equivalent results in each case were obtained using pseudo-randomly generated seeds.
Blinding	Researchers were blinded during histological assessment of tumours. Blinding was not relevant for genomic analyses as all processing was automated and all samples/genomic-loci meeting pre-defined inclusion criteria were processed.

Reporting for specific materials, systems and methods

We require information from authors about some types of materials, experimental systems and methods used in many studies. Here, indicate whether each material, system or method listed is relevant to your study. If you are not sure if a list item applies to your research, read the appropriate section before selecting a response.

Materials & experimental systems

n/a	Involved in the study	
☐	☒	Antibodies
☒	☐	Eukaryotic cell lines
☒	☐	Palaeontology and archaeology
☐	☒	Animals and other organisms
☒	☐	Clinical data
☒	☐	Dual use research of concern
☒	☐	Plants

Methods

n/a	Involved in the study	
☐	☒	ChIP-seq
☒	☐	Flow cytometry
☒	☐	MRI-based neuroimaging

Antibodies

Antibodies used	CTCF antibody (rabbit polyclonal, Merck Millipore 07-729, lot 2517762).<br>H3K4me3 (mouse monoclonal IgG clone CMA304, Merck Millipore 05-1339, lot 2780484).<br>H3K27ac (rabbit polyclonal IgG, Abcam 4729, lot GR3187598).
Validation	CTCF: Millipore https://www.emdmillipore.com/US/en/product/Anti-CTCF-Antibody,MM_NF-07-729?bd=1<br>Routinely evaluated by Western Blot.<br>Western Blot Analysis: 1:1000–1:5000 dilution of this lot detected CTCF in HeLa nuclear extract. A previous lot detected CTCF in K562 nuclear extract.<br>Application - Use Anti-CTCF Antibody (Rabbit Polyclonal Antibody) validated in ChIP, WB, ChIP-seq to detect CTCF also known as 11-zinc finger protein, CCCTC-binding factor.<br>The same antibody has also been used and validated in previous publications, for example https://doi.org/10.1186/s13059-018-1484-3<br><br>H3K4me3: Millipore https://www.emdmillipore.com/US/en/product/Anti-Trimethyl-Histone-H3-Lys4-Antibody-clone-CMA304,MM_NF-05-1339<br>Western Blot Analysis: 1:500-2000 dilution of this antibody detected trimethyl Histone H3 (Lys4) in HeLa acid extract.<br>Application - Anti-Trimethyl Histone H3 (Lys4) Antibody, clone CMA304 is a mouse monoclonal antibody for detection of Trimethyl Histone H3 (Lys4) also known as H3K4me3, Histone H3 (tri methyl K4) & has been validated in ELISA, ICC, IP, WB, Mplex, DB.<br>The same antibody has been used and validated in previous publications, for example: https://doi.org/10.1016/j.cell.2015.01.006<br><br>H3K27ac: Abcam https://www.abcam.com/en-us/products/primary-antibodies/histone-h3-acetyl-k27-antibody-chip-grade-ab4729<br>Validation data shown available for ChIP (2 µg antibody), Western blot (1 µg/ml) , Immunohistochemistry (5 µg/ml), and

Immunofluorescence (1µg/ml) in HeLa cells.
The same antibody has been used and validated in previous publications, for example: https://doi.org/10.1016/j.cell.2015.01.006

Animals and other research organisms

Policy information about studies involving animals; ARRIVE guidelines recommended for reporting animal research, and Sex and Gender in Research

Laboratory animals	15-day-old (P15) male mice of strains C3H/HeOuJ, C57BL/6J, CAST/EiJ and CAROLI/EiJ were treated with a single intraperitoneal (IP) injection of N-Nitrosodiethylamine (DEN; Sigma-Aldrich N0258; 20 mg/kg body weight) diluted in 0.85% saline. Liver tumour samples were collected from DEN- treated mice 25 weeks (C3H/HeOuJ), 36 weeks (C57BL/6J), 38 weeks (CAST/EiJ) or 78 weeks (CAROLI/EiJ) after treatment. Additional tissues from untreated P15 mice (ear, tail, and liver) and untreated age matched adult mice (liver only; C3H/HeOuJ 27 weeks, C57BL/6J 38 weeks, CAST/EiJ 40 weeks, CAROLI/EiJ 80 weeks) were sampled for control experiments. In addition, a cohort of untreated DEN naive mice from each strain were aged to assess the incidence and latency of spontaneous tumours.
Wild animals	This study did not involve wild animals.
Reporting on sex	This study was based on male mice. It has previously been established that male mice are more susceptible to DEN induced tumour induction and more reliably develop tumours. Consequently limiting the study to male mice minimised the number of animals required. Further - prior data on 371 C3H/HeOuJ and 84 CAST/EiJ DEN induced tumours was generated only from male mice for the same reasons, so for fair comparison to this previously published data it was appropriate to use all male cohorts for the additional strains.
Field-collected samples	The study did not use samples collected from the field.
Ethics oversight	Animal experimentation was carried out in accordance with the Animals (Scientific Procedures) Act 1986 (United Kingdom) and with the approval of the Cancer Research UK Cambridge Institute Animal Welfare and Ethical Review Body (AWERB): the maximum approved tumour burden was 10% body weight, which was not exceeded.

Note that full information on the approval of the study protocol must also be provided in the manuscript.

Plants

Seed stocks	Not applicable.
Novel plant genotypes	Not applicable.
Authentication	Not applicable.

ChIP-seq

Data deposition

☒ Confirm that both raw and final processed data have been deposited in a public database such as GEO.

☒ Confirm that you have deposited or provided access to graph files (e.g. BED files) for the called peaks.

Data access links<br>*May remain private before publication.*	ArrayExpress accession: E-MTAB-14454<br>https://www.ebi.ac.uk/biostudies/arrayexpress/studies/E-MTAB-14454?key=62700e34-c0ce-4005-bf40-ff9c30a3a0b6
Files in database submission	*Provide a list of all files available in the database submission.*
Genome browser session<br>(e.g. UCSC)	*Provide a link to an anonymized genome browser session for "Initial submission" and "Revised version" documents only, to enable peer review. Write "no longer applicable" for "Final submission" documents.*

Methodology

Replicates	All experiments were performed with three to five biological replicates, with matched input controls. Each biological replicate comprised pooled liver tissue from ten individual mice.
Sequencing depth	Pooled libraries were sequenced on a HiSeq4000 (Illumina) to produce paired-end 150 bp reads.<br>There were an average of 43,541,260 reads per library, of which 36,809,656 were uniquely mapping.
Antibodies	CTCF antibody (rabbit polyclonal, Merck Millipore 07-729, lot 2517762).

Antibodies	H3K4me3 (mouse monoclonal IgG clone CMA304, Merck Millipore 05-1339, lot 2780484).<br>H3K27ac (rabbit polyclonal IgG, Abcam 4729, lot GR3187598).
Peak calling parameters	To identify ChIP-seq positive regions, sequencing reads were trimmed to 50 bp and then aligned to their respective genomes using bwa (v0.7.17) using default parameters. Peaks were identified for each ChIP library using MACS (v.2.1.2) and matched input controls; for histone modifications, the "--broad" flag was used. Peaks with q-value <0.05 were included in downstream analyses. Input libraries were used to filter spurious peaks associated with a high input signal using the GreyListChIP R package (v.1.36.0).
Data quality	Biologically-reproducible peaks were identified by merging ChIP-seq peaks defined as above (with an FDR q-value <0.05) from individual replicates and selecting those that overlapped ≥2 individual replicate peaks.
Software	MACS2 (v.2.1.2) callpeak with default parameters for CTCF and the "--broad" flag for histone modifications.
