# Supplementary material for: Genetic background sets the trajectory of experimental cancer evolution
Source: Nature. 2026 Jul 22;657(8133):1035–44. doi: 10.1038/s41586-026-10821-z (PMC13600922; doi:10.1038/s41586-026-10821-z)
Supplement: Supplementary file 3 [file 41586_2026_10821_MOESM8_ESM.pdf]

nature portfolio

Peer Review File

Genetic background sets the trajectory of experimental cancer evolution

Corresponding Author: Dr Sarah Aitken

This file contains all reviewer reports in order by version, followed by all author rebuttals in order by version.

Version 0:

Reviewer comments:

Referee #1

(Remarks to the Author)
This work analyzes dependencies of cancer evolution on genetic background. The authors use their well-established model of DEN-induced liver cancer in mice. All four strains included in the comparison stably develop tumors following a pulse of DEN mutagenesis but differ in length of the latency period. All four strains develop driver mutations in the MAPK pathway, show similar expression changes of the MAPK pathway gene transcripts and feature highly concordant copy number variants (both gains and losses). At the same time, presence of WGD, number of drivers, usage of driver genes and specific driver mutations all depend on genetic background. Some of these observations are evident from the earlier work by the same authors, but this manuscript analyzes additional strains and puts the genetic background dependency in focus. Overall, this is an important topic, and I found the paper of great interest. At the same time, the analysis and presentation can be greatly improved. Also, the manuscript offers very little mechanistic insight.

1) The manuscript presents the analysis of 581 tumors. If I am not mistaken, the earlier publication analyzed over 350 tumors. It would be helpful to know how many data points are new.
2) The presentation of results would benefit from displaying the differences between strains normalizing to inter-individual variation within strains (ideally also showing difference in littermates vs. unrelated mice). For example, one can estimate the proportion of variance explained by genetic background.
3) The analysis of gene expression is currently limited to the presentation of first two principal components highlighting major sources of expression variance among the samples. It is not clear whether the effects of tumorigenesis on gene expression differ due to genetic background (the main topic of the paper).
4) Is it possible to expand the analysis of genetic differences within driver genes beyond the window of total conservation? Does the number of amino acid substitutions track differences in usage of individual driver mutations? Is the structural neighborhood more informative than the sequence neighborhood?
5) The story of the strand bias of HRAS Q61 mutations is fascinating. Some Q61L tumors are symmetric. Does this affect the inference of the strand bias? Also, the idea of “the production of pro-oncogenic miscoded proteins before replication” is confusing. Do the authors imply that Q61L and Q61R are driven by different lesion types that result in different transcripts over the lesion-containing template strand? Is there any evidence of transcription over a DEN-induced lesion?
6) The finding of differences in distributions of times to MRCA between strains is of clear interest. However, additional details would be welcome here. Is it possible to analyze these differences separately for different drivers (to control for the driver-specific fitness advantage)? Also, is it possible to analyze the difference between tumors with one known driver vs. two known drivers (I understand that the former will be dominated by C3H tumors)?
7) The “dispersion test” is creative. Does it have sufficient power to detect partial loss of lineages? It Is not immediately obvious that the quantitative effect on dispersion would be strong enough. Is it possible that the analysis is confounded by differences in somatic recombination rates? Unless it is too demanding, computer simulations may be helpful here.

Referee #2

(Remarks to the Author)
This study compares the genomic and transcriptomic features of mutagen-induced (DEN) liver tumours between four mouse strains. Tumours in all strains show convergent activation of MAPK signalling, then the paper reports inter-strain differences in other molecular features: driver mutations, genome doubling, subclonal selection. The authors conclude that germline variation strongly determines the course of tumour evolution.

The authors' liver cancer consortium has generated an incredible resource, that this paper is further evidence of the value of. There is a lot of data in this manuscript, and the authors do a very good job at constructing an easy-to-follow narrative from it, clearly explaining key features in the data.

The paper is very convincing that inter-strain differences in tumour evolution exist - but I'm not convinced this is a novel finding in general (explained below).

I think a major limitation of the manuscript is that it provides very little mechanistic insight into *why* the inter-strain differences exist. I really wanted to see a specific consideration of precisely what the germline variation between species is, what its function is, and how this causes differences in tumour evolution. At present this is not explored at all.

The final sentence of the introductory paragraph says "it is not clear how consistently cancer would develop given the same genetic background and same environment, or how germline differences influence the trajectory to tumourigenesis". I think this point is oversold: there is already a big literature on this exact question.

Specifically:

There is a large literature on strain dependence in mouse models, across cancer types (perhaps most famously the mom locus in apc/min models of intestinal cancer: https://www.sciencedirect.com/science/article/pii/0092867493904848)
There is also literature on how mouse germline genetics alters tumour biology, for instance this manuscript describing metastases suppression by germline variants: https://www.nature.com/articles/nature20792

The molecular analysis in this new manuscript is more comprehensive that anything I have seen before, but arguably only incrementally so.

In humans, there are very clear examples of how genetic background determines tumour evolution in patients: take tumours arising in Lynch, FAP, VHL or even BRCA carriers for example (compared to sporadic tumour counterparts).

Further, also in human, there are previous detailed investigations of the molecular/evolutionary differences between the multiple tumours that arise synchronously in these high-penetrance predisposition syndromes (i.e. human tumours with an identical genetic background, and essentially identical microenvironment),
such as in FAP in colon: https://www.nature.com/articles/s43018-024-00831-z (and multiple other papers)
VHL carriers in kidney: https://genomebiology.biomedcentral.com/articles/10.1186/s13059-014-0433-z and a recent bigger study that is presumably in review? https://aacrjournals.org/cancerres/article/83/7_Supplement/133/721425/Abstract-133-Rewinding-the-tape-of-life-somatic
BRCA carriers with bilateral breast cancer:
https://www.nature.com/articles/s41591-023-02216-8

These studies collectively show that quite big differences in tumour evolution occur between tumours arising from essentially-identical starting points.

There is also a big literature on how human genetic ancestry (ethnicity) influences cancer biology. Perhaps prostate cancer is most instructive, where the somatic genetics of prostate cancers arising in black vs white men show stark differences, tumour biology is different, and there is correlated, probably explanatory, germline variation in the androgen receptor.

Specific comments:
1. Fig 1A reports differences in tumour latency between strains, that appear to correlate with the evolutionary divergence between strains. I would really like to learn why from this manuscript!

2. Fig 2A reports that the mutational consequence of DEN exposure varies between strains, with the DEN2 signature enriched in C3H strain. The authors should investigate why this is. There is a comment about the role of MGMT expression but no data are shown. I think the authors have an opportunity to explore their RNA data, germline variants and chromatin state of the MGMT pathway, and/or other DNA repair pathways, etc.

3. The analysis of mutation burden feels quite limited. I think there is a missed opportunity to explore the distribution of mutations around the genome, and correlations with epigenetic state (same for driver analysis). There is some of this analysis in fig5 and figS6 but it is limited to a global analysis of promoters, enhancers and CTCF sites. Perhaps this genome-wide heterogeneity in mutation susceptibility mechanistically explains some of the inter-strain variability in burden.

4. The frequent whole genome doubling (WGD) in the CAROLI strain is interesting; also that this mouse has more frequent and distinct CNAs. Again, there is no exploration of what might cause these differences. What are the germline variants in CNA regions that are divergent between lines? Are these explanatory? There may even be "pathogenic" germline variants in these regions.

5. Are there clues from the normal liver data about the baseline level of MAPK signalling or otherwise about why there are driver identity and number differences between strains? Are there explanatory germline variants? Are strain-biased drivers in differential chromatin state regions?

6. Fig 5 (and associated sup figures) begins to explore background gene expression and chromatin differences between strains. It would be interesting if these data were more directly related to germline genetic differences between strains.

7. Fig 6 – I think this is the most interesting part of the paper (though ironically is less about inter-strain differences). I really like the analysis of mutations on template vs non-template strand. The analysis suggests that many driver mutations arise in cells that are not actively transcribing the oncogenic driver (because the lesions are not repaired by TCR), and therefore suggests inter-tumour differences in the cell of origin of tumours. Can the authors see this reflected in the genome-wide transcriptional phenotype of tumours? Can they see it in their digital pathology data? Alternatively, is there any evidence of inter-allele heterogeneity in expression? (could it be that expression “bursts” from one allele or other, that mean that TCR is biased to one allele or other?) Again a deeper analysis that provides more molecular mechanism would be great to see.

8. Fig 7 – subclonal selection. The data on multiallelism show that “instant transformation” is most common in CH6 tumours: this is interesting. Could the authors explore how the retained mutagenized allele relates to driver genes (as they did in their original Lesion Segregation paper; fig2h), and possibly identify subclonal drivers this way? How is the analysis in Fig 7b affected by CNAs (WGD is already excluded I think). Please check that VAF/CCF distributions are consistent with the multiallelism data: VAF/CCF distributions should show a characteristic peak of subclonal mutations in tumours where subclonal selection is inferred. The identity of mutations in the subclonal peak can then be assessed to identify drivers.

9. Are there any distinctive molecular features of tumours with subclonal selection? Does tumour/subclonal identity correlate with strain identity and is there a potential mechanism?

10. In general, it would be good to include to include more specific (quantitative) comparisons of within-strain vs between-strain heterogeneity. I appreciate some of this is already in the paper, but usually the comparison is only implicit (e.g. driver mutation burden fig 4d).

Minor:
11. Please add statistical tests about the differences in tumour latency between strains (fig 1A)
12. Please add statistical tests about mutation burden variation between strains (Fig 2B)
13. Please add statistics on inferred frequency of genome doubling, associations with nuclear size, etc throughout Fig 3.

Referee #3

(Remarks to the Author)
This original study, using a well-designed one-time DEN exposure in four mouse models with distinct varying germline genetic backgrounds, aims to advance our understanding of how germline genetic and environmental factors interact in tumor initiation and progression. As someone outside the in vivo research field, I find this particularly exciting, as such insights are difficult to achieve through epidemiological studies. However, while in vivo models offer unique advantages, caution is necessary when extrapolating and translating findings to human settings.

The introduction and discussion are well-structured and accessible to a wider public. However, the result section is more challenging to follow, despite its logical and stepwise presentation, with each section focusing on one part of the analyses. While a narrative is clearly present, the wider public may need a bit more guidance. Does the observed lack of differences in onco-genetic profiles between the strains suggest that the selected in vivo models are more comparable to each other and/or that other (unmeasured) factors play a larger role than expected? This is also relevant in the light of human population differences observed after specific exposures (e.g. https://pubmed.ncbi.nlm.nih.gov/38693263/). Additionally, for example, in several instances CAROLI is the significant onco-genetic profile outlier though this seems unrelated to the longer tumor latency. Given that tumor latency is likely a characteristic of the specific genetic background, further clarification on how these observations fit into the broader narrative would be beneficial.

Further specific comments on the results section:

Mostly the estimates are well-summarized in the text, which is important since these are often quite hard to read/extract from the figures. However, lines 76–85 require additional support for the reported differences in tumor latency (both DEN-induced and spontaneous), including mean ± SD values and statistical tests.

Given the genetic differences between the strains, how representative are the findings regarding the relative contributions of development, DEN exposure, and oncogenesis? Specifically, can conclusions drawn from transcriptomic data in DEN-exposed BL6 background liver (distant to tumors) be extended to the other strains (and beyond)?

Some specific conceptual considerations:

The study’s proof-of-principle is exciting. However, caution is warranted when generalizing its findings beyond this specific DEN exposure and liver cancer model. The conclusion in the abstract—stating that "These results show that interactions between the germline genome and the environment are highly deterministic for the trajectory of tumour genome evolution, and even modest genetic divergence can substantially alter selection pressures during cancer development, influencing both cancer risk and the biology of the tumour that develops." —may be overstated.

Epidemiological research indicates that genetic background and specific exposures influence cancer types and mutational

profiles in well-documented ways. For example, BRCA1 pathogenic variants are strongly associated with triple-negative breast cancer (e.g. https://pubmed.ncbi.nlm.nih.gov/35084436/), while smoking is linked to specific lung cancer subtypes (e.g. https://pubmed.ncbi.nlm.nih.gov/38203395/). Admittedly, unfortunately (large-scale) studies on germline-exposure interactions remain limited and inconclusive, particularly regarding tumor genetic profiles rather than just cancer risk, largely due to sample size constraints (lack of power).

Furthermore, epidemiological and clinical studies have established that the impact on tumor subtypes of germline mutations depends on the mutation's pathogenicity or penetrance (even within for example high risk BRCA mutation carriers; e.g. https://pubmed.ncbi.nlm.nih.gov/25849179/). Similarly, environmental exposures have varying effects strenghts —while smoking has a strong association with lung cancer (https://pubmed.ncbi.nlm.nih.gov/12517539/), other factors, such as alcohol, have weaker relative effects on e.g. breast cancer risk (https://pubmed.ncbi.nlm.nih.gov/39581746/).

Thus, while this study provides valuable insights, its findings do not fully support broad claims about "nature's trajectory of cancer evolution." Instead, they offer strong evidence for a specific trajectory of liver cancer under a defined exposure condition. Extending these findings to all tumor types and exposures would be speculative.

Moreover, the study's one-time high-dose exposure differs substantially from typical human exposures, which are often cumulative over a lifetime (e.g., smoking, hormones, and alcohol; e.g. https://pubmed.ncbi.nlm.nih.gov/39581746/) or repetitive due to occupational hazards (e.g., asbestos; https://pubmed.ncbi.nlm.nih.gov/39003938/). Interestingly, even in the highest risk groups there seems to be not much evidence for interaction between germline background and exposures that are known to be associated with cancer development (e.g. https://pubmed.ncbi.nlm.nih.gov/37095519/).

This raises the question whether a one-time high exposure induces a different oncogenic process compared to the durations and frequency of exposure more similar to the human situation. It While it is outside my expertise to suggest whether in vivo models could be exposed to such trajectories, at least this translational gap could be addressed in the discussion.

The reporting of the data and methodology is sufficiently detailed and individual data have been provided in the supplemental tables.

Minor clarifications:
Fig 1 a: "post partum", the method section indicates the all mice are males, should be post natal?

The data set (excel 503049_0_data_set_4644226_spvq4d) includes one female? There are also cases of ear (or tail) instead of liver?

Version 1:

Reviewer comments:

Referee #1

(Remarks to the Author)
This revision is a substantial improvement. The nested animal/litter/strain model has made the data interpretation transparent. Application of ICA, and the discussion of the differential immune response to Hras Q61 mutations in BL6 have added a lot of value. I have three minor/optional comments below.

1) It is great to see that the authors tested their nested model in simulations. A more traditional presentation of null simulations in the form of a QQ plot and also traditional power simulations would be more convincing to a reader. The authors may decide to include simulations figures with the Extended Data. Of course, if this is difficult to do, and the authors are convinced that Type I error is well controlled, this is optional.

2) I do not have any specific comments on presentation but find the manuscript difficult to read. I understand that this is a story about multiple observations but wonder if it can be structured better. Again, if difficult, it is fully optional.

3) The manuscript (especially the discussion section) overstates the relevance of the results to human cancers. Genetic diversity in the human population is sharply different from variation amongst lab mouse strains. There is no evidence that the results are relevant to epidemiological differences between human continental populations. I do not think. this makes the manuscript of a lesser importance, because the demonstration of epistatic effects on tumorigenesis is important on its own right (I understand that some readers may only care about relevance to human cancers).

Referee #2

(Remarks to the Author)
I continue to think that this is an interesting and high-quality paper, based on an excellent dataset with rigorous and innovative analysis. It reports strong evidence of differences in cancer evolutionary dynamics between mouse strains. The new hierarchical statistical analysis, bolstered by other specific analyses, very convincingly shows that these evolutionary differences are due to germline variation. The dissection of the evolutionary features (such as whole genome doubling, causes and timing of driver mutations, and subclonal selection) is careful, elegant and insightful.

I think my “worry” with the paper continues to be that it isn’t at all surprising that germline variation causes differences in tumour evolution. The literature I cited before – from humans and mice - clearly shows this. The analysis of multiple tumours from humans with high-risk (Mendelian) predisposition syndromes also shows that there is stochasticity in the evolutionary dynamics within the same genetic background, and within the same individual/organ.

The authors argue, quite convincingly, that it is not realistic to find causal (polygenic) genetic variants that drive tumour evolutionary differences, given their study design. I accept this, and appreciate that the study I cited before from van der Wyeden (Nature 2017)- identifying host genetics that altered metastasis - has a very different design. Nevertheless I do still think that not going after *why* the germline causes differences exist between strains does limit the impact of the study essentially to an over-arching result which won’t be seen as a surprise.

Nevertheless, I want to emphasise the dissection of evolutionary dynamics in the paper is elegant and innovative – particularly around timings and mechanisms of drivers and WGD – there is significant value in these data/analyses alone.

Specific replies:
a. The inclusion of the hierarchical statistical modelling is very welcome. It does convincingly show that strain (genetic background) drives the differences between mouse strains.
b. The new correlations between gene expression phenotypes and driver frequency (Fig 6) are also welcome, they hint at some possible molecular mechanisms that drive the evolutionary differences between species.
c. I appreciate the new clarity of findings 3-8 as listed in the rebuttal.

1. I agree that the authors had definitely shown (and now do even more convincingly) that inter-strain variation is due to germline genetics. I still would like to know *what* genetics is the cause, but the authors argue strongly this is not achievable, I can (sadly) accept this is the case.
2. Good that the MGMT expression levels has now been investigated and is lower in CH3 and CAST as predicted.
3. OK – I agree with the argument made here.
4. Again I can accept its not reasonable to expect to find germline variants that explain CNA biases. I find the argument that there are many SNPs between strains (too many to explore!) a little weak though; it should be possible, for example, to test if there are coding variants in “important” genes located in each CNA (especially if those genes have altered expression between strains). I can imagine variants in regulatory elements of “important” genes could also be worth looking at (a nice human example for the older literature is https://www.nature.com/articles/ng.406 relating to a MYC regulating variant identified by GWAS), though I do appreciate that this analysis is more speculative.
5. OK
6. OK
7. The reply is strong – I still find this mechanistic insight into transcription (or indeed lack of) of the HRAS is a fascinating part of the paper.
8. Subclonal selection analyses. I appreciate the involved new analysis of subclonal multiallelic variation. This section is very interesting and strong. The new overdispersion analysis concludes that subclonal selection is quite common. I think the authors should check what happens to this test if the death rate of the (transformed) cells is very high: this would cause (neutral) bottlenecking, and I think this could appear to be subclonal selection by the dispersion test. I do suspect the death rate following mutagen exposure is very high.
9. OK
10. The new likelihood ratio tests are great.

Minor: all fine (thanks)

Referee #3

(Remarks to the Author)
This manuscript has been substantially revised. The revisions have strengthened the manuscript and clarified (seeming) inconsistencies. I also highly appreciated the considerations and clarifications provided in the rebuttal and have no further questions or suggestions to offer based on my expertise.

NB apologies I made a typo and had meant to capture an essence/paraphrase ('nature’s trajectory of cancer evolution') in my comment

Version 2:

Reviewer comments:

Referee #1

(Remarks to the Author)
This revision has addressed all of my remaining comments.

Referee #2

(Remarks to the Author)
Thanks for looking at the effect of cell death in the dispersion test - the new tempering of the results I think is appropriate.

I have no further technical comments.

I appreciate the rigour with which the authors have dissected tumour evolutionary dynamics; it is high-quality work.

I wonder if changing the title to something like "Tumour evolutionary dynamics vary by mouse strain" would help position the paper better; I don't think I will be alone in expecting to see a mechanistic dissection of germline control of tumour evolution after reading the current title of "Genetic background sets the trajectory of cancer evolution"

Open Access This Peer Review File is licensed under a Creative Commons Attribution 4.0 International License, which permits use, sharing, adaptation, distribution and reproduction in any medium or format, as long as you give appropriate credit to the original author(s) and the source, provide a link to the Creative Commons license, and indicate if changes were made.
In cases where reviewers are anonymous, credit should be given to 'Anonymous Referee' and the source.
The images or other third party material in this Peer Review File are included in the article’s Creative Commons license, unless indicated otherwise in a credit line to the material. If material is not included in the article’s Creative Commons license and your intended use is not permitted by statutory regulation or exceeds the permitted use, you will need to obtain permission directly from the copyright holder.
To view a copy of this license, visit https://creativecommons.org/licenses/by/4.0/

Version 0:

We thank the three reviewers for their time, insights, enthusiasm and constructive feedback. Motivated by these comments, the revised version of the manuscript has a series of new analyses that substantially reinforce and extend our previous findings. Key additions include:

1. Leveraging the nested hierarchical relationships of strain-litter-animal sharing between tumours to robustly separate genetic (strain) and aspects of shared environment (litter, animal) effects on tumour development. These new analyses have been incorporated through all results sections of the manuscript and strengthen our original findings.
2. The analysis of gene expression changes has been substantially expanded, defining expression signatures that are variable between tumours and identifying the underlying causes including genetic background, specific driver mutation, and circadian cycles.
3. Refinement and expansion of analyses exploring clonal dynamics during tumour development, including the introduction of neutral expectations for subclonal selection.
4. Identifying epistatic interactions between genetic background and driver mutation that generate divergent transcription signatures enriched in critical cancer pathways, including p53 cellular stress response.

We have worked to improve the impact and clarity of the manuscript. Throughout, we better emphasise that our unique and powerful study design is intended to reveal (1) the extent to which tumours consistently develop given the same environment, and (2) how polygenic divergence leads to conservation or divergence in mutagenesis, cancer development, and clonal selection. We have restructured our Introduction to better clarify that this work is not intended or designed to identify monogenic causes of strain differences in cancer susceptibility; such insights would be academically interesting but of narrower appeal than the original and impactful study that we present. Finally, our revised Discussion showcases how our uniquely powerful study design complements what is feasible in clinical studies, and how our new findings might inform future studies that span diverse human ancestry backgrounds.

Referee #1 (Remarks to the Author):

This work analyzes dependencies of cancer evolution on genetic background. The authors use their well-established model of DEN-induced liver cancer in mice. All four strains included in the comparison stably develop tumors following a pulse of DEN mutagenesis but differ in length of the latency period. All four strains develop driver mutations in the MAPK pathway, show similar expression changes of the MAPK pathway gene transcripts and feature highly concordant copy number variants (both gains and losses). At the same time, presence of WGD, number of drivers, usage of driver genes and specific driver mutations all depend on genetic background. Some of these observations are evident from the earlier work by the same authors, but this manuscript analyzes additional strains and puts the genetic background dependency in focus. Overall, this is an important topic, and I found the paper of great interest.

We thank the reviewer for their assessment of our manuscript and for recognising the work as important and of great interest. In the revised manuscript, we have fully addressed all of the specific points raised below.

At the same time, the analysis and presentation can be greatly improved. Also, the manuscript offers very little mechanistic insight.

We agree that aspects of the analysis and presentation could be improved, which we have addressed and implemented as detailed below. Additionally, this referee's suggestion to make use of the inherent nested structure of the strain-litter-animal-tumour relationships has been pivotal in refining the analysis and presentation through the revised manuscript, and resulting in new biological and mechanistic insights.

Regarding mechanism, we realise that our original presentation did not clearly highlight the broad mechanistic insights into the consistency of tumour development and how that is influenced by polygenic divergence between strains. Inspired by the reviewers' feedback, new analyses add further mechanistic insights and we have made several improvements to better showcase the key mechanistic insights, including:

- How DEN2 mutations are likely caused when the Mgmt DNA repair enzyme system is overwhelmed (**new Extended Data Fig. 1c-e**).
- The mechanistic origins of non-template strand bias of most driver mutations and the evasion of transcription coupled repair by *Hras* Q61R driver mutation arises from evasion of transcription coupled repair (**updated Fig. 5e**, **Extended Data Fig. 6a**).
- How CAROLI whole genome duplications (WGD) occur in the very first cell-cycle following DEN exposure (**revised Fig. 3c,d** and **new Extended Data Fig. 3a**). And a clear, statistically robust demonstration that WGD is associated with higher aneuploidy rate (**new Fig. 3e**).
- Identified that clonally distinct tumours maintain the circadian coordination of transcription (**new Fig. 6c** and **new Extended Data Fig. 11a,b**).
- How strain differences in driver mutations seem to arise from epistatic interactions between MAPK activating mutations and genetic background that alter the transcription key cancer associated pathways of p53, PPAR and TGF-β signalling (Expanded gene expression results section, **new Fig. 6b-f**, and **new Extended Data Fig. 10a,b**).

1) The manuscript presents the analysis of 581 tumors. If I am not mistaken, the earlier publication analyzed over 350 tumors. It would be helpful to know how many data points are new.

Our earlier publications[1–3] analysed whole genome sequencing from 371 DEN-induced C3H tumours, and Aitken *et al.*, 2020[1] included limited use of 84 DEN-induced CAST tumours as a replication cohort, but not for inter-strain comparison. This new study adds whole genome sequencing and digitised histopathology from 72 DEN-induced CAROLI tumours and 55 DEN-induced BL6 tumours, as well as paired whole transcriptome sequencing from the tumours of all four strains (n=453). We also report for the first time a wealth of complementary genomic, transcriptomic, and functional-genomic datasets from corresponding normal tissues as summarised in the table below (**Reviewer Table 1**).

Reviewer Table 1 (see also Supplementary Table 2)

**Description**	**Archive**	**Accession**	**First reported**
Histology images of tumours from DEN mutagenised C3H/HeOuJ mice	BioStudies, EMBL-EBI	S-BSST383	Aitken *et al.*, 2020[1]
Histology images of tumours from DEN mutagenised C57BL/6J mice	BioStudies, EMBL-EBI	S-BSST384	This study
Histology images of tumours from DEN mutagenised CAST/EiJ mice	BioStudies, EMBL-EBI	S-BSST383	Aitken *et al.*, 2020
Histology images of tumours from DEN mutagenised CAROLI/EiJ mice	BioStudies, EMBL-EBI	S-BSST384	This study
Whole genome sequence and mutation calls from tumours of DEN mutagenised C3H/HeOuJ mice, along with control samples	European Nucleotide Archive	PRJEB37808	Aitken *et al.*, 2020
Whole genome sequence and mutation calls from tumours of DEN mutagenised C57BL/6J mice, along with control samples	European Nucleotide Archive	PRJEB15138	This study
Whole genome sequence and mutation calls from tumours of DEN mutagenised CAST/EiJ mice, along with control samples	European Nucleotide Archive	PRJEB37808	Aitken *et al.*, 2020
Whole genome sequence and mutation calls from tumours of DEN mutagenised CAROLI/EiJ mice, along with control samples	European Nucleotide Archive	PRJEB15138	This study
RNA-seq data from 15-day old C3H/HeOuJ mouse liver	ArrayExpress	E-MTAB-8518	Aitken *et al.*, 2020
RNA-seq data from 15-day old C57BL/6J mouse liver	ArrayExpress	E-MTAB-16391	This study
RNA-seq data from 15-day old CAST/EiJ mouse liver	ArrayExpress	E-MTAB-8518	Aitken *et al.*, 2020
RNA-seq data from 15-day old CAROLI/EiJ mouse liver	ArrayExpress	E-MTAB-16391	This study
RNA-seq data from 190-day old C3H/HeOuJ mouse liver	ArrayExpress	E-MTAB-16391	This study
RNA-seq data from 267-day old C57BL/6J mouse liver	ArrayExpress	E-MTAB-16391	This study
RNA-seq data from 281-day old CAST/EiJ mouse liver	ArrayExpress	E-MTAB-16391	This study
RNA-seq data from 561-day old CAROLI/EiJ mouse liver	ArrayExpress	E-MTAB-16391	This study
RNA-seq data from tumours of DEN mutagenised C3H/HeOuJ mice	ArrayExpress	E-MTAB-16391	This study
RNA-seq data from tumours of DEN mutagenised C57BL/6J mice, along background liver tissue	ArrayExpress	E-MTAB-16391	This study

RNA-seq data from tumours of DEN mutagenised CAST/EiJ mice	ArrayExpress	E-MTAB-16391	This study
RNA-seq data from tumours of DEN mutagenised CAROLI/EiJ mice	ArrayExpress	E-MTAB-16391	This study
CTCF ChIP-seq data from 15-day old C3H/HeOuJ mouse liver	ArrayExpress	E-MTAB-11959	Anderson *et al.*, 2024[2]
H3K4me3 ChIP-seq data from 15-day old C3H/HeOuJ mouse liver	ArrayExpress	E-MTAB-14454	This study
H3K27ac ChIP-seq data from 15-day old C3H/HeOuJ mouse liver	ArrayExpress	E-MTAB-14454	This study
CTCF ChIP-seq data from 15-day old C57BL/6J mouse liver	ArrayExpress	E-MTAB-14454	This study
H3K4me3 ChIP-seq data from 15-day old C57BL/6J mouse liver	ArrayExpress	E-MTAB-14454	This study
H3K27ac ChIP-seq data from 15-day old C57BL/6J mouse liver	ArrayExpress	E-MTAB-14454	This study
CTCF ChIP-seq data from 15-day old CAST/EiJ mouse liver	ArrayExpress	E-MTAB-14454	This study
H3K4me3 ChIP-seq data from 15-day old CAST/EiJ mouse liver	ArrayExpress	E-MTAB-14454	This study
H3K27ac ChIP-seq data from 15-day old CAST/EiJ mouse liver	ArrayExpress	E-MTAB-14454	This study
CTCF ChIP-seq data from 15-day old CAROLI/EiJ mouse liver	ArrayExpress	E-MTAB-14454	This study
H3K4me3 ChIP-seq data from 15-day old CAROLI/EiJ	ArrayExpress	E-MTAB-14454	This study
H3K27ac ChIP-seq data from 15-day old CAROLI/EiJ	ArrayExpress	E-MTAB-14454	This study
ATAC-seq data from 15-day old C3H/HeOuJ mouse liver	ArrayExpress	E-MTAB-11780	Anderson *et al.*, 2024
ATAC-seq data from 15-day old C57BL/6J mouse liver	ArrayExpress	E-MTAB-14144	This study
ATAC-seq data from 15-day old CAST/EiJ mouse liver	ArrayExpress	E-MTAB-14144	This study
ATAC-seq data from 15-day old CAROLI/EiJ mouse liver	ArrayExpress	E-MTAB-14144	This study
ATAC-seq data from tumours of DEN mutagenised C57BL/6J mice, along background liver tissue	ArrayExpress	E-MTAB-14144	This study

We now explicitly state the summary numbers of new and previously published datapoints in the **Methods** section under "Data availability", and **Supplementary Table 2** indicates in which study each collection of datapoints was first described (29 datasets are described for the first time in this study).

2) The presentation of results would benefit from displaying the differences between strains normalizing to inter-individual variation within strains (ideally also showing difference in littermates vs. unrelated mice). For example, one can estimate the proportion of variance explained by genetic background.

The reviewer raises an important point, which motivated a valuable new set of analyses. Not only do we have tumours to compare between strains but we also have multiple tumours per animal and multiple animals per litter. This nested structure was documented in original **Supplementary Table 1** but is now graphically visualised in the revised main text **Fig. 1d**. As suggested, we now make use of this nested structure of tumour relatedness. Because all tumours from the same animal necessarily share the same litter and strain, classic multivariate regression using animal, litter and strain as independent variables is not appropriate. Instead, we apply nested linear mixed-effects models with likelihood ratio tests to accurately separate the independent influence of these parameters. We illustrate the applicability and power of this approach through simulation in **Reviewer Fig. 1**.

This insightful additional analytic strategy has now been incorporated throughout the revised manuscript to explore the variance in mutation burden (**Fig. 2d,e**), oncogenic driver mutation (**Fig. 4e,f**), gene expression (**Fig. 6c**), and for measures of selection (**Fig. 7d**, **Extended Data Fig. 12f,g**), and telomere length (**Extended Data Fig. 3d**). In each case, we find the predominant - and often only - significant covariate is the strain, which substantially reinforces the main conclusions from this manuscript - that the genetic background sets the trajectory for cancer evolution.

In addition to the simulations (**Reviewer Fig. 1**), this hierarchical strain-litter-animal-tumour approach is experimentally validated by the discovery that circadian gene expression effects are associated with both animal and litter. In turn, this correlates with the timing of tumour isolation, reflecting the contemporaneous collection of tumours from the same animal and litter (**Fig. 6f**, **Extended Data Fig. 11a,b**).

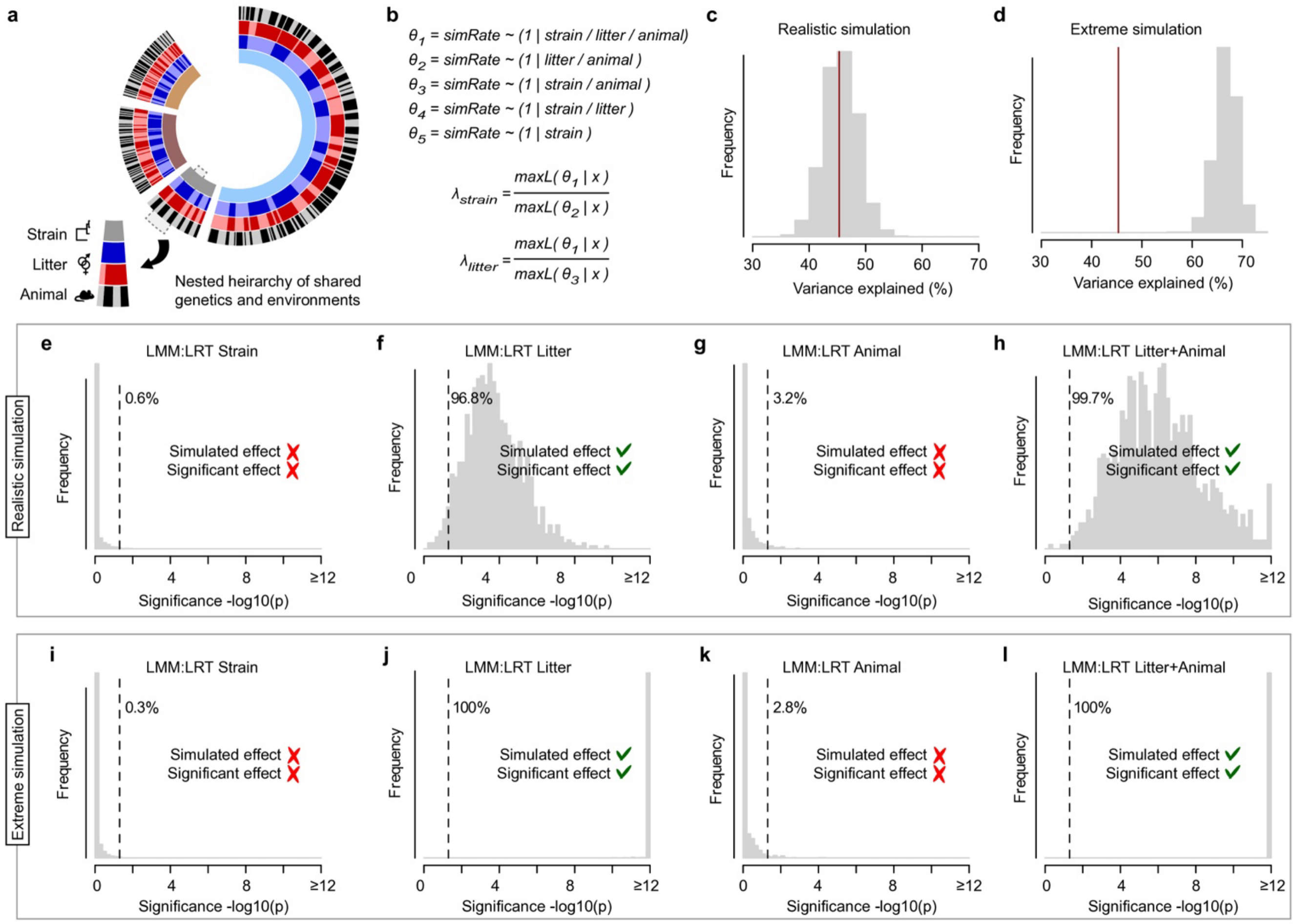

Reviewer Fig.1 | Linear mixed-effects models and likelihood ratio tests accurately separate the significant from non-significant variables. a, Clonally distinct DEN-induced tumours from inbred mice have a hierarchy of shared environment (animal, litter) and genetics (strain). All tumours from the same animal necessarily share the same litter and strain, so these are not independent variables. **b,** Linear mixed-effects models (LMM) can be applied in this setting. The comparison between nested models provides a statistically robust way to test for a correlation between an outcome variable (e.g. simulated mutation rate, simRate) and a predictor variable (e.g. litter) that is independent of other, potentially correlated predictor variables (e.g. strain or animal). Model structure is shown using lmer notation[4]. Here, an unrestricted model Θ_1 containing strain, litter, and animal is fit with maximum likelihood (maxL) to a dataset (x). A nested model Θ_3 is similarly fit with maxL to x, Θ_3 is identical to Θ_1 except that the litter variable is removed. The likelihood ratio (λ_{litter}) is then used to test (likelihood ratio test) if the observed variance is better explained by the unrestricted than the restricted model, taking into account the difference in model complexity (degrees of freedom). **c,** Simulations of litter-only effects (n=1,000, grey histogram) with the total variance (x-axis) explained by litter based on univariate linear regression. Data shown for ten iterations of mutation rate shuffling which approximately equates to the level of variance explained in the real data by correlation with strain (brown line). These simulations are subsequently referred to as “realistic simulations”. **d,** As for **c** but after only four iterations of mutation rate shuffling, consequently the simulated data shows a greater percentage of variance explained by litter, than the real data correlation between strain and mutation rate (brown line). These are subsequently referred to as “extreme simulations”. **e,** Frequency (y-axis) distribution of p-values (x-axis) from 1,000 simulations of litter specific effects on mutation rate. Green ticks and red crosses denote true and false, respectively, for whether a strain specific effect is

expected from the simulation and if it is detected in >5% of simulations at $p < 0.05$ after Bonferroni correction. Under a well behaved test, simulated and significant should match. Dashed line indicates Bonferroni corrected threshold for statistical significance ($p < 0.05$). Percentage value indicates the percent of simulations that passed the significance threshold (0.6%). **f-h**, Plots in the same style as **e** based on the same simulations, but testing for associations with litter (**f**), animal (**g**) or combined litter+animal effects (**h**). Fewer than 5% of tests pass the Bonferroni corrected $p<0.05$ significance threshold for strain and animal associations, demonstrating the statistical test and multi-testing correction appropriately control the false discovery rate. In the case of litter-only effects (**f**) the simulations indicate 96.8% power to detect an association of the same magnitude as the relationship between strain and mutation rate observed in the real data. **i-l**, as the corresponding panels above but based on the extreme, rather than realistic, simulations. This demonstrates that even with strong associations and partially correlated variables the LMM framework accurately attributes variation to the correct (litter or litter+animal) rather than correlated, incorrect (strain or animal) variable.

Methods for Reviewer Figure 1

Simulations (n=1,000) were conducted where the observed strain-litter-animal-tumour structure of the study was preserved and observed measures of tumour mutation rate were reassigned to tumours: (1) Mutation rates were numerically sorted into a list, then randomly re-shuffled in sliding windows of 85, the re-shuffling iteratively applied to progressively degrade the rank order of mutation rates (up to 11 iterations of shuffling). (2) A litter identifier was picked at random, the top mutation rates from the list (count corresponding to the number of tumours from animals in the litter) were allocated in random order to the tumours from that litter. Allocated mutation rates were removed from this list. The process repeated by picking litters without replacement until all mutation rates from the list were allocated to tumours.

A simple linear regression between litter as a factor and mutation rate as a continuous variable was calculated using the lm function from R. The distribution of variance in mutation rate explained by litter in the regression model was compared to the variance in mutation rate explained by strain in the real data. Simulations with 10 iterations of shuffle were found to approximately equate to the variance observed in the real data (**Reviewer Fig. 1c**) so those simulations were selected as the "Realistic simulation" set for comparison and power analysis.

3) The analysis of gene expression is currently limited to the presentation of first two principal components highlighting major sources of expression variance among the samples. It is not clear whether the effects of tumorigenesis on gene expression differ due to genetic background (the main topic of the paper).

Addressing this point, we have now included substantial new independent component (IC) analysis of gene expression, both amongst the tumours and between tumours and non-tumour liver tissue. This is graphically summarised in the revised **Extended Data Fig. 10**. The IC analysis reveals coordinated differences in gene expression, many of which are associated with strain (**Extended Data Fig. 10a,b**).

Remarkably we found that, despite broadly uniform transcriptional changes in the MAPK pathway, there are pronounced epistatic interactions between somatic driver mutations and germline genetic background. These result in divergent transcription signatures, including those enriched for genes involved in the cellular stress response (p53, IC11) and mitogenic signalling (proliferator-activated receptor (PPAR) and TGF-β, IC16) (expanded gene expression results section and **new Fig. 6b-f**; **new Extended Data Fig. 10a,b**). In other words, an identical driver mutation can confer a different

growth/survival advantage depending on the polygenic background in which it occurs, which provides a plausible mechanistic basis for strain differences in driver mutation choice and tumour latency.

Combining the new expression signature analysis with the hierarchical strain-litter-animal-tumour models uniquely allows gene expression correlations to be explored across tumours from within the same animal and between animals of the same litter that shared *in utero* and cage environments (**Fig. 6c**). For example, this could be testing for evidence of immune or metabolic cross-talk between tumours. Along these lines we find a subset of gene expression signatures that associate with litter and animal that demonstrate the preservation of circadian transcriptional regulation in tumours and its coordination between tumours in the same animal (**Fig. 6c**), which is consistent with the documented time of tumour isolation (**Extended Data Fig. 11a,b**).

The extensive and insightful new analyses of gene expression have lengthened the corresponding results sections. To accommodate this and maintain a focused manuscript we have moved the analysis of mutation rates in CTCF, promoter, and enhancer regions to a **Supplementary Text** document.

4) Is it possible to expand the analysis of genetic differences within driver genes beyond the window of total conservation? Does the number of amino acid substitutions track differences in usage of individual driver mutations? Is the structural neighborhood more informative than the sequence neighborhood?

In terms of the "structural neighborhood", we think the referee is asking about within-gene epistatic interactions between potentially oncogenic amino acid substitutions and strain differences in the germline encoded amino acid sequence of the proto-oncogene. This is an interesting hypothesis, but we do not find any evidence for it to be the case. For example, *Hras* shows striking differences in driver mutation utilisation within the gene between C3H and BL6 (**Fig. 5a**) yet the encoded orthologous proteins are 100% identical in amino acid sequence in the germline genomes. The amino acid sequence of Hras is 100% identical between C3H, BL6, CAST, and human. CAROLI has one germline encoded amino acid substitution compared to those other strains and species, R169Q, but CAROLI is not an outlier in *Hras* driver utilisation.

Similarly, the amino acid sequence of Egfr is 100% identical between C3H, BL6, and CAST germlines, and 99% identical between those and CAROLI. The *Egfr* F254I mutation is recurrently observed in C3H, BL6, and CAST but not CAROLI, whereas other potentially oncogenic Egfr amino acid substitutions are observed in CAROLI. However, although CAROLI is an outlier in terms of both Egfr germline amino acid sequence and driver mutation occurrence, the immediate structural neighbourhood of F254 is fully conserved between strains within the Egfr molecule (no amino acid changes within 22 Å of F254): There are 7 amino acid differences in the extracellular domain of Egfr between BL6 and CAROLI: R72K, T161S, M173L, D412E, I463V, V502M, and A566T. Using the Alphafold all-atom model of BL6 Egfr (accession:AF-Q01279-F1-v4), the closest Euclidean distance between any atom of F254 and each of these non-conserved amino acid positions is 38.0, 29.3, 22.7, 54.8, 55.7, 62.8, and 34.9 Å, respectively, from the N-terminus. While we cannot exclude the possibility that structurally distant amino acid substitutions interact allosterically with *Egfr* F254I, we considered this too speculative to include in the revised manuscript.

5) The story of the strand bias of HRAS Q61 mutations is fascinating. Some Q61L tumors are symmetric. Does this affect the inference of the strand bias? Also, the idea of "the production of pro-oncogenic miscoded proteins before replication" is confusing. Do the authors imply that Q61L and

Q61R are driven by different lesion types that result in different transcripts over the lesion-containing template strand? Is there any evidence of transcription over a DEN-induced lesion?

We agree with the referee that the utilisation and properties of the *Hras* Q61 mutations are a fascinating case study of differences in driver mutation occurrence and selection.

We have previously published evidence that DEN-induced lesions can be transcribed over without stalling RNA polymerase[2,3] and it has been established that damaged DNA can provide an error-prone template for the production of mis-coded oncogenic proteins[5,6]. In the revised manuscript we have been more explicit in the last sentences of the corresponding results paragraph, which now reads: "*Thus, mutated oncogenic proteins would not be produced until after the first round of replication following damage. In contrast, the Hras Q61R mutations arise from template strand damage and therefore error-prone transcription over that damage could lead to the production of pro-oncogenic miscoded proteins before replication*[6]".

The analysis of template versus non-template lesion strand bias in **Fig. 5d,e** and **Extended Data Fig. 6a** only considers those regions where the focal driver mutation is in a mutationally asymmetric genomic interval in that particular tumour genome (i.e. the driver region/chromosome retained either two lesion-bearing forward strands or two lesion-bearing reverse strands (see Aitken *et al*., 2020, Fig. 2b,d[1]); tumours where the driver is in a mutationally symmetric region (one forward and one reverse strand) are excluded, described in the Methods). Instead, the lack of lesion strand bias between template and non-template stands for *Hras* Q61L indicates that, in aggregate between asymmetric tumours, there are approximately equal numbers of tumours with template strand lesions and tumours with non-template stand lesions (rather than being unbiased/symmetric within a given tumour genome). We have rephrased the legend to **Fig. 5e** to emphasise this.

6) The finding of differences in distributions of times to MRCA between strains is of clear interest. However, additional details would be welcome here. Is it possible to analyze these differences separately for different drivers (to control for the driver-specific fitness advantage)? Also, is it possible to analyze the difference between tumors with one known driver vs. two known drivers (I understand that the former will be dominated by C3H tumors)?

We also think that this discovery is interesting and, in addition to the dispersion test presented previously (now **Extended Data Fig. 12d**) we have now (thanks to this reviewer's earlier comment) used the nested nature of our data to orthogonally test, and confirm, that the fraction of MA segments (MRCA generation) shows a very strong strain effect that is independent of litter and/or animal effects (**Fig. 7d**).

Directly addressing the reviewer's point about one-driver versus two-driver tumours, we have added **Fig. 4f** which shows that beyond the strain association for driver count there is not a significant animal or litter effect, a conclusion robust to whether copy-number changes are included or not.

Addressing this reviewer's request to provide further details on the relationships between times to MRCA and driver genes, we have extended the mixed-effects models approach to consider our key selection measures in the context of gene expression signatures, driver identity, driver multiplicity, and aneuploidy, all conditioned on strain (**Extended Data Fig. 12f,g**). Having conditioned on strain (already shown to be an almost ubiquitously significant effect), the only additional association robust to multiple testing correction was the enrichment of aneuploidy in late MRCA generation tumours.

7) The "dispersion test" is creative. Does it have sufficient power to detect partial loss of lineages? It Is not immediately obvious that the quantitative effect on dispersion would be strong enough. Is it possible that the analysis is confounded by differences in somatic recombination rates? Unless it is too demanding, computer simulations may be helpful here.

Following this reviewer's suggestion we have now implemented a series of simulations to explore partial lineage loss and the influence of sister chromatid exchange on estimates of dispersion (**Extended Data Fig. 12b,c**; **Reviewer Fig. 2**). We consider the potential impact of both sister chromatid exchanges occurring:

1. Before the first mitotic division, "generation 0 SCEs", which generate switches in mutational asymmetry[1] and are detectable by our first-submission segment-calling approaches.
2. After the first mitotic division, "cryptic SCEs", which do not generate switches in mutational asymmetry and hence were not detectable by our first-submission approaches.

These simulations confirmed the reviewer's hypothesis that differences in (generation 0 and cryptic) SCE rates could influence dispersion-based estimates of selection, as presented in the original version of the manuscript. However, the effect is modest. As our comparisons were relative measures between strains, they would only present a problem if SCE rate systematically differed between strains (we previously noted that CAROLI had significantly fewer SCEs per genome than the other strains).

Addressing this, we have revised the dispersion analysis so that mutation asymmetry segments are not analysed as monolithic blocks, but are fragmented into consecutive windows, each with a uniform number of mutations (see Methods). This enables stringent filtering of windows which may contain a cryptic SCE breakpoint, which would distort the MA rate of the window relative to upstream and downstream windows. We now present multiple simulations to illustrate the statistical power of this approach, its robustness to observed and cryptic SCEs, and its ability to detect subclonal selection in later generation MRCA tumours (**Extended Data Fig. 12b,c**, **Reviewer Fig. 2**).

Our updated framework tested each tumour individually for non-neutral lineage retention, meaning that we are no longer confined to relative measures of selection between strains (**Fig. 7c**, **Extended Data Fig. 12e**). The corresponding results and methods sections have been updated, and new panels added to **Fig. 7** and **Extended Data Fig. 12**.

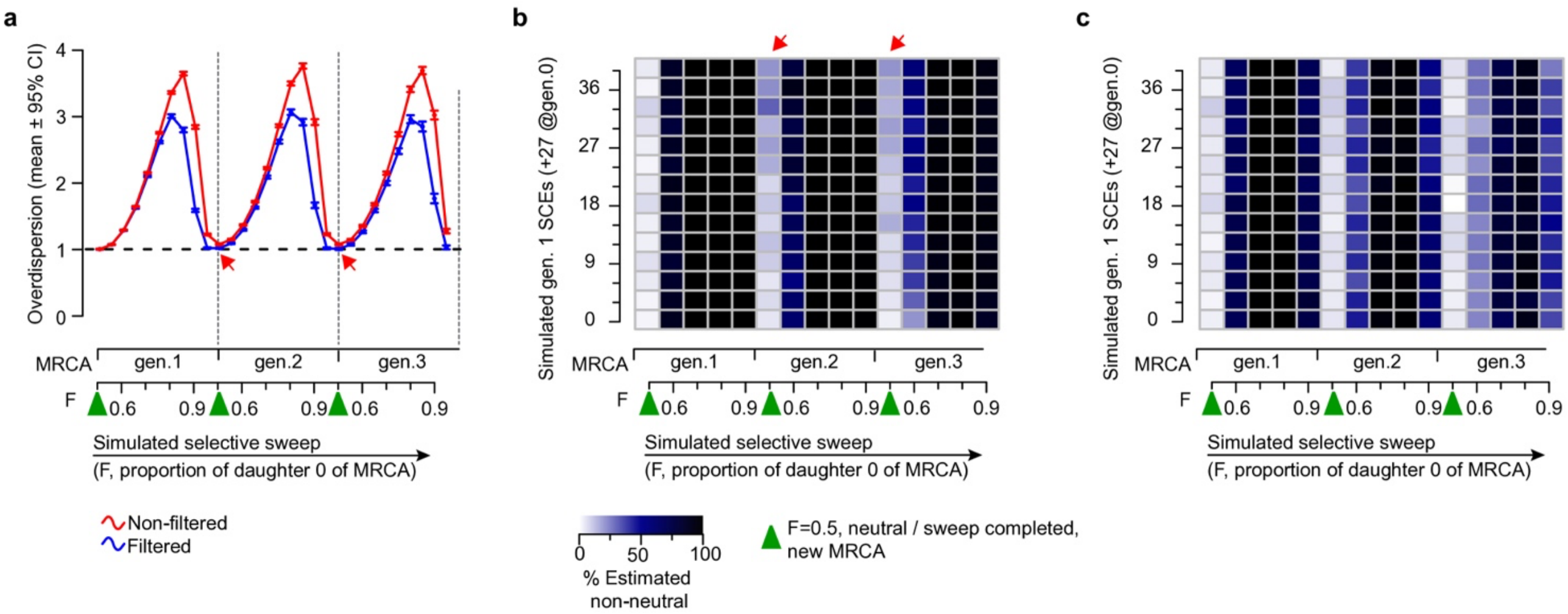

Reviewer Fig.2 | Simulated tumour evolution demonstrating statistical power and robustness of multiallelic rate dispersion tests. a, Simulation of tumour evolution from a single mutagenised cell, modelling lesion segregation, multiallelic variation and sister chromatid exchange (SCE), with different levels of selection (x-axis; the measure F denotes the proportion clonal contribution of the dominant daughter from the MRCA, F=0.5 equal contribution and neutrality, F>0.5 progressively unequal contributions and a stronger/more-complete selective sweep). Green triangles denote the transition between an extreme selective sweep and complete lineage loss, leading to the definition of a later-generation MRCA. Estimated overdispersion (y-axis) as the average over 2,800 simulations for the scenario of 27 SCE in generation 0 (27 is the median count observed in tumours, which are visible through mutation asymmetry) and encompassing between 0 and 38 SCE events in generation 1, with 95% confidence intervals (whiskers) from bootstrap sampling. Non-filtered (red) measures exclude the window-based filtering designed to detect and exclude cryptic SCE events. Filtered (blue) measures represent the approach used in the revised manuscript. Red arrows point to modest inflation of overdispersion measures at F=0.5 in the non-filtered analysis that lead to erroneous rejection of neutral evolution, a problem solved by the filtered analysis. **b,** Power to detect non-neutral evolution. Heatmap showing the percentage of simulations that reject the null-hypothesis of neutral (post-MRCA) evolution at p<0.05 under the truncated binomial model (see Methods). Data shown for non-filtered measures (panel **a**, red curve), red arrows indicate inflated rejection of neutrality for high rates of generation 1 SCEs when filtering is not implemented. Green arrows indicate the simulation of neutral evolution (F=0.5) at each MRCA generation. **c,** Heatmap plotted as in panel **b** for filtered analysis (panel **a**, blue curve). Implementing the window-based filtering appropriately controls the false discovery of non-neutral evolution even in the extreme scenarios of high generation 0 and generation 1 SCE rates.

Referee #2 (Remarks to the Author):

This study compares the genomic and transcriptomic features of mutagen-induced (DEN) liver tumours between four mouse strains. Tumours in all strains show convergent activation of MAPK signalling, then the paper reports inter-strain differences in other molecular features: driver mutations, genome doubling, subclonal selection. The authors conclude that germline variation strongly determines the course of tumour evolution.

The authors' liver cancer consortium has generated an incredible resource, that this paper is further evidence of the value of. There is a lot of data in this manuscript, and the authors do a very good job at constructing an easy-to-follow narrative from it, clearly explaining key features in the data.

We thank this reviewer for recognising the value of our work, the scale of the datasets we have generated (which are all deposited in open access databases), and for highlighting the clarity of the manuscript.

The paper is very convincing that inter-strain differences in tumour evolution exist - but I'm not convinced this is a novel finding in general (explained below).

I think a major limitation of the manuscript is that it provides very little mechanistic insight into *why* the inter-strain differences exist. I really wanted to see a specific consideration of precisely what the germline variation between species is, what its function is, and how this causes differences in tumour evolution. At present this is not explored at all.

In the revised manuscript we have added two key additional sets of analyses which provide several additional mechanistic insights (i.e. why the inter-strain differences exist) and further strengthen our original findings. Specifically, we used the hierarchical nested structure of our data (strain-litter-animal-tumour relationships) and also substantially extended the transcriptome analyses. Our two key new mechanistic insights are:

1. Genetic background (rather than common animal- or litter-environment) sculpts the landscape of mutagenesis and early oncogenic selection, and even modest germline differences between strains dramatically shifts the profile of clonal selection and course of tumour evolution. We incorporated the strain-litter-animal-tumour analysis framework into each results section of the revised manuscript. Applying likelihood ratio tests of nested mixed-effects models identified that genetic background is the only significant source of differences across all aspects of mutagenesis and selection, rather than animal- or litter-specific effects (**new Figures: Fig. 1d, Fig. 2d,e, Fig. 4e,f, Fig. 6c,** and **Fig. 7d**, **Extended Data Fig. 3d,** and **Extended Data Fig. 12f,g**).
2. We newly reveal how epistatic interactions between drivers and genetic background shape tumour development. Independent component analysis of the tumour transcriptomes identified 17 expression signatures that vary between DEN induced tumours. Despite the broad equivalence of different MAPK driver mutations on gene expression that we originally reported, the deeper analysis guided by the reviewers' suggestions has now discovered additional epistatic interactions between driver (somatic) mutations and genetic background (germline variation). This results in differences in key signalling pathways, including the induction of p53 and peroxisome proliferator-activated receptor (PPAR) and TGF-β (Expanded gene expression results section and **new Fig. 6c-f**; **new Extended Data Fig. 10a,b**). In other words, an identical driver mutation can confer a different growth/survival advantage depending on the polygenic background in which it occurs, which provides a plausible mechanistic basis for strain differences in driver mutation choice and tumour latency.

In addition to these new insights, we have extended, strengthened, and more clearly articulated our existing mechanistic findings:

3. Strain-specific differences in the expression of DNA damage repair enzymes (e.g. Mgmt) cause a shift in the mutation spectrum, specifically of the G-rich DEN2 signature (**new Extended Data Fig. 1c-e**).
4. Efficient transcription coupled nucleotide excision repair means most driver mutations are biased to damage on the non-template strand of DNA, but *Hras* Q61R driver mutations evade such repair (**Fig. 5e**, **Extended Data Fig. 6a**).
5. Whole genome duplication (WGD) is pervasive in CAROLI tumours, and usually occurs in the very first cell-cycle following DEN exposure (**revised Fig. 3c,d** and **new Extended Data Fig. 3a**). In turn, this generates genome instability which results in an increased frequency of secondary aneuploidies in these tumours (statistical testing shown in **new Fig. 3e**).
6. Telomere length varies between strains at baseline, and there are divergent dynamics during tumorigenesis. Telomere shortening in CAROLI, associated with prior observations of telomere damage and length crisis, is associated with WGD (**new Extended Data Fig. 3b,c** and new paragraph in the WGD results section). Whereas, telomere lengthening may play a role in selection, specifically in the C3H strain (**new Extended Data Fig. 12h**).
7. The strain-litter-animal-tumour framework allows us to detect coordinated activity between tumours within the same animal, for example coordinated immune clearance of shared driver mutations, selection pressure or mutation burden. Most notably, we found that circadian rhythm related genes maintain synchronised coordination in tumours (**new Fig. 6c**, **new Extended Data Fig. 11a,b**), consistent with the time of day of tissue collection.
8. Simulations of tumour evolution identify that genetic background effects on subclonal dynamics are due to selection through lineage-loss during the earliest stages of tumour initiation (**Fig. 7** and **Extended Data Fig. 12**).

Further to these mechanistic insights we think this work also presents an important conceptual advance. For example, discovering that epistatic interactions between genetic background (germline variation) and driver (somatic) mutations perturb gene expression of key cancer pathways and the occurrence of whole genome duplication provides a plausible mechanistic basis for strain differences in driver mutation choice and tumour latency. Furthermore, if an individual's genetic background influences how a driver mutation activates other cancer pathways, this may explain divergent outcomes in precision medicine strategies despite targeting specific oncogenic mutations.

The final sentence of the introductory paragraph says "it is not clear how consistently cancer would develop given the same genetic background and same environment, or how germline differences influence the trajectory to tumourigenesis". I think this point is oversold: there is already a big literature on this exact question.

We agree that this is an area of wide interest and in the revised manuscript we have cited more of the existing literature in the introduction, and contextualised our results more fully in the discussion. As described in detail below, we have considered how our work relates to other work in the field, highlighting that the majority of human cancers occur sporadically, in the absence of high-penetrance germline alterations that typify both genetically engineered mouse models of tumourigenesis and human predisposition syndromes. Where our study is especially novel is that it is considering the cumulative effects of polygenic divergence on the trajectory of tumourigenesis, which is more typical of the divergence between most humans and human groups. Furthermore, our prospective experimental design allows us to separate genetic vs controlled environmental influences to establish the basis of the observed differences, which is, to our knowledge, conceptually novel.

Specifically:
There is a large literature on strain dependence in mouse models, across cancer types (perhaps most famously the mom locus in apc/min models of intestinal cancer: https://www.sciencedirect.com/science/article/pii/0092867493904848)
There is also literature on how mouse germline genetics alters tumour biology, for instance this manuscript describing metastases suppression by germline variants: https://www.nature.com/articles/nature20792

We agree that there are many previous studies of strain dependence in mouse cancer models, and that they have been insightful in identifying genes responsible for cancer causation and resistance. As summarised in van der Weyden *et al.*[7], key genetic risk/resistance loci have been identified, but the mechanism by which these genes actually modulate cancer is much more obscure. Our work is complementary to these studies, providing a different approach to dissect these complex biological questions. Given the polygenic nature of divergence between strains C3H, BL6, CAST, and CAROLI (spanning millions of SNPs) we do not expect phenotypic features of tumour development to neatly map to individual loci of major effect and the study design is not one that could reveal such loci - for that F2 or recombinant inbred lines would likely be needed, and would not actually be possible with these strains as the *Mus caroli* species cannot breed with the three *Mus musculus* strains. Instead, there will be multiple - and interacting - downstream consequences.

For example, even in the well-characterised Apc^{Min} mouse model[8], the *Mom-1* locus only accounts for ~50% of the genetic variation in tumour load and other putative modifiers exist in BL6 mice[9], and the mechanism(s) of interaction remain obscure. An alternative approach, as pursued by van der Weyden *et al.*[10] and others, is to systematically consider hundreds of individual gene mutants in otherwise controlled conditions (predominantly BL6 background). Such studies give insight into Mendelian-like genetics, but cannot consider the epistatic interactions between the individual gene effects and the polygenic genetic background in which they arise.

Addressing this gap, rather than to decipher locus specific causation (which, while intellectually interesting are unlikely to be directly translational), we asked: (i) how consistently tumours develop within a genetic background and (ii) the consequences of polygenic variation on processes such as mutagenesis and selection. Our study identified mechanistic insights into the genetic and evolutionary stability of damage induced mutation signatures, driver gene pathways, gene expression programmes, and selection processes.

The molecular analysis in this new manuscript is more comprehensive that anything I have seen before, but arguably only incrementally so.

We thank the reviewer for recognising the richness of the datasets that we generated, analysed, and deposited to be publicly available as part of this manuscript. In addition to data published in our previous work[1–3], for the current manuscript we generated an additional 29 complementary genomic, transcriptomic, functional-genomic, and histological datasets - representing hundreds to thousands of individual sequencing and image files. These datasets are summarised in **Reviewer Table 1** (above) and with full details also provided in the manuscript (Methods - "Data availability"; **Supplementary Table 2**). The raw data files for all 29 new datasets are available from ArrayExpress, the European Nucleotide Archive (ENA), and BioImage Archive at the EMBL-EBI.

Specifically, our earlier publications[1–3] analysed whole genome sequencing from 371 DEN induced C3H tumours, and Aitken *et al.*, 2020[1] included limited use of 84 DEN induced CAST tumours as a replication cohort, but not for inter-strain comparison. This new study adds whole genome sequencing from 72 DEN induced CAROLI tumours and 55 DEN induced BL6 tumours, paired whole transcriptome sequencing from the tumours of all four strains (n=453 new tumour transcriptomes), as well as functional-genomic, digitised histopathology, and full metadata.

In sum, this collection of rich, deeply replicated, and complementary genome-wide datasets, all generated within a highly controlled and carefully documented model system, represents a landmark dataset with broad impact and will be widely reused.

In humans, there are very clear examples of how genetic background determines tumour evolution in patients: take tumours arising in Lynch, FAP, VHL or even BRCA carriers for example (compared to sporadic tumour counterparts).
Further, also in human, there are previous detailed investigations of the molecular/evolutionary differences between the multiple tumours that arise synchronously in these high-penetrance predisposition syndromes (i.e. human tumours with an identical genetic background, and essentially identical microenvironment),
such as in FAP in colon: https://www.nature.com/articles/s43018-024-00831-z (and multiple other papers)
VHL carriers in kidney: https://genomebiology.biomedcentral.com/articles/10.1186/s13059-014-0433-z and a recent bigger study that is presumably in review? https://aacrjournals.org/cancerres/article/83/7_Supplement/133/721425/Abstract-133-Rewinding-the-tape-of-life-somatic
BRCA carriers with bilateral breast cancer: https://www.nature.com/articles/s41591-023-02216-8
These studies collectively show that quite big differences in tumour evolution occur between tumours arising from essentially-identical starting points.
There is also a big literature on how human genetic ancestry (ethnicity) influences cancer biology. Perhaps prostate cancer is most instructive, where the somatic genetics of prostate cancers arising in black vs white men show stark differences, tumour biology is different, and there is correlated, probably explanatory, germline variation in the androgen receptor.

We agree that this is an area of great interest and much phenomenology has been described in the cancer epidemiology literature. In the revised manuscript we have more clearly contextualised how our work complements the existing literature, adds new mechanistic insights, and helps to frame new questions for future studies.

The work highlighted by the reviewer falls broadly into two groups. First, people with germline mutations, resulting in high-penetrance single gene (Mendelian) effects where the mechanism is known; for example, *BRCA1* mutations lead to defective double-strand break repair and therefore increased risk of cancer. Tumours arising people with such cancer predisposition syndromes [11–14]are overwhelmingly driven by high-penetrance single-gene effects that can mask more subtle polygenic or environmental contributions[15,16]. It is apparent (including references listed by the reviewer) that there can be varying penetrance between patients[17] but it can be challenging to establish the causal basis for this because these studies are often intrinsically limited by numbers of synchronous/metachronous tumours and/or number of affected family members (e.g. the highlighted FAP study[18] includes only six patients).

The second body of literature relates to how genetic ancestry or ethnicity influence cancer biology; these retrospective human cohort studies are constrained by the inherent variation of human life, including diet, sex, environment, mutagen exposure, and sociodemographic history making it difficult to identify specific risk mechanisms. Two studies from the same consortium exemplify these difficulties: kidney cancers from globally distributed cohorts showed substantial geographic variation in mutational progresses[19], whereas they found that in oesophageal cancers there were no differences in mutation burdens or signatures despite widely different incidence rates[20].

As noted by the reviewer, prostate cancer outcomes show geo-ethnic disparity[21] and parallel observations have been made in breast cancer[22], highlighting the real-world need for a mechanistic understanding of these observations. In most cases, more aggressive prostate cancers possess *CDK12* inactivation associated genomic complexities characterised by recurrent tandem duplications that involve an upstream enhancer of the androgen receptor - thus making them resistant to standard antiandrogen treatment[23]. In contrast, our mouse genomes do not show such complex karyotypes (**Fig. 3f,g**). A recent prostate cancer study identified potential pathogenic variants for germline testing in men of African ancestry[24], but this includes a large number of unvalidated candidates from a small study. This highlights the timely relevance of our manuscript and the unique power provided by our experimental design to consider each variable independently, and to identify modes of oncogenic divergence such as mutation load, spectrum, gene expression, and selection.

These latter population-based studies are more comparable to our current manuscript, where we consider tumours arising in individuals (mice) with accumulated polygenic divergence. The conflicting findings from epidemiological studies[19,20] reinforce the importance of performing experimental, *in vivo* studies in order to tease out germline vs somatic interactions in cancer - an equivalent dataset is essentially impossible to curate in human populations. Importantly, our experimental design allows us to control for all environmental factors and the nested nature of our data allows us to confidently separate the effects of individual, family, and genetic background on tumourigenesis.

Our results provide important conceptual advances. For example, an identical driver mutation in one pathway (e.g. *Braf* in the MAPK pathway) has different effects depending on genetic background, including on oncogenic p53 signalling or the occurrence/tolerance of whole genome duplication. With increasing adoption of genomics in clinical diagnostics and oncology practice, we highlight that "just" identifying a specific driver mutation may not be sufficient for prognostication and prediction, and could explain differences in outcome between patient groups[21,22].

Specific comments:

1. Fig 1A reports differences in tumour latency between strains, that appear to correlate with the evolutionary divergence between strains. I would really like to learn why from this manuscript!

Indeed, our study offers important insights into this very question. The relative resistance of wild-derived mice (*Mus musculus castaneus* strain and *Mus Caroli* species) to carcinogens and pathogens has previously been documented but is not well understood[7,25]. Here, we have shown that the differences in latency do not reflect differences in mutation burden or the spectrum of mutations generated in the livers of DEN exposed mice. The pathways acquiring activating mutations are the same and transcriptional perturbation of the key MAPK pathway is highly consistent across tumours regardless of strain. Despite this, our new analyses show there are strong epistatic interactions between the specific driver mutation and genetic background, impacting key signalling pathways (e.g. p53) (**Fig. 6c-f**), which may explain aspects of latency/susceptibility. In addition, we find profound

differences in multiple aspects of oncogenic selection which also align with strain and correspond to tumour susceptibility and latency (**Fig. 7, Extended Data Fig. 7, Extended Data Fig. 12d**).

This provides a strong argument that polygenic divergence can profoundly shape both the selection landscape and regulation of key pathways through epistatic somatic-germline interactions. These concepts motivate new thinking around the interpretation of human population variation, divergence between sub-groups, and how consistently cancer develops. While we do not claim to have answered all aspects of this question, we have provided compelling answers to parts of it, in a well structured, original, and innovative study.

2. Fig 2A reports that the mutational consequence of DEN exposure varies between strains, with the DEN2 signature enriched in C3H strain. The authors should investigate why this is. There is a comment about the role of MGMT expression but no data are shown. I think the authors have an opportunity to explore their RNA data, germline variants and chromatin state of the MGMT pathway, and/or other DNA repair pathways, etc.

Thank you for this valuable analysis suggestion. Addressing the reviewer's comment, we explored the expression of *Mgmt* in the DEN-induced tumours, normal liver at the developmental time of DEN exposure (P15), and in normal adult liver (**Extended Data Fig. 1c-e**). This demonstrates that *Mgmt* is expressed at significantly lower levels in C3H and BL6 compared to CAST and CAROLI across all developmental stages and DEN exposures. Since Mgmt is a suicide enzyme, low expression levels could allow the repair pathway to become saturated by the large number of O6-ethG adducts caused by DEN, resulting in a higher rate of the G→A (or complementary C→T) mutations that are characteristic of the DEN2 signature (**Extended Data Fig. 1b**). Incorporating these mechanistic insights, the revised text of the results section now reads:

"The DEN2 signature contributes a significant minority of the total base substitutions in all four strains, with a median of between 11.8% (CAROLI) and 14.1% (CAST), but there are notable outlier tumours in C3H and BL6 which have >30% mutations attributed to DEN2 (14% and 7% of tumours, respectively; ***Fig. 2b-d; Extended Data Fig. 1c****). O6-ethG adducts are the likely source of the G→A (or reverse complement C→T) mutations that dominate the DEN2 signature (Aitken et al. 2020), and can be directly repaired by the enzyme Mgmt (O6-methylguanine-DNA methyltransferase). Since each Mgmt enzyme can only repair a single adduct (Christmann et al. 2011), this system can be saturated. Mgmt is expressed at much lower levels in C3H and BL6 than the other strains in normal tissue, including at the time of DEN exposure (****Extended Data Fig. 1d,e****), indicating that the outlier tumours with >30% DEN2 signature may represent cases where this direct repair system was overwhelmed, resulting in an excess of O6-ethG induced mutagenesis."*

3. The analysis of mutation burden feels quite limited. I think there is a missed opportunity to explore the distribution of mutations around the genome, and correlations with epigenetic state (same for driver analysis). There is some of this analysis in fig5 and figS6 but it is limited to a global analysis of promoters, enhancers and CTCF sites. Perhaps this genome-wide heterogeneity in mutation susceptibility mechanistically explains some of the inter-strain variability in burden.

We previously presented the mutational data from the C3H DEN-induced tumour samples, which have been studied in detail with damage strand resolution from whole-genome to the single nucleotide scale, stratifying by replication time, replication strand bias, transcription strand, position within a gene, chromatin state, accessibility, and transcription factor binding[1–3]. There are many important insights

from this work but our objective was not to repeat all these analyses across the four strains. The analyses we presented of CTCF, promoters, and enhancers served to show that the DNA damage and repair dynamics that we reported for C3H are conserved and essentially identical between each of the four strains; for example, DEN-induced mutation rates are consistently elevated at CTCF binding sites across strains (**Extended Data Fig. 8g**). By classifying sites as functionally conserved or shadow (partially conserved) we extend that analysis by showing the elevated mutation rate is causally dependent on that functional activity.

Analysis in **Fig. 5** explicitly models driver mutation rates for each strain taking into account locus specific gene expression, and DNA damage strand, which shows that heterogeneity in mutation susceptibility, while clearly influential in the broader incidence of driver mutations (e.g. *Braf* V637E > *Egfr* F254I in all strains) does not account for more subtle differences in diver incidence between strains (**Fig. 5**; **Extended Data Fig. 6b-e**). In contrast, epistasis between somatic driver mutation and germline genome gives rise to strain specific perturbations of key cancer signalling pathways (**Fig. 6d-f**) which mechanistically could explain the inter-strain variability in driver incidence and cancer susceptibility.

With limited space, we think it is more important to focus the narrative on new insights from the extended gene expression analysis (as suggested by this reviewer) rather than highlighting the conservation of previously reported correlations between mutation rate and epigenetic state. The revised manuscript now mentions the broader context of previous work on the interactions between damage induced mutations and the genome regulatory landscape, summarising the cross-strain conservation and pointing interested readers to more detailed **Supplementary Text** and corresponding figures.

4. The frequent whole genome doubling (WGD) in the CAROLI strain is interesting; also that this mouse has more frequent and distinct CNAs. Again, there is no exploration of what might cause these differences. What are the germline variants in CNA regions that are divergent between lines? Are these explanatory? There may even be "pathogenic" germline variants in these regions.

We agree that the strain specific WGD and its joint dependence on a specific somatic driver mutation (*Braf* V637E) is a very interesting finding that serves as an excellent example of germline interaction with somatic mutations in cancer development. We have further explored the association of WGD with CNAs to separate out the independent effects of strain and WGD on (secondary) aneuploidy rates (**Fig. 3e**). This demonstrates that it is WGD (rather than strain itself) that pre-disposes to secondary aneuploidy - a mechanistic insight which we now consider in the results and discussion sections.

Addressing the "What are the germline variants" part of the question: The number of DNA sequence changes accumulated over evolution between the strains, as indicated by the branch-lengths based on protein-coding 4-fold degenerates sites (**Fig. 1a**) precludes practical consideration of individual germline variants: Between C3H and BL6 there are expected to be 7.5×10^6 single nucleotide differences genome-wide. Between BL6 and CAST 2.5×10^7 single nucleotide differences are expected. For comparison, between any two randomly selected humans there are expected to be between 6.4×10^6 and 2.5×10^7 single nucleotide differences (**Methods**, "Genomic annotation"), placing most human comparisons between BL6:C3H and BL6:CAST in the number of genetic differences.

As we are working with inbred mouse lines the study design does not allow biased germline allele retention analyses to be performed, since both paternal and maternal haplotypes are the same.

However, our strain-comparative analysis of copy-gain and copy-loss events across the genome shows conservation of effect direction across the genome (e.g. chromosomes 2 and 15 are preferentially gained in all strains). This seems to point to gene-dosage rather than mutation content biases in retention (e.g. the *Myc* gene on chromosome 15 is similarly prone to copy-gain events in human cancers, see page 8 line 213).

None of the driver mutation co-occurrence or mutually-exclusive relationships (**Fig. 4b,c**) with copy-gain or copy-loss events coincide on the same chromosome (e.g. chromosome 4 loss is mutually exclusive with *Hras* activating mutations, but *Hras* is located on chromosome 7 not chromosome 4). This argues against two-hit processes influencing copy number or allele ratios of induced mutations. We have commented on *Stag2* and *Amer1* loss-of-function mutations and, since these genes are both on the X chromosome, a loss of function mutation will be a complete knockout in the induced tumours (all DEN treated mice were male).

To further explore the mechanism of WGD, we have added new analyses of telomere length in normal liver and the DEN-induced tumours. This reveals that CAROLI, the only strain to undergo WGD, has the shortest telomeres in normal liver tissue and is the only strain to show significant telomere shortening in tumours (**Extended Data Fig. 3b,c**). Indeed, WGD has previously been associated jointly with telomere crisis (shortening) and telomere-damage in mice and humans, supporting a causal link (see page 8 lines 189-196).

5. Are there clues from the normal liver data about the baseline level of MAPK signalling or otherwise about why there are driver identity and number differences between strains? Are there explanatory germline variants? Are strain-biased drivers in differential chromatin state regions?

All the identified driver base substitution mutations are within bodies of expressed genes, and we have previously shown that transcription rate and transcription strand of the damage are typically the biggest influences on mutation rate in the DEN system in C3H mice[1–3]. As such, we had considered the possibility that variation in MAPK gene expression might impact driver choice. To test this, we explicitly modelled each of the recurrent MAPK pathway driver mutations in each of the strains taking into account normal gene expression in liver (P15 - the time of DEN exposure) and the strand specific mutation rates (192 category strand-resolved mutation context) from similarly expressed genes in each of the strains (see methods - "Expected mutation rate modelling"). This shows overall good concordance between observed and expected proportions of each driver mutation (**Fig. 5f**), but with some very clear outliers (e.g. *Egfr* F254I in CAROLI, and *Hras* Q61R and Q61L in C3H). Applying the same modelling, but hypothesising reduced expression of *Hras* in tumour progenitor cells, does provide a possible mechanistic explanation for the higher-than-expected *Hras* Q61L mutation rates and an improved fit for other recurrent *Hras* mutations across strains (**Extended Data Fig. 6b-e**).

The main outstanding discrepancies are the absence of *Hras* Q61L from BL6 and the absence of *Egfr* F254I from CAROLI. It seems unlikely that the absence of *Hras* Q61L represents a difference in damage or repair because (i) the same nucleotide is mutated to generate Q61R, (ii) both mutations co-occur in C3H, and (iii) the mutation signatures are closely matched between strains; instead, favouring a difference in oncogenic selection. We show that Q61L should be well presented by the MHC to the immune system specifically - and only - in BL6 (**Extended Data Fig. 7a**), providing a potential mechanism for its BL6-specific absence.

Following the reviewer's suggestion we have also looked for differential chromatin accessibility between strains over all driver mutation sites. Of the recurrent MAPK pathway mutation sites *Braf* V637, *Hras* Q61, *Hras* K117, and *Kras* Q61, are all consistently outside ATAC-seq defined peaks (accessible regions) in all replicates (3 to 8 per strain) of all strains. *Egfr* F254 is identified as overlapping ATAC-seq defined peaks in C3H, BL6, and CAROLI in single replicates but never identified as a consensus peak so chromatin accessibility is not a convincing difference between strains.

6. Fig 5 (and associated sup figures) begins to explore background gene expression and chromatin differences between strains. It would be interesting if these data were more directly related to germline genetic differences between strains.

We have extensively expanded our gene expression analysis, now incorporating the independent component analysis which dissects transcription from both tumour and normal liver (at the time of mutagenesis, P15), and compares strains (**Extended Data Fig. 10a,b** and **Extended Data Fig. 11f**).

Despite uniform transcriptional changes in the MAPK pathway, we found that the co-ordinated expression of other groups of genes - transcription signatures - correspond to specific MAPK activating mutations. Remarkably the associations between somatic driver mutations and these transcription signatures were highly divergent between strains. These epistatic interactions between driver mutation and genetic background are limited to a subset of transcription signatures that are enriched for genes involved in the p53 cellular stress response and the mutually antagonistic PPAR and TGF-β signalling pathways (**Fig. 6c-f**). Perturbation of these critical pathways would be expected to impact cell division, differentiation, inflammation and apoptosis[26–28] potentially resulting in a selective advantage, but only when the driver mutation arises on the synergistic genetic background.

This concept of somatic to germline epistasis is not currently considered in the design and validation of clinical prognostic and predictive tests. Combined with observations that specific driver mutations vary in frequency between human populations[29,30], our results indicate this is an important consideration. The same genetic change in different populations of distinct ancestry may not just reflect differences in mutagenesis [19], but could confer vastly different oncogenic consequences and clinical outcomes.

In terms of chromatin between strains, while there are some differences in conservation of promoter/enhancer marks between strains, the ChIP-seq analyses we present show that for a given group of chromatin marks (e.g. enhancers, promoters, or CTCF binding sites), the DNA damage and repair dynamics are conserved and essentially identical between each of the four strains (**Extended Data Fig. 8g**).

7. Fig 6 – I think this is the most interesting part of the paper (though ironically is less about inter-strain differences). I really like the analysis of mutations on template vs non-template strand. The analysis suggests that many driver mutations arise in cells that are not actively transcribing the oncogenic driver (because the lesions are not repaired by TCR), and therefore suggests inter-tumour differences in the cell of origin of tumours. Can the authors see this reflected in the genome-wide transcriptional phenotype of tumours? Can they see it in their digital pathology data? Alternatively, is there any evidence of inter-allele heterogeneity in expression? (could it be that expression "bursts" from one allele or other, that mean that TCR is biased to one allele or other?) Again a deeper analysis that provides more molecular mechanism would be great to see.

We agree that this is a very interesting result. To explore the higher than expected frequency of *Hras* Q61R and Q61L mutations we considered the idea that *Hras* is not actually being expressed in some progenitor cells (**Fig. 5f**). Indeed, we found the observed driver mutation frequencies could be better explained if *Hras* is not transcribed in tumour progenitor cells during the time between DNA damage and the first round of DNA replication (**Extended Data Fig. 6b-e**). This would be consistent with transformation occurring in a minority population of hepatocytes that are transiently quiescent at the *Hras* locus, which would be masked by bulk transcriptomic analysis.

The original RNA-seq analyses identified that driver gene does subtly influence tumour transcriptome (**Fig. 6b**, **Extended Data Fig. 9a-d,g**). In the revised manuscript we have added much more extensive IC analyses, and we specifically considered the influence of each driver gene and mutation on these gene expression signatures. We found that some expression signatures are enriched (or depleted) in tumours containing activating mutations in the key MAPK genes (**Reviewer Fig. 3a**). However, when considering individual *Hras* mutations, none of the driver mutations show a significant difference in expression signature enrichment (**Reviewer Fig. 3b**), indicating approximate functional equivalence of the *Hras* Q61K, Q61L and Q61R mutations. In other words, regardless of whether *Hras* is expressed or not, or which strand of the DNA is damaged, all three mutations ultimately have the same impact on gene expression in tumours harbouring these driver mutations.

In terms of pathology data: the P15 liver tissue is histologically indistinguishable between strains, with no apparent difference in cellular composition. While spatial transcriptomics analyses might allow the identification of rare cell types that are not apparent on H&E staining alone, this would not prove cell-of-origin identity. We have however used the histopathology of the resultant tumours to support our finding that the mononuclear whole genome duplication observed in mutationally symmetric CAROLI tumours occurs in the first cell division after exposure, evidenced by approximate doubling of nuclear volume (**Extended Data Fig. 3a**)

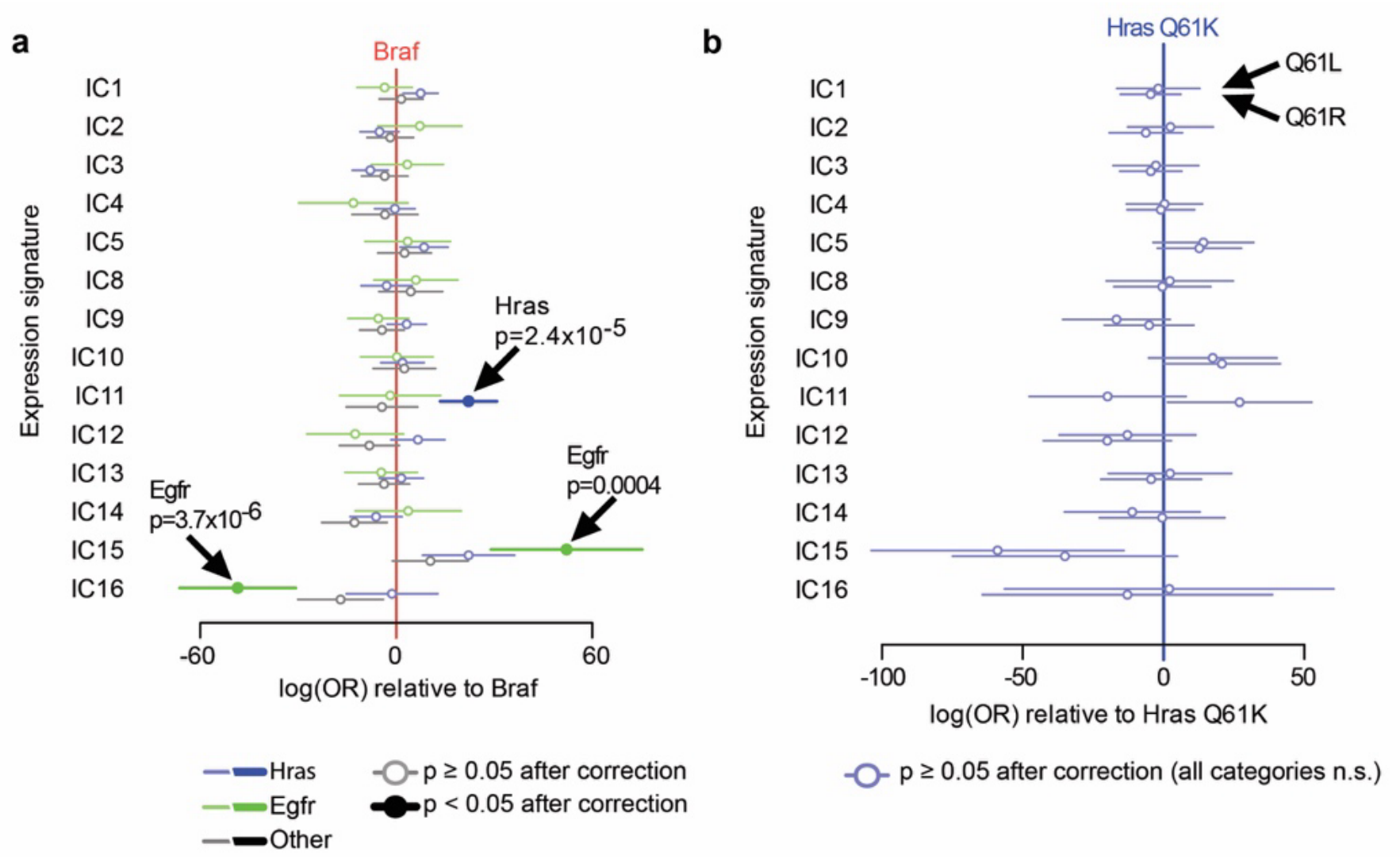

Reviewer Fig.3 | Expression signature enrichment for driver genes and mutations. Statistical analysis using multinomial, multivariable regression including strain as a model parameter. Whiskers denote 95% confidence intervals and Bonferroni corrected p-values shown for significant effects. **a**, The enrichment or depletion (positive or negative x-axis values, respectively) of tumour expression signatures for tumours containing activating mutations in the key MAPK genes *Braf*, *Hras,* and *Egfr*. Enrichments plotted as a log odds-ratio of enrichment relative to *Braf* driven tumours. Strain-specific signatures (IC6, IC7, IC17 (**Extended Data Fig. 11f**)) were excluded. **b**, The enrichment (positive x-axis values) or depletion of tumour expression signatures for tumours with *Hras* Q61 driver mutations. None of the driver mutations show a significant difference in expression signature enrichment indicating approximate functional equivalence of the *Hras* Q61K, Q61L and Q61R mutations.

8. Fig 7 – subclonal selection. The data on multiallelism show that "instant transformation" is most common in CH6 tumours: this is interesting. Could the authors explore how the retained mutagenized allele relates to driver genes (as they did in their original Lesion Segregation paper; fig2h), and possibly identify subclonal drivers this way? How is the analysis in Fig 7b affected by CNAs (WGD is already excluded I think). Please check that VAF/CCF distributions are consistent with the multiallelism data: VAF/CCF distributions should show a characteristic peak of subclonal mutations in tumours where subclonal selection is inferred. The identity of mutations in the subclonal peak can then be assessed to identify drivers.

We agree that the frequent "instant transformation" (generation 1 MRCA) of tumours in C3H versus the typically later generation MRCA in other strains is very interesting and seems to correspond to the low tumour latency and low driver mutation count of C3H - i.e. they develop tumours more readily than the other strains. The retained-mutagenised-allele analysis from the "Lesion Segregation paper"[1] is actually the basis of the Template vs Non-template transcriptional strand bias analysis for driver mutations presented in **Fig. 5d,e** and **Extended Data Fig. 6a** but instead of labelling strands "Watson" and "Crick", each locus has been oriented by the focal gene's transcription strand. A significant result in this test for an individual mutation is reporting biased strand retention. The strong and consistent lesion strand biases for driver mutations (except *Hras* Q61L) show that each of the driver mutations considered, including those in genes other than *Hras*, *Braf*, *Egfr,* and *Kras*, were typically caused by

DEN-induced damage rather than independently arising before DEN exposure or in post-damage clonal evolution.

Directly addressing the query about the effect of CNAs on MRCA generation-based estimates of selection, we have repeated the analysis from the original submission's Fig 7b considering (1) only tumours that do not contain aneuploidies, and (2) repeating the analysis for all non-WGD tumours but calculating the fraction of multiallelic segments just from the diploid, non-aneuploid portion of those genomes. Both analyses support our previous conclusion: that C3H are an outlier amongst strains with a high rate of generation 1 MRCA (**Reviewer Fig. 4a,b**).

As we have previously shown, the presence of multiallelic variation distorts VAF distributions even if all mutant alleles are considered in aggregate for the VAF/CFF calculation (see Aitken *et al.,* 2020, Extended Data Figure 6.i,j[1]). This is because accurate (non-mutagenic) replication over the lesion site reduces the mutant allele fraction in the cell population, providing a characteristic left skew to the VAF distribution specifically on chromosomes that exhibit multiallelic variation. Those left skews mimic "the characteristic peak of subclonal mutations in tumours where subclonal selection is inferred" which means the suggested checking of subclonal selection cannot be applied in this case. By extension, inferences of subclonal selection in other datasets might be detecting the influence of multiallelic variation (e.g. persistent damage induced phylogeny violating variants observed in[31]) rather than subclonal selection. It is these considerations that led us to develop the dispersion test for subclonal selection.

Considering this point about the VAF of driver mutations and identifying subclonal drivers, we have calculated the quantile position of each identified driver mutation within the VAF distribution of the corresponding tumour. For sex chromosomes, the observed VAF was divided by two, to adjust for hemizygosity; WGD tumours were excluded. Density plots of these quantile positions show that, as expected, driver mutations are biased to the right side of the VAF distribution (**Reviewer Fig. 4c-e**) - passenger mutations may be sub-clonal but the driver mutations are generally clonal. We do find driver mutations with lower VAF quantile positions, but these are biased to tumours with minimal skew to their VAF distribution (Spearman's correlation of absolute Pearson's median skewness of a tumour's VAF distribution, with driver mutation VAF quantile position, rho=0.1, p=0.001), so likely reflect stochastic sampling of a homogeneous VAF distribution. In other words, drivers with low VAF quantile positions are typically in clones with no multiallelic variation, and all DEN-induced mutations have the same VAF.

To further explore this, for each tumour we identified the recurrent MAPK driver mutations that are mutually exclusively found in almost all tumours (*Braf* V637E; *Hras* Q61R, Q61L, Q61K; *Egfr* F254I) and compared the VAF of those mutations to the VAF of identified non-MAPK driver mutations in the same tumour. This allowed 353 pairwise within-tumour driver VAF comparisons with which we asked how often the VAF of the MAPK driver was higher than the non-MAPK driver in that tumour. In 165 cases the VAF of the MAPK driver was higher, in 187 cases the non-MAPK driver was higher (and in one case the VAF was identical). This is not significantly different from the null expectation of a 1:1 ratio (Fisher's exact test odds ratio 0.88, p=0.5). Repeating that MAPK versus non-MAPK test for the subset of tumours with evidence of ongoing non-neutral evolution similarly finds no evidence for sub-clonal non-MAPK driver mutations (Fisher's exact test odds ratio 0.91, p=0.75).

We conclude that both the highly recurrent MAPK and other non-MAPK driver mutations we have identified are typically clonal rather than subclonal, although the methods originally used to identify

them were agnostic to clonality. The subclonal selection we identify with the overdispersion test may reflect the loss of deleterious subclonal mutations, or secondary driver mutations that were too heterogeneous to identify. Overall, the substitution and small indel drivers that we detect have strong selective effects on early lineages which result in defining the tumour MRCA. For example a high rate of single driver tumours is identified in C3H which also tend to have generation 1 MRCAs. In contrast strains that typically require two drivers have later generation MRCAs, which could reflect the combinatorial sampling of multiallelic variation to arrive at a cell that has both required drivers. These effects of genetic background on tumour latency and cancer susceptibility are considered in the revised manuscript Discussion.

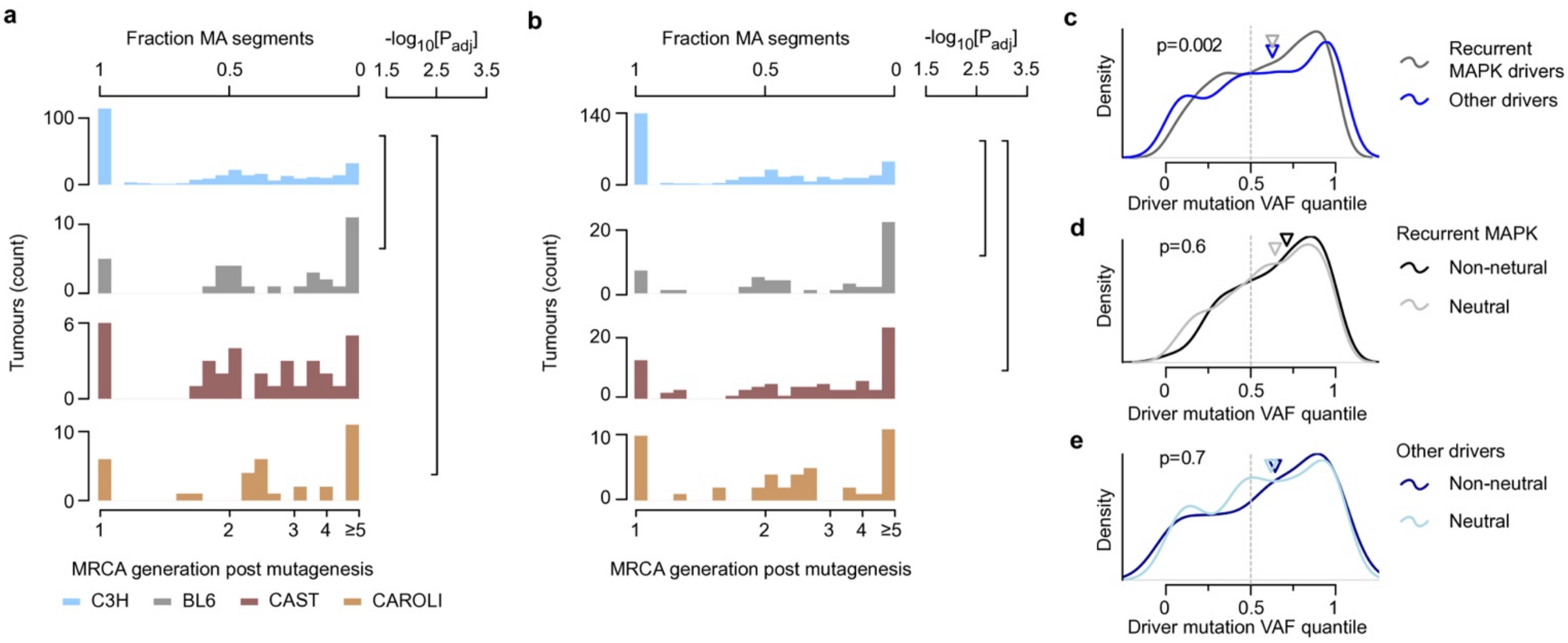

Reviewer Fig.4 | Subclonal selection. a, The fraction of chromosomal segments with multiallelic variation (top x-axis) identifies the cell-generation post mutagenesis of each tumour's MRCA (bottom x-axis); analysis restricted to tumours that do not show evidence of aneuploidy. The frequency distribution (y-axis, tumour count) of MRCA generation is strikingly different between C3H and the other strains (Kolmogorov-Smirnov test p-values, Bonferroni corrected, shown for pairwise comparisons p<0.05), with the majority of C3H tumours having their MRCA in the first cell generation post-mutagenesis. **b,** Analysis as in panel **a** but rather than excluding whole tumours with any aneuploidy, all (non-WGD) tumours were analysed but the fraction of MA segments was only calculated for the diploid, non-aneuploid portion of each tumour genome. **c,** Driver mutations are biased to higher VAF values. Within a tumour a VAF quantile of 0.5 is the median VAF, 1.0 the highest and 0.0 the lowest. The density plot shows the VAF quantile position for recurrent MAPK driver mutations (dark grey) and other non-MAPK driver mutations from non-WGD tumours (blue). Triangles denote the median VAF quantile position. Kolmogorov-Smirnov test p-values shows a significant difference in VAF quantile distribution between recurrent MAPK drivers and other drivers, primarily that the other drivers have a broader distribution of VAF quantiles. **d,** Plotted as panel **c**, demonstrating that there is no significant difference in recurrent MAPK driver mutation VAF quantile distributions between tumours with neutral or non-neutral post-MRCA evolution. **e,** Plotted as panel **c**, demonstrating that there is no significant difference in non-MAPK driver mutation VAF quantile distributions between tumours with neutral or non-neutral post-MRCA evolution.

9. Are there any distinctive molecular features of tumours with subclonal selection? Does tumour/subclonal identity correlate with strain identity and is there a potential mechanism?

As noted above (responses to reviewer questions #1.6 and #1.7), we have improved our subclonal selection analysis, and we have now included tests for associations with other key results. We show that MRCA generation is strongly associated with strain but not litter or animal (**Fig. 7d**). Across strains, late MRCA generation tumours are enriched for aneuploidies (**Extended Data Fig. 12g**), likely reflecting underlying genomic instability resulting in selective sweeps. In addition, in C3H tumours we found that first generation MRCA tumours had longer telomeres than later MRCA tumours (**Extended Data Fig. 12h**), which may also confer a growth advantage.

10. In general, it would be good to include to include more specific (quantitative) comparisons of within-strain vs between-strain heterogeneity. I appreciate some of this is already in the paper, but usually the comparison is only implicit (e.g. driver mutation burden fig 4d).

Directly addressing this point, the newly introduced likelihood ratio tests of nested mixed-effects models are now incorporated into each results section of the revised manuscript (**new Figures: Fig. 2d,e, Fig. 4e,f, Fig. 6c, Fig. 7d, Extended Data Fig. 3d,** and **Extended Data Fig. 12f,g**). These data show where variation corresponds to between strain (genetic background) or within strain variation (litter, animal or litter+animal). The multivariate regression frameworks that we have now introduced also allow us to compare effects across strains while accounting for systematic differences between strains, for example driver mutation associations on gene expression signatures while controlling for strain differences on gene expression and driver mutation frequency (**Fig. 6c-f**). We have also added formal statistical testing to comparisons of distributions between strains where appropriate (**Fig. 1b, Fig.2c, Fig.3c,e,** and **Fig. 7b**).

We think the addition of these multivariate regression frameworks bolsters the main conclusions of the manuscript and highlights both the statistical power of the study and the insights that can be derived from the novel aspects of the study design: hierarchies of genetic and environment relationships, inference of selection from multiallelic variation, and mechanistic insights into mutagenesis and selection from strand resolved DNA damage.

Minor:

11. Please add statistical tests about the differences in tumour latency between strains (fig 1A)

Latency of spontaneous tumours is now shown in **Fig. 1b** and includes Kolmogorov-Smirnov test with Bonferroni correction. We have also expanded the explanation in the corresponding Methods section to clarify how colony monitoring was performed to collect these data.

12. Please add statistical tests about mutation burden variation between strains (Fig 2B)

Genome wide substitution mutation rate is now in **Fig. 2c**. Two-sided Mann-Whitney tests with Bonferroni correction were performed between all pairwise comparisons of strains, which are now plotted. Further statistical tests of strain to mutation rate were conducted as likelihood ratio tests of nested mixed-effects models to demonstrate the importance of strain, but that there were no significant animal or litter effects, for example excluding batch effects for DEN administration per-animal or per-litter (**Fig. 2d,e**).

13. Please add statistics on inferred frequency of genome doubling, associations with nuclear size, etc throughout Fig 3.

Percentage mutational asymmetry by strain is now in **Fig. 3c**. Two-sided Mann-Whitney tests with Bonferroni correction were applied to all-versus-all pairwise comparisons of strains. We have now included additional supplementary figures to better highlight the relationship between nuclear size and genome doubling (**Extended Data Fig. 3a**).

Referee #3 (Remarks to the Author):

This original study, using a well-designed one-time DEN exposure in four mouse models with distinct varying germline genetic backgrounds, aims to advance our understanding of how germline genetic and environmental factors interact in tumor initiation and progression. As someone outside the in vivo research field, I find this particularly exciting, as such insights are difficult to achieve through epidemiological studies. However, while in vivo models offer unique advantages, caution is necessary when extrapolating and translating findings to human settings.

We thank the reviewer for their interest in our work and for their thoughtful and constructive comments on the manuscript, recognising the insights that are possible from a well-designed *in vivo* experiment. All points are addressed in our response below.

The introduction and discussion are well-structured and accessible to a wider public. However, the result section is more challenging to follow, despite its logical and stepwise presentation, with each section focusing on one part of the analyses. While a narrative is clearly present, the wider public may need a bit more guidance. Does the observed lack of differences in onco-genetic profiles between the strains suggest that the selected in vivo models are more comparable to each other and/or that other (unmeasured) factors play a larger role than expected? This is also relevant in the light of human population differences observed after specific exposures (e.g. https://pubmed.ncbi.nlm.nih.gov/38693263/). Additionally, for example, in several instances CAROLI is the significant onco-genetic profile outlier though this seems unrelated to the longer tumor latency. Given that tumor latency is likely a characteristic of the specific genetic background, further clarification on how these observations fit into the broader narrative would be beneficial.

We are pleased that this reviewer found the introduction and discussion clear and widely accessible. In the revised text, we have more clearly explained the results and their mechanistic basis, and more comprehensively contextualised them in relation to the human literature; see responses to Reviewer 2 (narrative comments before specific numbered points).

Across the four strains, the near-universal reliance on MAPK pathway activation, the shared set of non-MAPK drivers, and the common pattern of MAPK-pathway transcriptional rewiring argue that the *in vivo* models are, at a mechanistic level, highly comparable rather than idiosyncratic. This convergence is important: it shows that, despite substantial germline divergence (comparable to or exceeding major human ancestry differences), tumour evolution is funnelled into a common core oncogenic programme that closely mirrors human oncogenesis. The kidney cancer study highlighted by the reviewer[19] exemplifies why our controlled prospective study is needed: within the same consortium, kidney cancers showed strong geographic variation in mutational processes, whereas oesophageal cancer displayed no detectable differences in mutation burdens or signatures despite large

incidence differences across populations[20]. These apparently conflicting human observations, under broadly comparable exposure settings, underscore how difficult it is to disentangle environmental, germline, and somatic effects in retrospective cohorts alone. In our system, the fact that most between-strain differences in driver identity (for example, the exact *Hras* Q61 allele) and in genome-scale features (for example, mutation load, WGD, and aneuploidy) cannot be explained by mutational spectra, local sequence context or shared animal or litter effects supports a model in which germline–somatic epistasis and selection, rather than unmeasured environmental variation, are the dominant sources of divergence in tumour evolution.

Within this framework, CAROLI's behaviour is illustrative rather than contradictory. CAROLI is the most evolutionarily divergent mouse strain in our study and is an outlier for several mechanistic aspects of genome evolution, including short telomeres, high propensity to early WGD, enrichment for CNAs, and reduced SCEs. Remarkably, it still converges on the same MAPK-driven architecture as other strains and similar patterns of clonal selection seen in BL6 and CAST. Its long latency, despite pronounced genomic instability and frequent WGD, indicates that background-dependent requirements for transformation (for example, needing more than one effective driver hit) can outweigh simple metrics such as mutation burden or karyotypic disruption. Conversely, C3H's high susceptibility and short latency align with needing fewer driver events and showing more frequent "instant" (generation-one) transformation, while still using the same core MAPK circuitry.

Taken together, these observations reinforce the central narrative: different genetic backgrounds shape how tumours reach oncogenic transformation (which drivers are selected, when WGD occurs, the extent of early lineage loss), but they converge on a shared, human-relevant set of pathway perturbations. In designing this study, we anticipated that strain differences in mutation burden or spectrum would be the main drivers of divergence in the paths to tumour development, but instead our results highlight the importance of differences in selection, likely driven by epistatic interactions between specific driver mutations and the germline genome. This provides a mechanistic bridge between tightly controlled murine experiments and human population differences after shared exposures, and argues that polygenic germline context should be considered when interpreting onco-genetic profiles across populations.

This narrative is substantially reinforced by the newly introduced strain-litter-animal-tumour analysis framework and its integration with deeper analyses of gene expression. All sections of the manuscript have been revised to reflect this coherent set of findings and present them in the context of the clearer guiding narrative for the broadest possible audience.

Further specific comments on the results section:

Mostly the estimates are well-summarized in the text, which is important since these are often quite hard to read/extract from the figures. However, lines 76–85 require additional support for the reported differences in tumor latency (both DEN-induced and spontaneous), including mean ± SD values and statistical tests.

To clarify the presentation of tumour latency, updated **Fig. 1** now shows the data across two panels (**Fig. 1a,b**), and we have added statistical testing. Additionally, we have extended and clarified these details in the Methods.

Based on literature and our own prior work[32] we performed pilot experiments to identify a time point for each strain at which 100% of mice had developed tumours: 25 weeks (C3H), 36 weeks (BL6), 38 weeks (CAST), or 78 weeks (CAROLI) after DEN treatment (red diamonds in **Fig. 1a**). These time points were then implemented for all DEN experiments in the manuscript.

We also assessed the inherent susceptibility of each mouse strain to developing spontaneous tumours. To do this, untreated control mice from each strain were assessed for the presence or absence of tumours at the same ages as DEN-treated mice of the same strain (proportion of age-matched mice with tumours shown as grey pie charts in **Fig. 1a**; **Supplementary Table 3**). This identified that a quarter of the untreated C3H mice had a spontaneous tumour, but none were found in BL6, CAST, or CAROLI at the matched time point. In addition, spontaneous liver tumours were collected from untreated mice identified opportunistically during routine health surveillance of our mouse colonies (**Fig. 1b**; **Supplementary Table 3**); here, over a prolonged latency period, spontaneous tumours were identified in C3H, BL6, and CAST. In contrast, CAROLI mice remained tumour resistant even over the longer timeframe.

The Methods section now reads:
"Liver tumour samples were collected from DEN-treated mice 25 weeks (C3H), 36 weeks (BL6), 38 weeks (CAST), or 78 weeks (CAROLI) after treatment; pilot data indicated that 100% of surviving DEN-treated mice would develop tumours by these timepoints. The time of tumour harvesting was recorded for DEN-exposed BL6 mice, with 5 minute intervals added for each sequential tumour isolated (Extended Data Fig. 11a-e).

Untreated control mice from each strain were assessed for the presence or absence of tumours at the same ages to assess the inherent susceptibility to spontaneous tumours (Fig. 1a; Supplementary Table 3). In addition, spontaneous liver tumours were collected from untreated mice identified opportunistically as part of routine colony health surveillance (including one female mouse) (Fig. 1b; Supplementary Table 3). All macroscopically identified tumours were isolated and processed in parallel for DNA and RNA extraction and histopathological examination. Additional tissues from untreated P15 mice (ear, tail, and liver), untreated age-matched adult mice (liver only; C3H 27 weeks, BL6 38 weeks, CAST 40 weeks, CAROLI 80 weeks), and DEN-treated background (non-tumour) liver (BL6 38 weeks) were sampled for control experiments."

Given the genetic differences between the strains, how representative are the findings regarding the relative contributions of development, DEN exposure, and oncogenesis? Specifically, can conclusions drawn from transcriptomic data in DEN-exposed BL6 background liver (distant to tumors) be extended to the other strains (and beyond)?

To answer this question we have now included plots of the first two principal components separately for each of the four strains (**Extended Data Fig. 9a-d**). Uniformly across strains we find that the axis of oncogenesis is identified as the first principal component. For C3H, BL6, and CAROLI we reproducibly find the second principal component corresponds to developmental time as originally reported. CAST may be an outlier in this regard (**Extended Data Fig. 9c**). As this pattern is not entirely consistent between strains, in the revised manuscript we have removed mention of PC2 corresponding primarily to development.

Some specific conceptual considerations:

The study's proof-of-principle is exciting. However, caution is warranted when generalizing its findings beyond this specific DEN exposure and liver cancer model. The conclusion in the abstract—stating that "These results show that interactions between the germline genome and the environment are highly deterministic for the trajectory of tumour genome evolution, and even modest genetic divergence can substantially alter selection pressures during cancer development, influencing both cancer risk and the biology of the tumour that develops." —may be overstated.

We thank the reviewer for recognising that this is an exciting proof-of-principle study. The statement from the abstract quoted by the referee is accurate with respect to our findings of the DEN model system, which is the context in which the statement is made. However, we appreciate that this final sentence of the abstract also serves the function of signalling the broader impact of the work. In the revised manuscript, we have rephrased this statement to be less dogmatic and allow for equivocation when generalising findings beyond the model system.

The conclusion of the revised abstract reads: "*Our analyses reveal that epistatic interactions between genetic background and acquired somatic mutations result in population-specific disease progression, including choice of driver mutations, occurrence of whole genome duplication, and subclonal selection dynamics that mirror both cancer susceptibility and tumour growth rate. Even modest genetic divergence, comparable to that found within human ancestry groups, can strikingly alter selection pressures during cancer development to shape both cancer risk and the trajectory of tumour evolution.*"

In addition, we have extended the introduction and discussion sections to better contextualise our work in relation to the existing literature. Furthermore, in the revised manuscript we have identified epistatic germline-somatic interactions, whereby a specific driver mutation in the MAPK pathway can have a different effect on p53 signalling depending on the genetic background of the individual. It seems unlikely that this style of effects would be limited to the mouse genome. If such epistasis does occur in human cancers it would have important implications for genomic diagnostics - for example, it may explain divergent outcomes in precision medicine strategies despite targeting specific oncogenic mutations.

Epidemiological research indicates that genetic background and specific exposures influence cancer types and mutational profiles in well-documented ways. For example, BRCA1 pathogenic variants are strongly associated with triple-negative breast cancer (e.g. https://pubmed.ncbi.nlm.nih.gov/35084436/), while smoking is linked to specific lung cancer subtypes (e.g. https://pubmed.ncbi.nlm.nih.gov/38203395/). Admittedly, unfortunately (large-scale) studies on germline-exposure interactions remain limited and inconclusive, particularly regarding tumor genetic profiles rather than just cancer risk, largely due to sample size constraints (lack of power).

This reviewer's point nicely sums up the underlying rationale for our study which we have now specifically articulated in the introduction and discussion, including reference to several of these citations. Filling this knowledge gap, we have provided a well-powered, well-controlled study with innovative hierarchical design addressing the interactions between human-like genetic divergence and environmental carcinogen exposure. Utilising the hierarchical structure of our tumour cohort allows us to definitively show that within this system, genetic background is almost uniquely responsible for differences in mutagenesis and selection, including variance in mutation burden (**Fig. 2d,e**), oncogenic driver mutation (**Fig. 4e,f**), gene expression (**Fig. 6c**), and for measures of selection (**Fig. 7d**, **Extended Data Fig. 12f,g**), and telomere length (**Extended Data Fig. 3d**). In each case, we find the predominant

- and often only - significant covariate is the strain, which substantially reinforces the main conclusions from this manuscript - that the genetic background sets the trajectory for cancer evolution.

Furthermore, epidemiological and clinical studies have established that the impact on tumor subtypes of germline mutations depends on the mutation's pathogenicity or penetrance (even within for example high risk BRCA mutation carriers; e.g. https://pubmed.ncbi.nlm.nih.gov/25849179/). Similarly, environmental exposures have varying effects strenghts —while smoking has a strong association with lung cancer (https://pubmed.ncbi.nlm.nih.gov/12517539/), other factors, such as alcohol, have weaker relative effects on e.g. breast cancer risk (https://pubmed.ncbi.nlm.nih.gov/39581746/).

Thus, while this study provides valuable insights, its findings do not fully support broad claims about "nature's trajectory of cancer evolution." Instead, they offer strong evidence for a specific trajectory of liver cancer under a defined exposure condition. Extending these findings to all tumor types and exposures would be speculative.

We agree with the reviewer that simple extrapolation of our specific mechanistic findings to all human cancers would be speculative. In the revised manuscript we have utilised the hierarchical strain-litter-animal-tumour structure throughout the results sections (**new Figures: Fig. 1d, Fig. 2d,e, Fig. 4e,f, Fig. 6c, Fig. 7d, Extended Data Fig. 3d,** and **Extended Data Fig. 12f,g**) and we have substantially extended the RNA-seq analyses (**Fig. 6c-f**, **Extended Data Fig. 9a-d**, **Extended Data Fig. 10**, **Extended Data Fig. 11**). Combining the new expression signature analysis with the nested hierarchical relationship allows us to draw strong conclusions about epistatic interactions which set precedents that are likely to be broadly generalisable.

We have shown strong evidence of specific and reproducible trajectories of tumour development that, in defined and sometimes mechanistically explained ways, differ with the polygenic divergence between strains. For example, despite broadly uniform transcriptional changes in the MAPK pathway (**Fig. 6b**), there are pronounced epistatic interactions between somatic driver mutations and germline genetic background. These result in divergent transcription signatures, including key cancer pathways (e.g. p53, PPAR, and TGF-β) (expanded gene expression results section and **new Fig. 6b-f** and **new Extended Data Fig. 10a,b**). In other words, an identical driver mutation (e.g. *Braf* V637E, orthologous to the *BRAF* V600E mutation frequently found in human cancers) can confer a different growth/survival advantage depending on the polygenic background in which it occurs. This establishes novel mechanistic insights into how population-specific biases in driver utilisation can occur, provides a plausible mechanistic basis for strain differences in driver mutation choice and tumour latency, and may explain some of the observations from the epidemiological and clinical studies highlighted by the reviewer, for example the variable penetrance of pathogenic germline variants[17].

Specifically addressing this reviewer point we have reviewed and revised language to avoid overly broad claims. For example in the abstract "*These results show that interactions between the germline genome and the environment are highly deterministic for the trajectory of tumour genome evolution*" has been revised to "*Even modest genetic divergence, comparable to that found within human ancestry groups, can strikingly alter selection pressures during cancer development to shape both cancer risk and the trajectory of tumour evolution*." We have also been more considered when describing the interpretation of the gene expression principal component analysis, removing reference to PC2 trends that are not universal between strains.

Our original submission did not contain the phrase "nature's trajectory of cancer evolution." and we have not used it in our revised submission.

Moreover, the study's one-time high-dose exposure differs substantially from typical human exposures, which are often cumulative over a lifetime (e.g., smoking, hormones, and alcohol; e.g. https://pubmed.ncbi.nlm.nih.gov/39581746/) or repetitive due to occupational hazards (e.g., asbestos; https://pubmed.ncbi.nlm.nih.gov/39003938/). Interestingly, even in the highest risk groups there seems to be not much evidence for interaction between germline background and exposures that are known to be associated with cancer development (e.g. https://pubmed.ncbi.nlm.nih.gov/37095519/).

This raises the question whether a one-time high exposure induces a different oncogenic process compared to the durations and frequency of exposure more similar to the human situation. It While it is outside my expertise to suggest whether in vivo models could be exposed to such trajectories, at least this translational gap could be addressed in the discussion.

As the reviewer highlights, tumours arising in people with high-penetrance single-gene effects can mask more subtle polygenic or environmental contributions[15–17]. In contrast, the mice in our studies do not have such variants, allowing us to more closely model the genomic context in which most sporadic cancers occur, albeit with a strong environmental mutagen.

There are several layers of evidence to support the generalisability of our findings. We previously reported that spontaneous tumours and DEN-induced tumours harbour *Hras* driver mutations[32]. In the current manuscript, we consider other strains and demonstrate that genetic background causes the same rank ordering of susceptibility to both spontaneous and DEN-induced liver tumours (**Fig. 1a,b**). Also, while we used a single-dose DEN protocol in P15 mice, others have used serial low-doses (intraperitoneal) or chronic exposure (orally) of the same mutagen in C3H and BL6 mice (+/- a potentiating agent), in which C3H are consistently more tumour susceptible than BL6[33–35]. The same MAPK hotspot mutations are also prevalent in these studies, but genome-wide analyses are not available for comparison. To our knowledge, comparative studies have not previously been performed using CAST or CAROLI mice.

Moving from mouse to human genomes, we previously identified that single exposure of human cells to 41 different mutagens results recapitulates the lesion segregation patterns observed in this DEN mouse model (reanalysis of Kucab *et al*., datasets[36], published in Aitken *et al*., 2020[1]), and that the single base substitution (SBS) signatures are consistent with those found in their human tumour counterparts (reanalysis of ICGC[37] in Aitken *et al*., 2020[1]). We also identified genome-wide mutation asymmetry occurring in human hepatocellular carcinomas with aristolochic acid exposure (Fig. 5c,d in Aitken *et al*., 2020[1]), indicating that almost all mutations in the tumour were acquired in a single division of a cell-of-origin that was damaged from aristolochic acid. In addition, we have previously shown that single-dose UV exposure results in chromosome-scale mutational asymmetry[1,38] with the same characteristic mutations as seen in malignant melanoma[39], again highlighting the applicability of our findings to a multi-exposure scenarios akin to "real life" human situations of sporadic tumourigenesis. Finally, it is now well established that chemotherapy (typically administered in a small number of episodic short-duration high-dose exposures) is an iatrogenic cause of secondary primary cancers in later life [40] and is associated with the mutational asymmetry and multiallelic variation of lesion segregation[1,31,41].

As for all model systems, a single model cannot recapitulate the complexity and heterogeneity of all cancer behaviours. Above, we present multiple lines of evidence supporting that the conclusions drawn from our model system reflect the mutational processes that occur in multiple different exposures and clinical scenarios.

The reporting of the data and methodology is sufficiently detailed and individual data have been provided in the supplemental tables.

Thank you.

Minor clarifications:
Fig 1 a: "post partum", the method section indicates the all mice are males, should be post natal?

Thank you for catching this - we previously had a mixture of postpartum and postnatal. As suggested, the revised manuscript now only uses the term postnatal.

The data set (excel 503049_0_data_set_4644226_spvq4d) includes one female?

That is correct: there was one spontaneous tumour from a female BL6 mouse identified in colony monitoring. All control samples and DEN-treated mice in this study were male.

There are also cases of ear (or tail) instead of liver?

Yes, in addition to normal liver tissue, the ear and tail samples were used as controls to help filter out germline variation and identify regions of abnormal sequencing coverage which were masked from subsequent analyses. Ear and tail samples were not used in other analyses in this manuscript but are provided as part of the wider resource of the Liver Cancer Evolution Consortium for which this paper is intended as the primary reference.

References:

1. Aitken, S. J. *et al.* Pervasive lesion segregation shapes cancer genome evolution. *Nature* **583**, 265–270 (2020).
2. Anderson, C. J. *et al.* Strand-resolved mutagenicity of DNA damage and repair. *Nature* **630**, 744–751 (2024) .
3. Nicholson, M. D., Anderson, C. J., Odom, D. T., Aitken, S. J. & Taylor, M. S. DNA lesion bypass and the stochastic dynamics of transcription-coupled repair. *Proc. Natl. Acad. Sci. U. S. A.* **121**, e2403871121 (2024).
4. Bates, D., Mächler, M., Bolker, B. & Walker, S. Fitting linear mixed-effects models Usinglme4. *J. Stat. Softw.* **67**, 1–48 (2015).
5. Brégeon, D. & Doetsch, P. W. Transcriptional mutagenesis: causes and involvement in tumour development. *Nat. Rev. Cancer* **11**, 218–227 (2011).
6. Agapov, A., Olina, A. & Kulbachinskiy, A. RNA polymerase pausing, stalling and bypass during transcription of damaged DNA: from molecular basis to functional consequences. *Nucleic Acids Res.* **50**, 3018-3041 (2022)
7. van der Weyden, L. & Adams, D. J. Using mice to unveil the genetics of cancer resistance. *Biochim. Biophys. Acta* **1826**, 312–330 (2012).

8. Dietrich, W. F. *et al.* Genetic identification of Mom-1, a major modifier locus affecting Min-induced intestinal neoplasia in the mouse. *Cell* **75**, 631–639 (1993).

9. Haines, J. *et al.* Genetic basis of variation in adenoma multiplicity in ApcMin/+ Mom1S mice. *Proc. Natl. Acad. Sci. U. S. A.* **102**, 2868–2873 (2005).

10. van der Weyden, L. *et al.* Genome-wide in vivo screen identifies novel host regulators of metastatic colonization. *Nature* **541**, 233–236 (2017).

11. Rebbeck, T. R. *et al.* Association of type and location ofBRCA1andBRCA2Mutations with risk of breast and ovarian cancer. *JAMA* **313**, 1347 (2015).

12. Carbone, M. *et al.* Tumour predisposition and cancer syndromes as models to study gene-environment interactions. *Nat. Rev. Cancer* **20**, 533–549 (2020).

13. Breast Cancer Association Consortium *et al.* Pathology of tumors associated with pathogenic germline variants in 9 breast cancer susceptibility genes. *JAMA Oncol.* **8**, e216744 (2022).

14. Garutti, M. *et al.* Hereditary cancer syndromes: A comprehensive review with a visual tool. *Genes (Basel)* **14**, 1025 (2023).

15. Li, H. *et al.* Alcohol consumption, cigarette smoking, and risk of breast cancer for BRCA1 and BRCA2 mutation carriers: Results from the BRCA1 and BRCA2 cohort consortium. *Cancer Epidemiol. Biomarkers Prev.* **29**, 368–378 (2020).

16. Cohen, S. Y., Stoll, C. R., Anandarajah, A., Doering, M. & Colditz, G. A. Modifiable risk factors in women at high risk of breast cancer: a systematic review. *Breast Cancer Res.* **25**, 45 (2023).

17. Milne, R. L. & Antoniou, A. C. Genetic modifiers of cancer risk for BRCA1 and BRCA2 mutation carriers. *Ann. Oncol.* **22 Suppl 1**, i11–7 (2011).

18. Esplin, E. D. *et al.* Multiomic analysis of familial adenomatous polyposis reveals molecular pathways associated with early tumorigenesis. *Nat. Cancer* **5**, 1737–1753 (2024).

19. Senkin, S. *et al.* Geographic variation of mutagenic exposures in kidney cancer genomes. *Nature* **629**, 910–918 (2024).

20. Moody, S. *et al.* Mutational signatures in esophageal squamous cell carcinoma from eight countries with varying incidence. *Nat. Genet.* **53**, 1553–1563 (2021).

21. Jaratlerdsiri, W. *et al.* African-specific molecular taxonomy of prostate cancer. *Nature* **609**, 552–559 (2022).

22. Brown, L. M. *et al.* An elevated rate of whole-genome duplications in cancers from Black patients. *Nat. Commun.* **15**, 8218 (2024).

23. Viswanathan, S. R. *et al.* Structural alterations driving castration-resistant prostate cancer revealed by linked-read genome sequencing. *Cell* **174**, 433–447.e19 (2018).

24. Gheybi, K. *et al.* Pathogenic variants reveal candidate genes for prostate cancer germline testing for men of African ancestry. *Nat. Commun.* **16**, 8799 (2025).

25. Guénet, J. L. & Bonhomme, F. Wild mice: an ever-increasing contribution to a popular mammalian model. *Trends Genet.* **19**, 24–31 (2003).

26. Kastenhuber, E. R. & Lowe, S. W. Putting p53 in context. *Cell* **170**, 1062–1078 (2017).

27. Ishtiaq, S. M., Arshad, M. I. & Khan, J. A. PPARγ signaling in hepatocarcinogenesis: Mechanistic insights for cellular reprogramming and therapeutic implications. *Pharmacol. Ther.* **240**, 108298 (2022).

28. Henriques, A. *et al.* TGF-β builds a dual immune barrier in colorectal cancer by impairing T cell recruitment and instructing immunosuppressive SPP1+ macrophages. *Nat. Genet.* 1–16 (2025).

29. Carrot-Zhang, J. *et al.* Comprehensive Analysis of Genetic Ancestry and Its Molecular Correlates in Cancer. *Cancer Cell* **37**, 639–654.e6 (2020).

30. Muquith, M. & Hsiehchen, D. Landscape and saturation analysis of mutations associated with race in cancer genomes by clinical sequencing. *Oncologist* **29**, 219–226 (2024).

31. Spencer Chapman, M. *et al.* Prolonged persistence of mutagenic DNA lesions in somatic cells. *Nature* **638**, 729–738 (2025).

32. Connor, F. *et al.* Mutational landscape of a chemically-induced mouse model of liver cancer. *J. Hepatol.* **69**, 840–850 (2018).

33. Lee, G. H., Nomura, K. & Kitagawa, T. Comparative study of diethylnitrosamine-initiated two-stage hepatocarcinogenesis in C3H, C57BL and BALB mice promoted by various hepatopromoters. *Carcinogenesis* **10**, 2227–2230 (1989).

34. Weghorst, C. M., Pereira, M. A. & Klaunig, J. E. Strain differences in hepatic tumor promotion by phenobarbital in diethylnitrosamine- and dimethylnitrosamine-initiated infant male mice. *Carcinogenesis* **10**, 1409–1412 (1989).

35. Goldsworthy, T. L. & Fransson-Steen, R. Quantitation of the cancer process in C57BL/6J, B6C3F1 and C3H/HeJ mice. *Toxicol. Pathol.* **30**, 97–105 (2002).

36. Kucab, J. E. *et al.* A compendium of mutational signatures of environmental agents. *Cell* **177**, 821–836.e16 (2019).

37. International Cancer Genome Consortium *et al.* International network of cancer genome projects. *Nature* **464**, 993–998 (2010).

38. Ginno, P. *et al.* Single-mitosis dissection of acute and chronic DNA mutagenesis and repair. *Nat. Genet.* **56**, 913–924 (2024).

39. Alexandrov, L. B. *et al.* The repertoire of mutational signatures in human cancer. *Nature* **578**, 94–101 (2020).

40. Demoor-Goldschmidt, C. & de Vathaire, F. Review of risk factors of secondary cancers among cancer survivors. *Br. J. Radiol.* **92**, 20180390 (2019).

41. de Kanter, J. K. *et al.* Antiviral treatment causes a unique mutational signature in cancers of transplantation recipients. *Cell Stem Cell* **28**, 1726-1739 (2021).

Version 1:

We thank the reviewers for their unanimous support and further feedback on this manuscript. Their guidance, questions, and suggestions have been valuable in improving and refining this work. We address each of the new reviewer comments below (original reviewer text in blue, our responses in black).

Referees' comments:

Referee #1 (Remarks to the Author):

This revision is a substantial improvement. The nested animal/litter/strain model has made the data interpretation transparent. Application of ICA, and the discussion of the differential immune response to Hras Q61 mutations in BL6 have added a lot of value. I have three minor/optional comments below.

Thank you.

1) It is great to see that the authors tested their nested model in simulations. A more traditional presentation of null simulations in the form of a QQ plot and also traditional power simulations would be more convincing to a reader. The authors may decide to include simulations figures with the Extended Data. Of course, if this is difficult to do, and the authors are convinced that Type I error is well controlled, this is optional.

We now present a more comprehensive power analysis based on simulation under the strain-litter-animal-tumour hierarchical model (new Extended Data Figure 1a,b, and described in the Methods under "Statistical analyses"). This demonstrates that the type I (false discovery) error is well controlled, we have good statistical power with the parameters of our study (control of false negative, type II error) and that there is no leakage of effects between correlated variables (e.g. simulated litter effects don't manifest as significant animal effects).

2) I do not have any specific comments on presentation but find the manuscript difficult to read. I understand that this is a story about multiple observations but wonder if it can be structured better. Again, if difficult, it is fully optional.

We have made a number of edits with tracked changes to make the manuscript more concise and improve the flow and clarity. This includes moving the "Aneuploidy biases are conserved" results section to the Supplementary Text, markedly improving flow and allowing a more concise presentation of Figure 3. We have also rephrased a portion of the final results section to improve readability and narrative flow. Since results need to be presented in a linear manner through the manuscript and there are dependencies between the analyses, we think the overall ordering of sections is already optimised.

3) The manuscript (especially the discussion section) overstates the relevance of the results to human cancers. Genetic diversity in the human population is sharply different from variation amongst lab mouse strains. There is no evidence that the results are relevant to epidemiological differences between human continental populations. I do not think. this makes the manuscript of a lesser importance, because the demonstration of epistatic effects on tumorigenesis is important on its own right (I understand that some readers may only care about relevance to human cancers).

We have rephrased comparisons to human population genetic diversity through the manuscript, especially in the discussion section to make it clearer that we are not making direct claims of relevance to cancer in human populations. In places we do highlight interesting parallels between our study and what is known (and unknown) in human cancers. By providing this context, this could motivate new thinking and future translational studies on ancestry biases in cancer risk and progression.

Referee #2 (Remarks to the Author):

I continue to think that this is an interesting and high-quality paper, based on an excellent dataset with rigorous and innovative analysis. It reports strong evidence of differences in cancer evolutionary dynamics between mouse strains. The new hierarchical statistical analysis, bolstered by other specific analyses, very convincingly shows that these evolutionary differences are due to germline variation. The dissection of the evolutionary features (such as whole genome doubling, causes and timing of driver mutations, and subclonal selection) is careful, elegant and insightful.

I think my "worry" with the paper continues to be that it isn't at all surprising that germline variation causes differences in tumour evolution. The literature I cited before – from humans and mice - clearly shows this. The analysis of multiple tumours from humans with high-risk (Mendelian) predisposition syndromes also shows that there is stochasticity in the evolutionary dynamics within the same genetic background, and within the same individual/organ.

The authors argue, quite convincingly, that it is not realistic to find causal (polygenic) genetic variants that drive tumour evolutionary differences, given their study design. I accept this, and appreciate that the study I cited before from van der Wyeden (Nature 2017)- identifying host genetics that altered metastasis - has a very different design. Nevertheless I do still think that not going after *why* the germline causes differences exist between strains does limit the impact of the study essentially to an over-arching result which won't be seen as a surprise.

Nevertheless, I want to emphasise the dissection of evolutionary dynamics in the paper is elegant and innovative – particularly around timings and mechanisms of drivers and WGD – there is significant value in these data/analyses alone.

We appreciate the reviewer's support for this work and the importance of our findings of strain effects in cancer evolutionary dynamics. While it is not entirely surprising that there are genetic background differences in tumour evolution, it was surprising what those differences were (e.g. the strong selection differences and more subtle differences in base substitution mutation spectra). As for a more complete molecular description of the why differences exist - we think the extensive, well matched and well controlled data released with this manuscript will provide a fertile ground for further exploration which might be complemented by further data for example in genetic crosses or genetically engineered mouse models. This manuscript is intended to serve as the "marker-paper" for the Liver Cancer Evolution consortium data and we anticipate that it will be well cited both for its insights and for the community's wide re-use of its data.

Specific replies:
a. The inclusion of the hierarchical statistical modelling is very welcome. It does convincingly show that strain (genetic background) drives the differences between mouse strains.

b. The new correlations between gene expression phenotypes and driver frequency (Fig 6) are also welcome, they hint at some possible molecular mechanisms that drive the evolutionary differences between species.
c. I appreciate the new clarity of findings 3-8 as listed in the rebuttal.

1. I agree that the authors had definitely shown (and now do even more convincingly) that inter-strain variation is due to germline genetics. I still would like to know *what* genetics is the cause, but the authors argue strongly this is not achievable, I can (sadly) accept this is the case.
2. Good that the MGMT expression levels has now been investigated and is lower in CH3 and CAST as predicted.
3. OK – I agree with the argument made here.

Thank you - these comments and questions were very useful in improving and strengthening the manuscript.

4. Again I can accept its not reasonable to expect to find germline variants that explain CNA biases. I find the argument that there are many SNPs between strains (too many to explore!) a little weak though; it should be possible, for example, to test if there are coding variants in "important" genes located in each CNA (especially if those genes have altered expression between strains). I can imagine variants in regulatory elements of "important" genes could also be worth looking at (a nice human example for the older literature is https://www.nature.com/articles/ng.406 relating to a MYC regulating variant identified by GWAS), though I do appreciate that this analysis is more speculative.

We understand the desire to assign traits to individual germline genetic variants. In the current study design, as the reviewer acknowledges, this would be speculative. We have assigned traits to specific somatic mutations (e.g. *Braf* V637E with whole genome duplication, specifically in the CAROLI strain) which our study design is well suited to do. As noted above, the questions raised about individual germline variants, and the data we provide, are good starting points for future studies.

5. OK
6. OK
7. The reply is strong – I still find this mechanistic insight into transcription (or indeed lack of) of the HRAS is a fascinating part of the paper.

Thank you.

8. Subclonal selection analyses. I appreciate the involved new analysis of subclonal multiallelic variation. This section is very interesting and strong. The new overdispersion analysis concludes that subclonal selection is quite common. I think the authors should check what happens to this test if the death rate of the (transformed) cells is very high: this would cause (neutral) bottlenecking, and I think this could appear to be subclonal selection by the dispersion test. I do suspect the death rate following mutagen exposure is very high.

This is a very insightful point. The new simulations summarised in Referee Figure 1 demonstrate that the dispersion test readily detects random post-MRCA deaths as elevated dispersion, at least when they occur in the first few cell generations following the MRCA. This makes us appreciate that the dispersion tests would be more accurately described as measuring the uniformity of clonal expansion rather than selection/neutrality. We have rephrased the corresponding results, methods, and

conclusions accordingly. Given the sensitivity of the dispersion test to either random or non-random cell death we were surprised that the majority of tumours showed uniform clonal expansion (77±8%) suggesting that once an MRCA is established there is perhaps surprisingly little random, or selection imposed lineage loss.

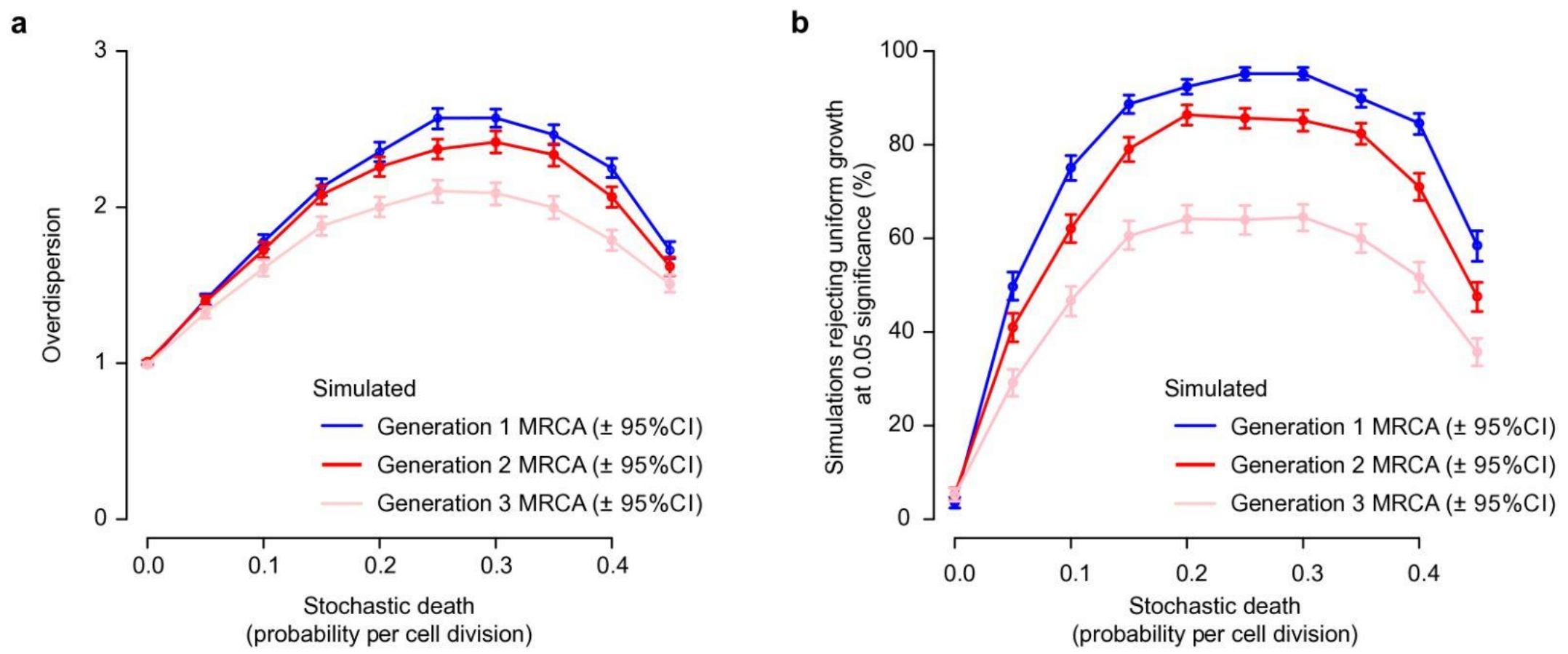

Referee Figure 1 | Simulated growth from MRCA with stochastic death. a, The effect of random cell death on estimates of overdispersion. Simulations were performed as previously described (Extended Data Fig. 12) but rather than simulating selection, random stochastic cell death was simulated for a range of cell deaths per division (x-axis). Whiskers show 95% confidence intervals. As with scenarios of extreme selection, high rates of random cell death (>0.3) effectively change the MRCA generation, resulting in reduced dispersion **b,** Simulations compared to the null expectation of uniform growth from the MRCA. The percent of simulations rejecting the null expectation at a 0.05 significance threshold (y-axis) for different rates of stochastic cell death per cell division (x-axis). Whiskers show 95% confidence intervals.

9. OK
10. The new likelihood ratio tests are great.

Minor: all fine (thanks)

Thank you.

Referee #3 (Remarks to the Author):

This manuscript has been substantially revised. The revisions have strengthened the manuscript and clarified (seeming) inconsistencies. I also highly appreciated the considerations and clarifications provided in the rebuttal and have no further questions or suggestions to offer based on my expertise.

NB apologies I made a typo and had meant to capture an essence/paraphrase ('nature's trajectory of cancer evolution') in my comment

Thank you for all the comments, questions and feedback, we greatly appreciate it.

Version 2:

We thank the reviewers for their unanimous support for the publication of our manuscript. We address each of the reviewers' final comments below (original reviewer text in blue, our responses in black).

Referees' comments:

Referee #1 (Remarks to the Author):

This revision has addressed all of my remaining comments.

Thank you for your positive comments, we appreciate your time and feedback. Your suggestion in the first review to make use of the nested structure of our dataset was particularly helpful in strengthening our analyses and conclusions.

Referee #2 (Remarks to the Author):

Thanks for looking at the effect of cell death in the dispersion test - the new tempering of the results I think is appropriate.

I have no further technical comments.

I appreciate the rigour with which the authors have dissected tumour evolutionary dynamics; it is high-quality work.

I wonder if changing the title to something like "Tumour evolutionary dynamics vary by mouse strain" would help position the paper better; I don't think I will be alone in expecting to see a mechanistic dissection of germline control of tumour evolution after reading the current title of "Genetic background sets the trajectory of cancer evolution"

Thank you, we appreciate your feedback and insightful comments throughout the productive review and revision process. We have carefully evaluated the suggestion to change the title.

A concise, accurate, and high-impact title for this manuscript should reflect the central findings. We have shown that genetic background impacts multiple facets of cancer evolution, including driver selection, whole-genome duplication, somatic-germline epistasis, and subclonal selection dynamics, and so highlighting only one of these in the title would not accurately reflect the paper as a whole. Instead, our title does support the overarching conclusion that genetic background (phrasing used 27 times in the text) provides a starting landscape that strongly influences the direction of cancer evolution. We deliberately use "trajectory" to relay the overall directionality and trend for tumourigenesis, rather than implying an entirely deterministic relationship.

The suggested alternative: "Tumour evolutionary dynamics vary by mouse strain" does not align with the style of Nature titles. It lacks the conceptual hook of a good title which should showcase a general principle; it contains a domain specific phrase "evolutionary dynamics" which reduces accessibility; and it documents details of the model system rather than foregrounding key concepts or conclusions. Despite the individual concerns with the component phrases, the overall structure of the suggested title is essentially the same as our original:

“Genetic background sets the trajectory of cancer evolution”
[condition] [has an effect on] [cancer evolution]

“Tumour evolutionary dynamics vary by mouse strain”
[cancer evolution] [has an effect on] [condition]

The main difference is that the linking term “vary by” is non-directional whereas “sets the trajectory of” is directional. Since the genetic backgrounds are the starting point for re-running cancer evolution that directionality is well justified.

We would be willing to consider, for example:

- Germline-somatic interactions direct cancer evolutionary dynamics
- Genetic background sets the trajectory of tumour evolutionary dynamics
- Germline-somatic epistasis sets the trajectory of cancer evolution
- Genetic ancestry sets the trajectory of cancer evolution

However, it is our strong preference to keep the existing title: “Genetic background sets the trajectory of cancer evolution”. We think that our title is more concise, accessible, and accurate than these alternatives. It optimally summarises, without overstating, our findings and conclusions, it has not previously provided any concern, and it reflects language used throughout the manuscript.
